# Rare Acetogenins with Anti-Inflammatory Effect from the Red Alga *Laurencia obtusa*

**DOI:** 10.3390/molecules24030476

**Published:** 2019-01-29

**Authors:** Walied Mohamed Alarif, Sultan Semran Al-Lihaibi, Nahed Obaid Bawakid, Ahmed Abdel-Lateff, Hamdan Salem Al-malky

**Affiliations:** 1 Department of Marine Chemistry, Faculty of Marine Sciences, King Abdulaziz University, P.O. Box 80207, Jeddah 21589, Saudi Arabia; sallihaibi@kau.edu.sa; 2 Department of Chemistry, Faculty of Science, King Abdulaziz University, P.O. Box 80203, Jeddah 21589, Saudi Arabia; 3 Department of Natural Products and Alternative Medicine, Faculty of Pharmacy, King Abdulaziz University, P.O. Box 80260, Jeddah 21589, Saudi Arabia; ahmedabdellateff@gmail.com; 4 Department of Pharmacognosy, Faculty of Pharmacy, Minia University, P.O. Box 61511, Minia 61519, Egypt; 5 Department of Pharmacology, Faculty of Pharmacy, King Abdulaziz University, P.O. Box 80260, Jeddah 21589, Saudi Arabia; halmalki@kau.edu.sa

**Keywords:** anti-inflammatory, *Laurencia obtusa*, acetogenins, spectroscopy, Red Sea

## Abstract

Three new rare C_12_ acetogenins (enyne derivatives **1**–**3**) were isolated from the organic extract obtained from the red alga *Laurencia obtusa*, collected from the Red Sea. The chemical structures of the isolated compounds were established by spectroscopical data analyses. Potent anti-inflammatory effect of the isolated metabolites was evidenced by inhibition of the release of inflammatory mediators (e.g., TNF-α, IL-1β and IL-6) by employing Human Peripheral Blood Mononuclear Cells (PBMC).

## 1. Introduction

Genus *Laurencia* (Rhodophyta) is commonly grown in the inter-tidal rocks of warm waters [1]. An algal database indicated that 421 species were identified, while only 146 have been taxonomically accepted [2]. *Laurencia* is well known for its ability to biosynthesize a diverse array of secondary metabolites with unprecedented molecular features [3]. This genus is rich in the polyhalogenated derivatives of different of terpenoidal skeletons [4], which significantly contribute to its defense mechanisms [5]. Moreover, the halogenated metabolites possess several biological activities, e.g., antifeedant, anthelmintic, antimalarial, antifouling, antimicrobial and cytotoxicity [6].

C_15_ acetogenins are fatty acid derivatives which, terminated with enyne or allene function. The algal acetogenins are characterized by presence of halogen atom(s) and different sizes of cyclic ether ring. For instance, isolaurenidificin, bromlaurenidificin, jeddahenyne A and B, and 12-debromo-12-methoxy isomaneonene A are recently reported acetogenins from *Laurencia obtuse* [7,8]. Sci-Finder survey indicated a lack of information on C_12_ acetogenins from the genus *Laurencia*. 

In continuation with our program aimed at isolating new chemical structures and/or biologically active secondary metabolites from marine algae [7,8,9,10,11,12], a specimen of the Red Sea *Laurencia obtusa* was chemically investigated and the anti-inflammatory effect of the pure isolated metabolites was evaluated.

## 2. Results and Discussion

**Figure 1:**
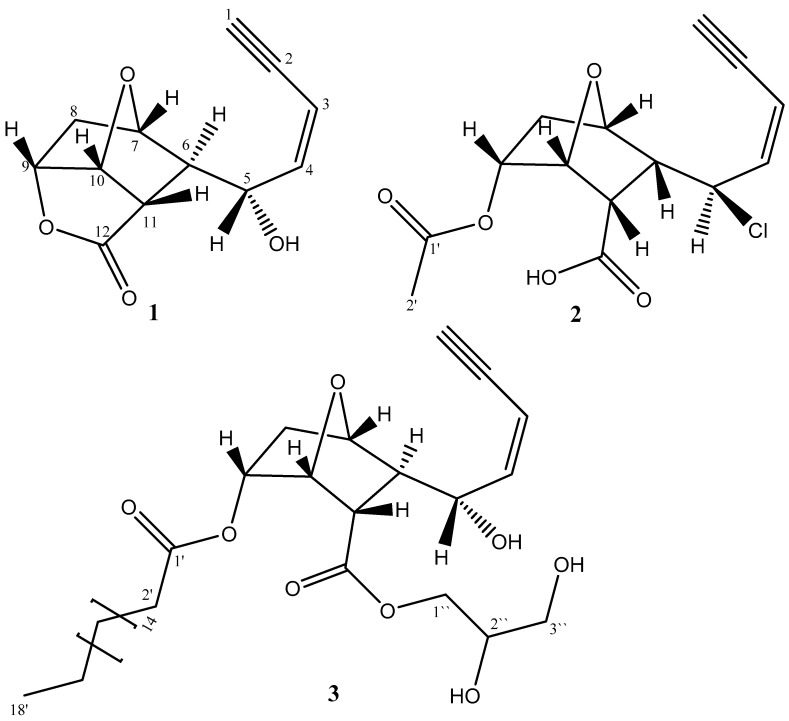
Structures of the isolated compounds **1**–**3.**

Phytochemical investigation of the organic extract of the *L. obtusa* led to the isolation of three rare C_12_ acetogenins (**1**–**3**). The chemical structures of the isolated compounds are illustrated in Figure 1. The structure elucidation was achieved by analyses of the obtained spectral data, including HRESI-MS, UV, IR, 1D and 2D NMR (see Supplementary Materials). 

Compound **1** was isolated as a pale yellow viscous material. The molecular formula of **1** was established as C_12_H_12_O_4_, based on the interpretation of HR-ESI-MS (*m*/*z* = 220.0729). The IR spectrum displayed absorption bands attributable to a hydroxyl group (3447 cm^−1^), a terminal alkyne moiety (3278 and 2171 cm^−1^), an ether functionality (1027 cm^−1^), and a lactonized carbonyl (1771 cm^−1^). The molecular formula indicated seven degrees of unsaturation. Interpretation of the DEPT and ^13^C NMR spectral data indicated the presence of a single methylene, nine methine, a carbonyl and two acetylenic carbon atoms. After association of all protons to the carbons through extensive investigation of the heteronuclear single quantum coherence (HSQC) spectral data of **1**, it was determined that the remaining proton should be assigned to a hydroxyl group. The prediction of the presence of the OH group is reinforced by the IR stretching at 3447 cm^−1^. Four degrees of unsaturation have been established through the assignment of an acetylenic, an olefinic double bond and a carbonyl function; thus, compound **1** should be tricyclic. Investigation of ^13^C NMR (Table 1) and HSQC spectral data displayed resonances for five oxygen-bearing carbons, demonstrated by the signals at δ_C_ 67.8 (C-5), 78.3 (C-7), 79.9 (C-9), 83.2 (C-10) and a carbonyl group at δ_C_ 177.7 ppm (C-12). 

Extensive interpretation of ^1^H NMR spectral data (Table 1) allowed the authors to determine the following characteristic assignments at δ_H_: 3.20 (dd, 2.6, 0.9 Hz, H-1), 5.64 (ddd, 11.1, 2.6, 1.7 Hz, H-3), 6.01 (ddd, 11.1, 10.2, 0.9 Hz, H-4); and 4.73 ppm (dd, 10.2, 10.2 Hz, H-5). 

**Figure 2:**
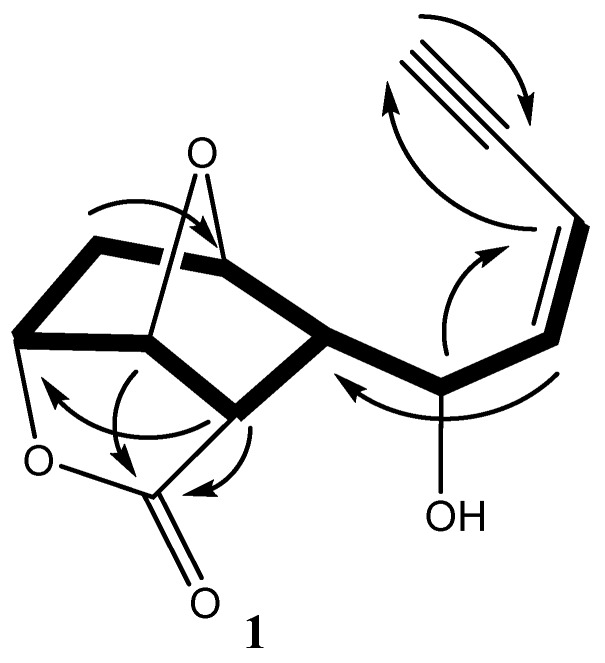
Selected COSY (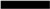) and HMBC (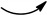) correlations of **1**.

Sequential correlations observed from H-1 to H-3 and H-4 in COSY spectrum led to the establishment of an enyne moiety (Figure 2). This deduction was consistent with the characteristic published data of the acetogenins obtained from *Laurencia* [7,8]. Further investigation of ^1^H NMR spectral data of **1** revealed that the *J*-value (11.1 Hz) between H-3 and H-4 indicated the geometry of the double bond between C-3 and C-4 to be *Z*. The low field chemical shift value of CH-5 (δ_H_ 4.73/δ_C_ 67.8 ppm) implies that such methine carbon should be attached to a hydroxyl group. Investigation of the ^1^H–^1^H COSY spectrum showed: correlations between H-6 (δ_H_ 2.59) and H-7 (δ_H_ 4.37); correlation between H-7 and H_2_-8 (δ_H_ 2.25 and 1.93); correlation between H-9 (δ_H_ 4.90) and H-10 (δ_H_ 5.37); correlation between H-10 and H-11 (δ_H_ 2.97 ppm) and H-11 and H-6. This led to the closure of the 1,4-epoxycyclohexane ring. The structure connectivity of **1** could be determined with the aid of heteronuclear multiple bond correlations (HMBC) spectral data; a cross peak of H-7/C-10 established the ether ring closure between C-7 and C-10. This deduction coincided with the published data [12,13]. On these bases, two rings have been established. HMBC correlations between H-9 and C-12, as well as, the IR absorption at ύ 1772 cm^−1^, indicated the presence of a lactone moiety; then, the third ring was established (Figure 1). 

The relative stereochemistry was assigned by performing a Nuclear Overhauser Effect Spectroscopy (2D NOESY) experiment and extensive interpretation of the coupling constant values (*J*). The *J* values ranged from 4.3 to 5.1 Hz, observed between H-6/H-7, H-7/Hb-8, H-9/H-10 and H-10/H-11 indicated a Ca 30° dihedral angle (Table 1), while values between H-5/H-6, H-6/H-11 (10.2 Hz) and Hb-8/H-9 (7.7 Hz) established a Ca 0° angle [14,15]. NOESY correlations enforced the previous deduction and established co-facial orientation of H-5, H-7, H-8b, H-9, H-10 and H-11. Compound **1**, for which the name “maneolactenol” is suggested, is a new C_12_ acetogenin.

Compound **2** was isolated as a pale yellow viscous oil. Its molecular formula was determined to be C_14_H_15_ClO_5_, based on the interpretation of HR-ESI-MS [*m*/*z =* 298.0601 and 300.0572 (3:1)]. The IR spectrum of the metabolite displayed absorptions for a hydroxyl group (3417 cm^−1^), a terminal alkyne moiety (3289 and 2171 cm^−1^), a carbonyl ester (1717 cm^−1^) and an ether functionality (1024 cm^−1^). The molecular formula of **2** indicated seven degrees of unsaturation. The interpretation of ^13^C NMR and DEPT spectral data indicated the presence of a methylene, nine methine, two carbonyl and two acetylenic carbons; accounted for five unsaturations. Thus, compound **2** has two rings. 

Further investigation of ^13^C NMR and HSQC spectra displayed resonances for four oxygen-bearing carbons; three of these carbons are demonstrated by signals at 78.8 (C-7), 78.8 (C-9), 81.6 ppm (C-10) and two carbonyl groups at δ_C_ 175.9 (C-12) and 174.9 (C-1′). The terminal conjugated enyne moiety was evidenced by interpreting ^1^H NMR spectral data, δ_H_ 3.28 (dd, *J* = 2.6, 0.9 Hz, H-1); 5.75 (ddd, *J* =11.1, 2.6, 0.9 Hz, H-3); 5.98 (ddd, *J* = 11.1, 9.4, 0.9 Hz, H-4); 4.76 (dd, *J* = 10.2, 9.4 Hz, H-5) (Table 1). This deduction was supported by ^1^H–^1^H COSY sequential correlations from H-1 to H-4. The calculated *J* value (10.2 Hz) between H-3 and H-4 indicated the geometry of the double bond between C-3 and C-4 to be *Z*. The chemical shift values δ_H_/δ_C_ (4.76/57.1) indicated the presence of halogen-bearing carbon. HR-ESI-MS exhibited a characteristic molecular-ion cluster at in a ratio 3:1, which clearly indicated the presence of a Cl atom.

Comparison of 1D and 2D NMR with those of **1** revealed that **2** contains a chlorine atom instead of a hydroxyl group. Furthermore, compound **2** is a hydrolysis and esterified product of **1**, based on the presence of acetyl and carboxylic functions in **2**. 

The relative stereochemistry was assigned by employing NOESY experiment and extensive interpretation of the coupling constants values (*J)*. The *J* values 10.2 and 7.7 Hz observed between H-5/H-6 and Ha-8/H-9, respectively, indicated a Ca 0° dihedral angle, while 5.1 Hz between H-7/Ha-8, H-9/H-10 and H-10/H-11 indicated a Ca 30° angle (Table 1). The 90° angle observed between H-6/H-7 and H-6/H-11 was due to 0 Hz coupling [14,15]. NOESY correlations reinforced the previous deduction and established co-facial orientation of H-6, H-7, H-8a, H-9, H-10 and H-11. Compound **2**, for which the name “maneolactenoate” is proposed, is a new C_12_ acetogenin.

Compound **3** was isolated as a yellow viscous material. Its molecular formula was established as C_33_H_54_O_8_, from the interpretation of HR-ESI-MS (*m*/*z* 578.3811). The IR spectrum of the metabolite displayed absorptions for a hydroxyl group (3400 cm^−1^), a terminal alkyne moiety (3220 and 2141 cm^−1^), and an ether functionality (1038 cm^−1^). The molecular formula of **3** indicated seven degrees of unsaturation. 

The interpretation of ^13^C NMR and DEPT spectral data (Table 1) indicated the presence of a methylene, nine methine, two carbonyl and two acetylenic carbon atoms. Five degrees of unsaturation were established through the assignment of an acetylenic, an olefinic double bond and two carbonyls. Therefore, **3** is a bicyclic skeleton. 

**Figure 3:**
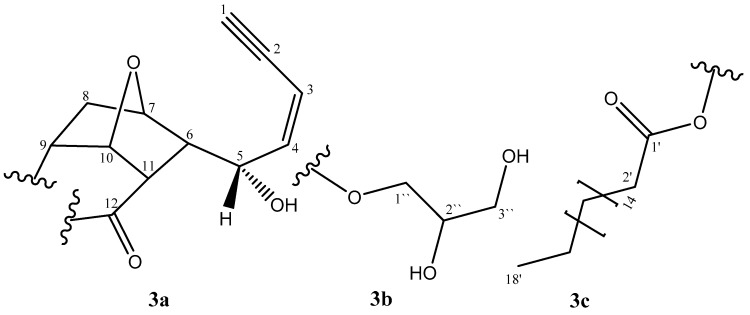
Substructures of compound **3.**

Extensive investigation of 1D and 2D NMR spectral data of **3** with those obtained from **1** indicated the coincided assignment of substructure 3a (Figure 3 and Table 1). Moreover, the NMR spectra showed a glycerol moiety assignment at δ_H_ 4.20 ppm (dd, 11.9, 4.3 Hz), 4.15 (dd, 11.9, 6.0) (H-1′′), 3.94–3.92 (m, H-2′′) and 3.70 (dd, 11.9, 4.3) 3.60 (dd, 11.9, 6.0) (H-3′′), as illustrated in substructure 3b. The only possible site to be attached as an ester moiety with carbonyl group at C-12, this deduction was supported by the HMBC correlations between H-11 and C-12 and H_2_-1′′ and C-12 through ether link. The presence of a huge CH_2_ envelope at δ_H_ 1.25, together with the signals due to fatty acid methyl end δ_H_/δ_C_ (1.25/14.1), indicated the existence a fatty acid moiety. The fatty acid is estrified with the main skeleton at C-9, supported by the HMBC correlations between H-9 and C-1′ through ether link. In order to determine the length of the fatty acid chain, the acid methanolysis method of Gaver and Sweeley [16], which yielded a FAME methyl tetradecanoate, was employed. The *m*/*z* 298 parent peak detected by GCMS analysis indicated the presence of C-18 fatty acid moiety and confirmed by the characteristic ion at *m*/*z* 267 [CH_3_(CH_2_)_16_CO]^+^. Thus, the molecular formula of FAME was shown to be C_19_H_38_O_2_. The relative stereochemistry of **3** is similar to that of **1**. The trivial name “Jeddahmaenoneoate” was given to the compound **3**.

**Figure 4:**
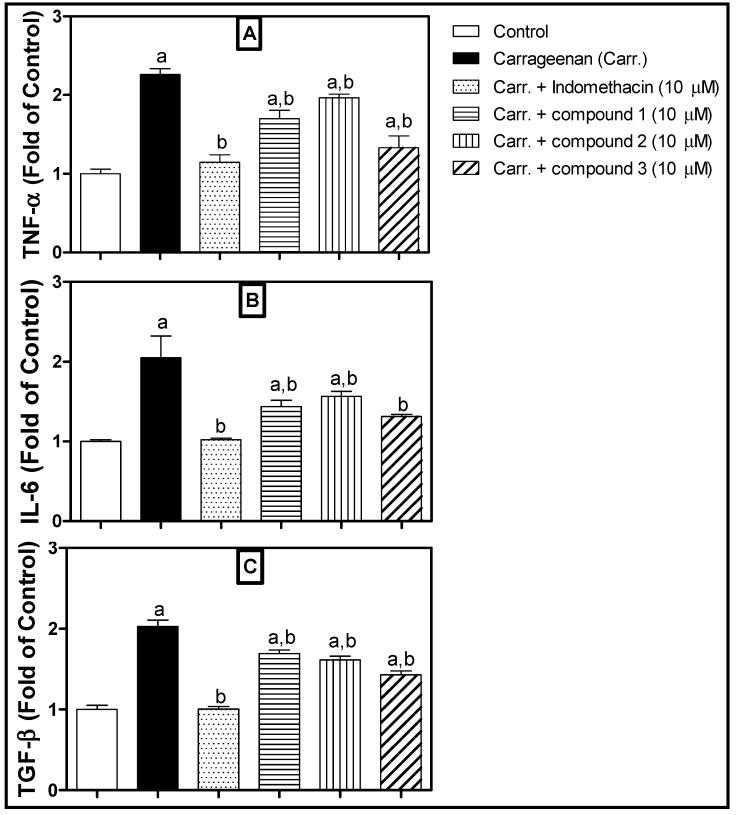
Effect of compounds (**1**–**3**) on: TNF-α (Panel **A**), IL-6 (Panel **B**) and TGF-β (Panel **C**) release in Carrageenan-stimulated PBMCs. a statistically different from the corresponding control group at *p* < 0.05; b statistically different from the corresponding Carrageenan-treated group at *p* < 0.05.

The obtained results indicated that compounds **1**–**3** (Figure 4) exhibit potent anti-inflammatory activity, as they significantly inhibited the inflammatory cytokine release from Peripheral Blood Mononuclear cells (PBMC) challenged with carrageenan. They revealed potent activity as evidenced by the inhibition of the release of inflammatory mediators like TNF-α, IL-6 and TGF-β. It is noteworthy that compound **3** exhibited the most potent effect that was very comparable to that of reference compound, indomethacin. It is proposed that the major difference in the chemical structure between **3** and other structures is the presence of a fatty chain, which plays a vital role in increasing the anti-inflammatory activity. 

## 3. Experimental 

### 3.1. General

Column chromatography was performed with Aluminum Oxide Fluka (Sigma-Aldrich, Buchs, Switzerland), neutral type 507C. Fractions were examined by TLC Silica Gel F_254_ plates. Preparative TLC glass plate (20 cm × 20 cm) supported silica gel of 250 μm thickness was used. Spots were visualized using UV light (λ_max_ = 254 nm) then detected using *p*-anisaldehyde-sulfuric acid as a spray reagent. Electron ionization mass spectra were recorded on Kratos MS-25 (Manchester, UK) at ionizing voltage of 70 eV. The 1D and 2D Nuclear Magnetic Resonance data were obtained on Bruker 850 MHz spectrometer (Bruker Company, Berlin, Germany). Samples were dissolved in CDCl_3_ (δ_H_ 7.26 and δ_C_ 77.0). 

### 3.2. Phytochemistry

#### 3.2.1. Algael Material 

*Laurencia obtusa* was collected in May 2016 from Salman Gulf, north of Jeddah, Saudi Arabia (21°51′39.8” N; 38°58′42.7” E). Reference stander (JAD 03060) was stored at the Faculty of Marine Sciences, King Abdulaziz University. The sample was identified by Prof. Mohsen El-Sherbiny, Marine Biology Department, Faculty of Marine Sciences, King Abdulaziz University, Jeddah, Saudi Arabia. 

#### 3.2.2. Extraction and Isolation

A dried sample of *Laurencia obtusa* (200 g) was extracted with a mixture of dichloromethane and methanol (1:1), yielding a dark green residue (6 g), which fractionated on the aluminum oxide column, employing gradient elution with *n*-hexane/diethylether; *n*-hexane/ethyl acetate, and finally, dichloromethane/ methanol. Fractions of 25 mL were gathered and monitored by employing the TLC technique. The promising spots were further purified by PTLC and Sephadex LH-20. 

Fraction eluted with *n*-hexane: ethyl acetate (1:1) was purified by Sephadex LH-20 with MeOH: CHCl_3_ (9.5:0.5) and then by preparative TLC system using *n*-hexane:ethyl acetate (1:1) to endow compounds **1**–**3** with R_f_ values 0.26, 0.33, and 0.11, respectively. 

#### 3.2.3. Spectral Data

Compound (**1**): pale yellow viscous oil (1.5 mg, 0.0008 % yield, based on dry weight); [α]^22^_D_= −87.25 (*c* 0.02, CHCl_3_); UV (MeOH) λ_max_ 229 nm; IR ύ 3447, 3278, 2924, 2854, 1771, 1445, 1359, 1165, 1114, 1027 and 985 cm^−1^; ^1^H NMR (CDCl_3_, 850 MHz) and ^13^C NMR (CDCl_3_, 213.77 MHz); HR-ESI-MS *m*/*z* 220.0729[M]^+^ (calcd. for C_12_H_12_O_4_, 220.0736).

Compound (**2**): pale yellow viscous material (1.8 mg, 0.0009 % yield, based on dry weight) [α]^22^_D_= −111.34 (*c* 0.02, CHCl_3_); UV (MeOH) λ_max_ 225 nm; IR ύ 3413, 3289, 2921, 2171, 1782, 1563, 1127 and 1024 cm^−1^; ^1^H NMR (CDCl_3_, 850 MHz) and ^13^C NMR (CDCl_3_, 213.77 MHz); HR-ESI-MS *m*/*z* 298.0601, 300.0572[M]^+^ (3:1) (calcd. for C_15_H_14_^35^ClO_5_, 298.0608; C_15_H_14_^37^ClO_5_, 300.0579).

Compound (**3**): yellow viscous material (1.3 mg (0.0007 % yield, based on dry weight), [α]^22^_D_= −183.13 (*c* 0.02, MeOH); UV (MeOH) λ_max_ 229 nm; IR ύ 3400, 2920, 2332, 2141, 1733, 1038 and 736 cm^−1^; ^1^H NMR (CDCl_3_, 850 MHz) and ^13^C NMR (CDCl_3_, 213.77 MHz); HR-ESI-MS *m*/*z* 578.3811[M]^+^ (calcd. for C_33_H_54_O_8_, 578.3804).

### 3.3. Biological Study

#### 3.3.1. Separation of Human Lymphocytes

Peripheral blood mononuclear Cells (PBMCs) were separated from whole blood obtained from healthy volunteers via density centrifugation. Briefly, diluted blood samples were layered onto a Ficoll-Paque reagent, followed by centrifugation at 400× *g* for 15 min (20 °C), and then the lymphocyte layer was transferred out. The cells were washed and pelted down with three volumes of PBS-BSA-EDTA. Then, they were maintained in supplemented DMEM at 5% CO2 for 48 h. 

#### 3.3.2. Lymphocyte Challenging

Separated PBMCs were seeded at a density of 1.5 × 106 cells/mL in 6-well plates. Then, the test Sexperiment. After 24 h, 10 µg/mL of mitogen “carrageenan” was utilized to stimulate the cells. The control group included cells challenged with carrageenan but not the tested compounds. An additional group was used and included unchallenged cells that were treated with DMSO in a final concentration equal to that of the test wells, but in no way exceeding 0.1%. The reference standard “Indomethacin, 10 µM” was used. After 48 h of incubation, the levels of TNF-α, IL-1β and IL-6 were determined in the supernatant media. 

#### 3.3.3. Assessment of Inflammatory Mediators

The concentration of inflammatory mediators (TNF-α, IL-6 and TGF-β) was assessed using a commercially available kit (Signosis, Santa Clara, CA, USA). Plates were coated with specific primary antibodies. After incubation, the wells were washed to remove unbound labeled antibodies. A HRP substrate, TMB, was added to result in the development of a blue color. The color development was then stopped with the addition of a stop solution, changing the color to yellow. The concentrations of the angiogenesis cytokines were directly proportional to the color intensity of the test sample. Absorbance was measured spectrophotometrically at 450 nm using a microplate reader (BioTeck ELx800, Winooski, VT, USA).

#### 3.3.4. Statistical Analyses

Data are presented as mean ± S.D. One-way analysis of variance (ANOVA) followed by Tukey’s test for post hoc analyses were used to carry out comparisons. The level of *p* < 0.05 for statistical significance was acceptable. GraphPad InStat software (version 3.05, GraphPad Software Company, Northside, San Diego, CA, USA) was used to carry out statistical analyses. GraphPad Prism software (version 5.00) was utilized to plot graphs (GraphPad Software, Inc., La Jolla, CA, USA). Data are presented as mean ± S.D. Statistical analysis was performed using one-way analysis of variance (ANOVA) followed by Tukey post hoc test. *n* = 3; a statistically different from the corresponding control group at *p* < 0.05; b statistically different from the corresponding Carrageenan-treated group at *p* < 0.05.

## 4. Conclusions

Chemical investigation of *Laurencia obtusa* led to identification of three new rare C_12_ acetogenins (enyne derivatives **1**–**3**). These compounds showed potent anti-inflammatory effect through inhibition of the release of inflammatory mediators (e.g., TNF-α, IL-1β and IL-6) by employing Human Peripheral Blood Mononuclear Cells (PBMC).

## Figures and Tables

**Table 1:** ^1^H and ^13^C NMR spectral data for compounds **1**–**3**^a–c^.

No.	1		2		3	
	δ_H_ (*J* in Hz)	δ_C_	δ_H_ (*J* in Hz)	δ_C_	δ_H_ (*J* in Hz)	δ_C_
1	3.20 dd (2.6, 0.9)	84.1	3.28 dd (2.6, 0.9)	85.4	3.21 dd (2.6, 0.9)	83.2
2	-	79.1	-	78.1	-	79.1
3	5.64 ddd (11.1, 2.6, 1.7)	110.5	5.75 dd (11.1, 2.6)	113.2	5.65 dd (11.1, 2.6)	110.6
4	6.01 ddd (11.1, 10.2, 0.9)	143.0	5.98 ddd (11.1, 10.2)	139.9	6.01 ddd (11.1, 10.2, 0.9)	143.0
5	4.73 dd, (10.2, 10.2)	67.8	4.76 dd (11.1, 10.2)	57.1	4.74 dd (10.2, 10.2)	67.8
6	2.59 dddd (10.2, 10.2, 4.3, 1.7)	53.3	2.42 dd (11.1, 1.7)	55.9	2.60 dddd (10,2, 10.2, 4.3,1.7)	53.5
7	4.37 dd (5.1, 4.3)	78.3	4.98 d (5.1)	78.8	4.37 dd (5.1, 4.3)	78.3
8a 8b	2.25 d (14.5) 1.93 dddd (14.5, 7.7, 5.1, 1.7)	35.1	2.11 m 1.80 d (14.5)	38.3	2.26 d (14.5) 1.93 dddd (14.5, 7.7, 5.1, 1.7)	35.1
9	4.90 dd (7.7, 5.1)	79.9	4.86 dd (7.7, 5.1)	78.8	4.91 dd (7.7, 5.1)	79.9
10	5.37 dd (5.1, 5.1)	83.2	5.31 dd (5.1, 5.1)	81.6	5.37 dd (5.1, 5.1)	84.1
11	2.97 dd (10.2, 5.1)	43.0	2.46 brd (5.1)	43.6	2.97 dd (10.2, 5.1)	43.0
12		177.7		175.9		174.4
1′				174.9		177.7
2′			2.09 s	20.3	2.35 t (7.7)	35.1
3′-16′					1.64–1.62 m	24.9
17′					1.30–1.24 m	29.7
18′					0.88 t (6.8)	14.1
1″					4.20 dd (11.9, 4.3) 4.15 dd (11.9, 6.0)	65.2
2″					3.94–3.92 m	70.3
3″					3.70 dd (11.9, 4.3) 3.60 dd (11.1, 6.0)	63.3

^a^ All assignments are based on 1D and 2D measurements (HMBC, HSQC, COSY). ^b^ Implied multiplicities were determined by DEPT (C = s, CH = d, CH_2_ = t). ^c^ *J* in Hz.

## Supplementary Materials

The Supplementary Materials are available online.
