# Supplementary material for: Rare Acetogenins with Anti-Inflammatory Effect from the Red Alga Laurencia obtusa
Source: Molecules. 2019 Jan 29;24(3):476. doi: 10.3390/molecules24030476 (PMC6384645; doi:10.3390/molecules24030476)
Supplement: Supplementary file 1 [file molecules-24-00476-s001.pdf]

SUPPLEMENTARY MATERIAL

Rare acetogenins with anti-inflammatory effect from the red alga *Laurencia obtusa*

Walied Mohamed Alarif [1,*], Sultan Semran Al-Lihaibi [1], Nahed Obaid Bawakid [2,*], Ahmed Abdel-Lateff [3,4] and Hamdan Salem Al-malky [5]

[1] Department of Marine Chemistry, Faculty of Marine Sciences, King Abdulaziz University, PO. Box 80207, Jeddah 21589, Saudi Arabia; sallihaibi@kau.edu.sa

[2] Department of Chemistry, Faculty of Science, King Abdulaziz University, PO. Box 80203, Jeddah 21589, Saudi Arabia

[3] Department of Natural Products and Alternative Medicine, Faculty of Pharmacy, King Abdulaziz University, PO Box 80260, Jeddah 21589, Saudi Arabia; ahmedabdellateff@gmail.com

[4] Department of Pharmacognosy, Faculty of Pharmacy, Minia University, PO. Box 61511, Minia 61519, Egypt.

[5] Department of Pharmacology, Faculty of Pharmacy, King Abdulaziz University, PO Box 80260, Jeddah 21589, Saudi Arabia; halmalki@kau.edu.sa

* Correspondence: walied1737@yahoo.com or welaref@kau.edu.sa (W.M.A.); nbawaked@kau.edu.sa (N.O.B.); Tel.: +966-56-0352-034 (W.M.A.); Tel +966-50-4690-309 (N.O.B.)

Abstract

Three new rare C12 acetogenins en-ynes derivatives (**1-3**) were isolated from the organic extract obtained from the red alga *Laurencia obtusa*, collected from the Red Sea. The chemical structures of the isolated compounds were established by spectroscopical data analyses. Potent anti-inflammatory effect of the isolated metabolites was evidenced by inhibition of releasing the inflammatory mediators (e.g. TNF-α, IL-1β and IL-6) by employing Human Peripheral Blood Mononuclear Cells (PBMC).

Keywords: Anti-inflammatory; *Laurencia obtusa*; Acetogenins; Spectroscopy; Red Sea

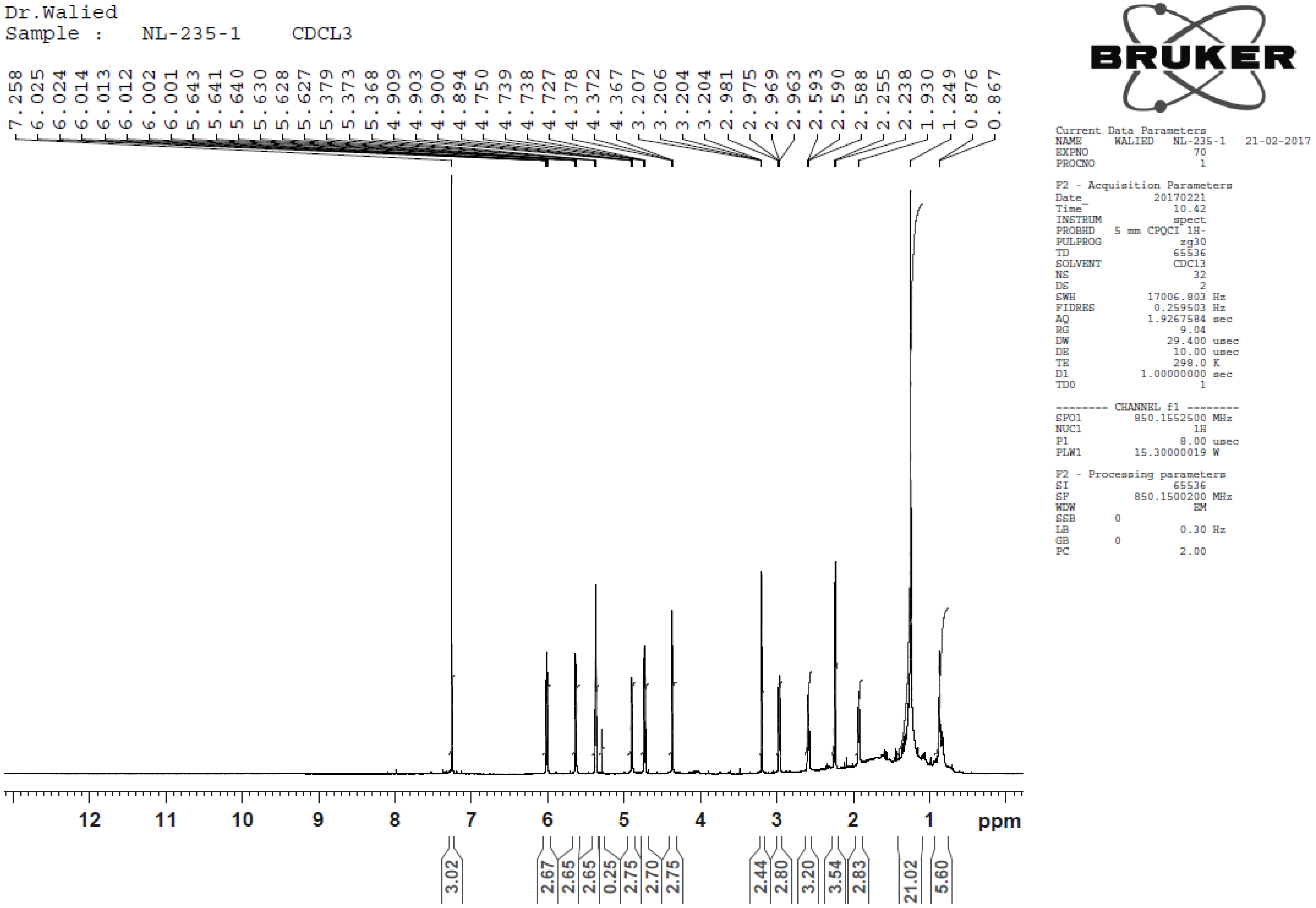

Figure S1a: ^{1}HNMR of compound 1

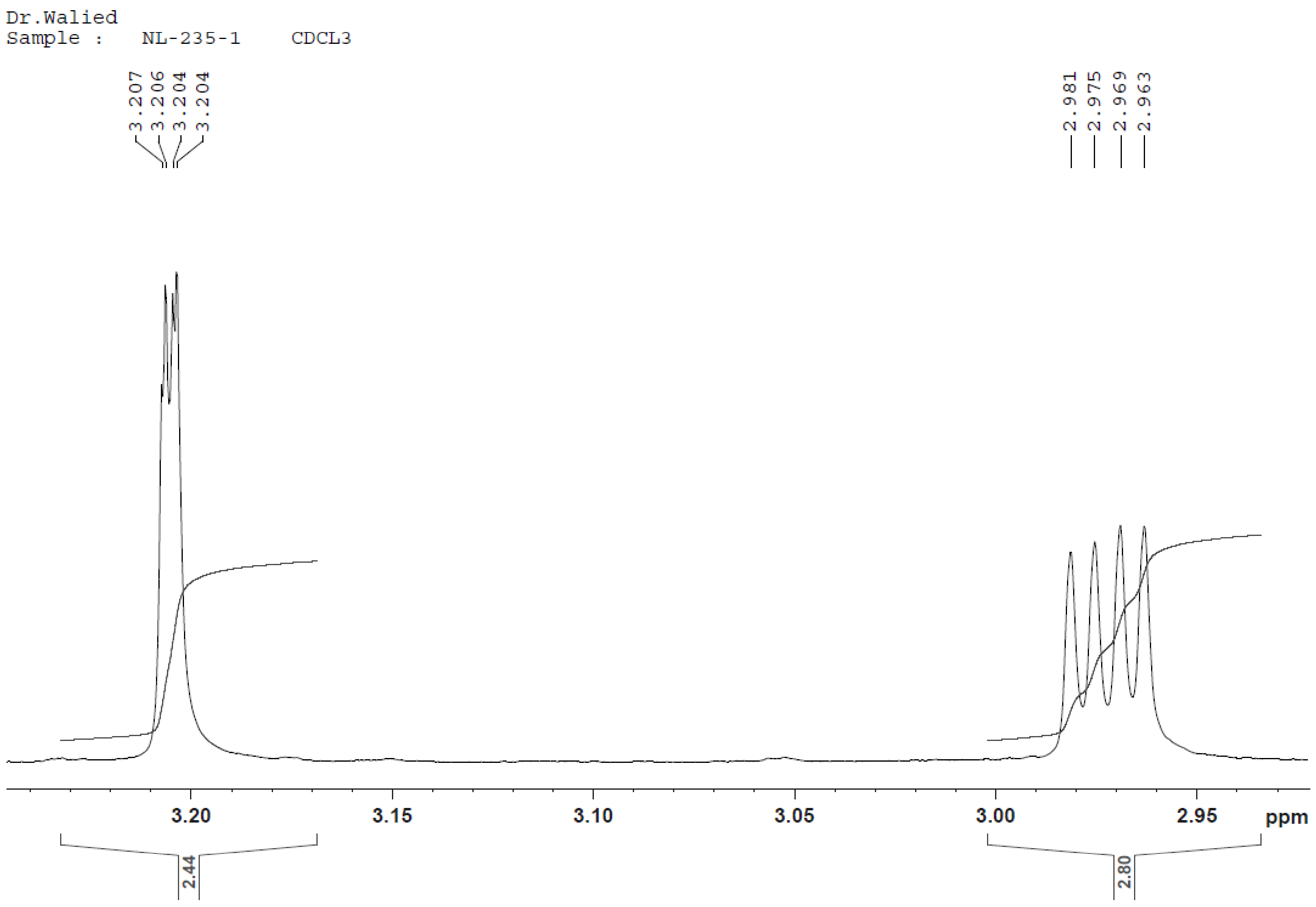

Figure S1b: ^{1}HNMR of compound 1

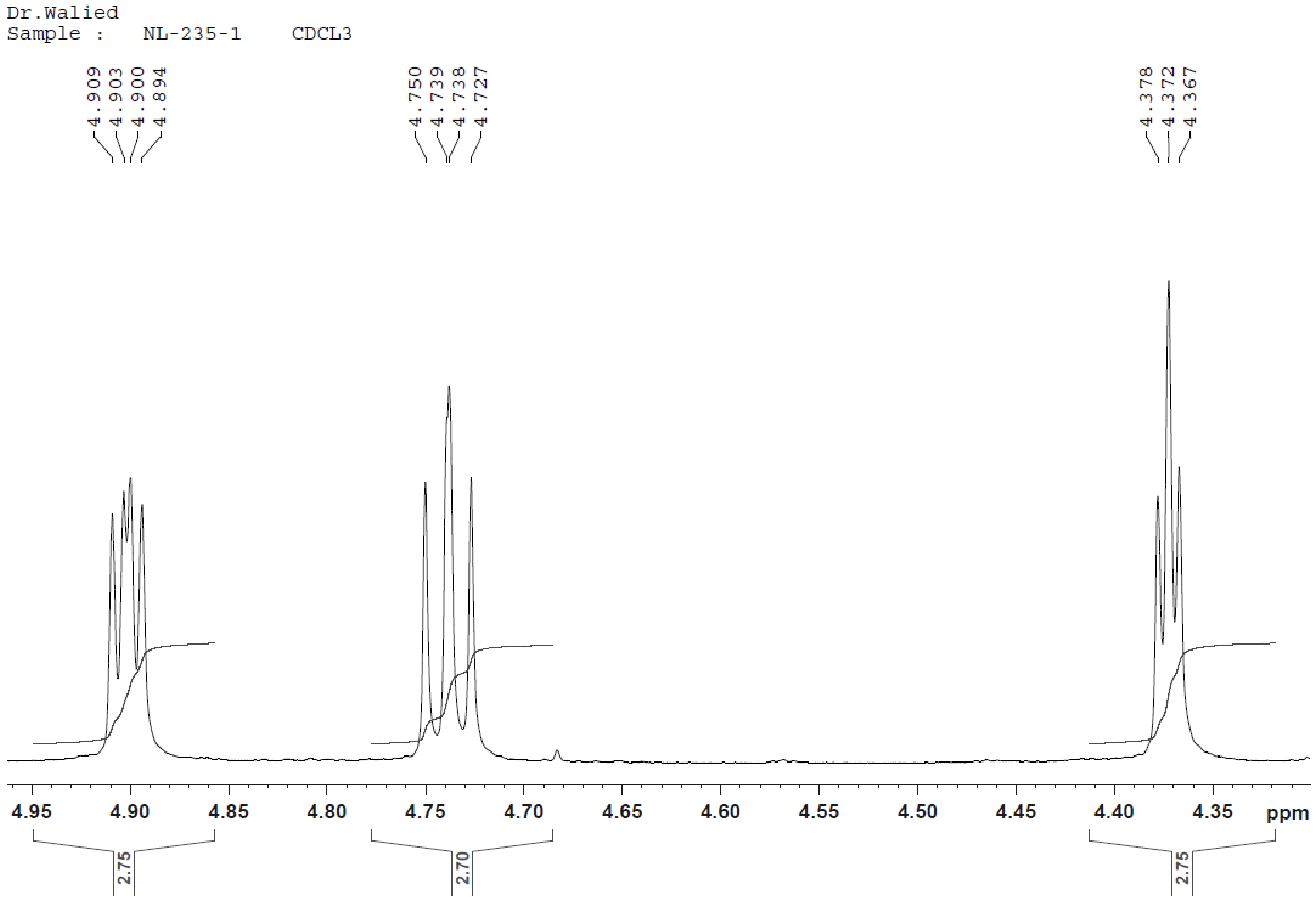

Figure S1c: ^{1}HNMR of compound 1

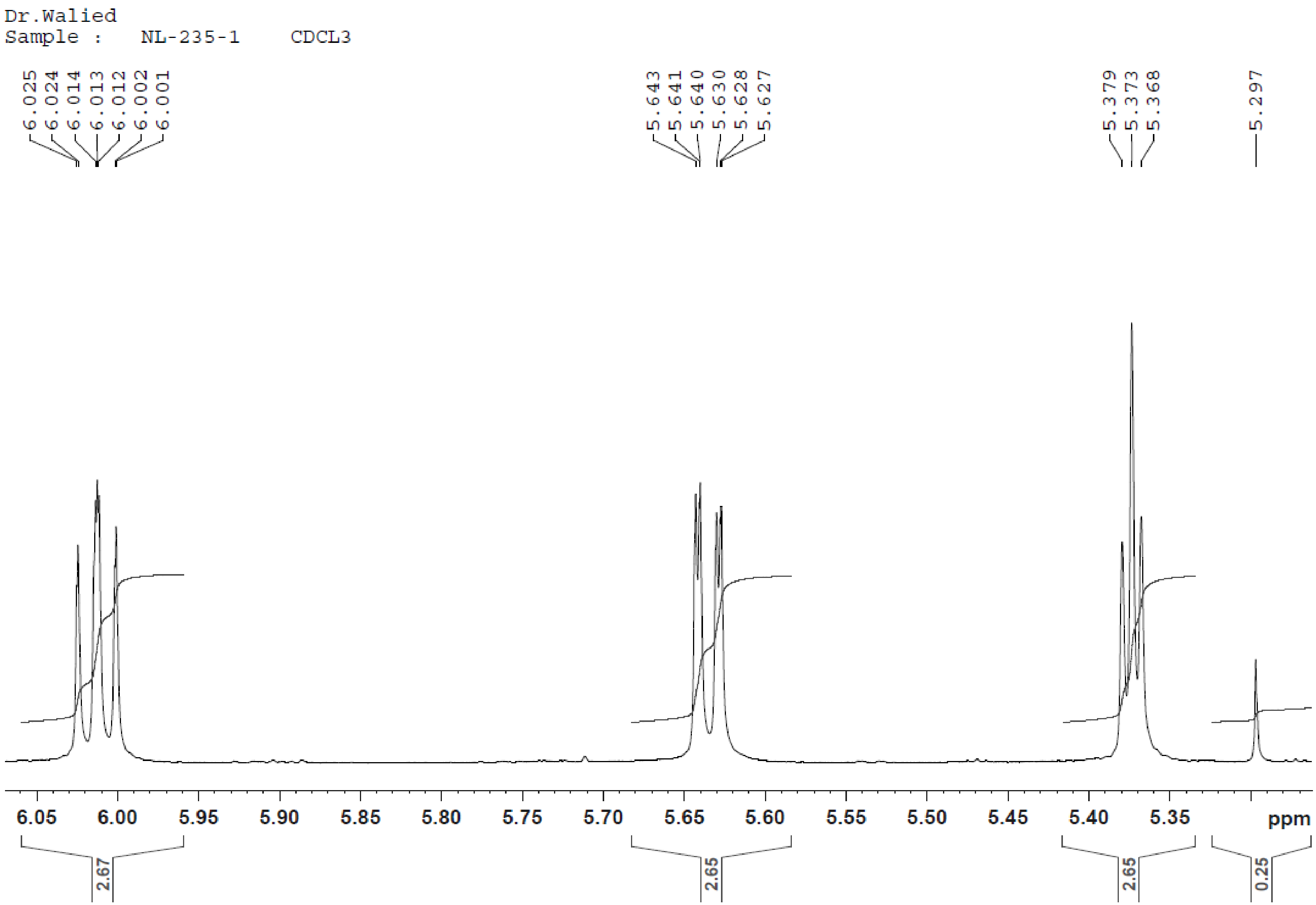

Figure S1d: ^{1}HNMR of compound 1

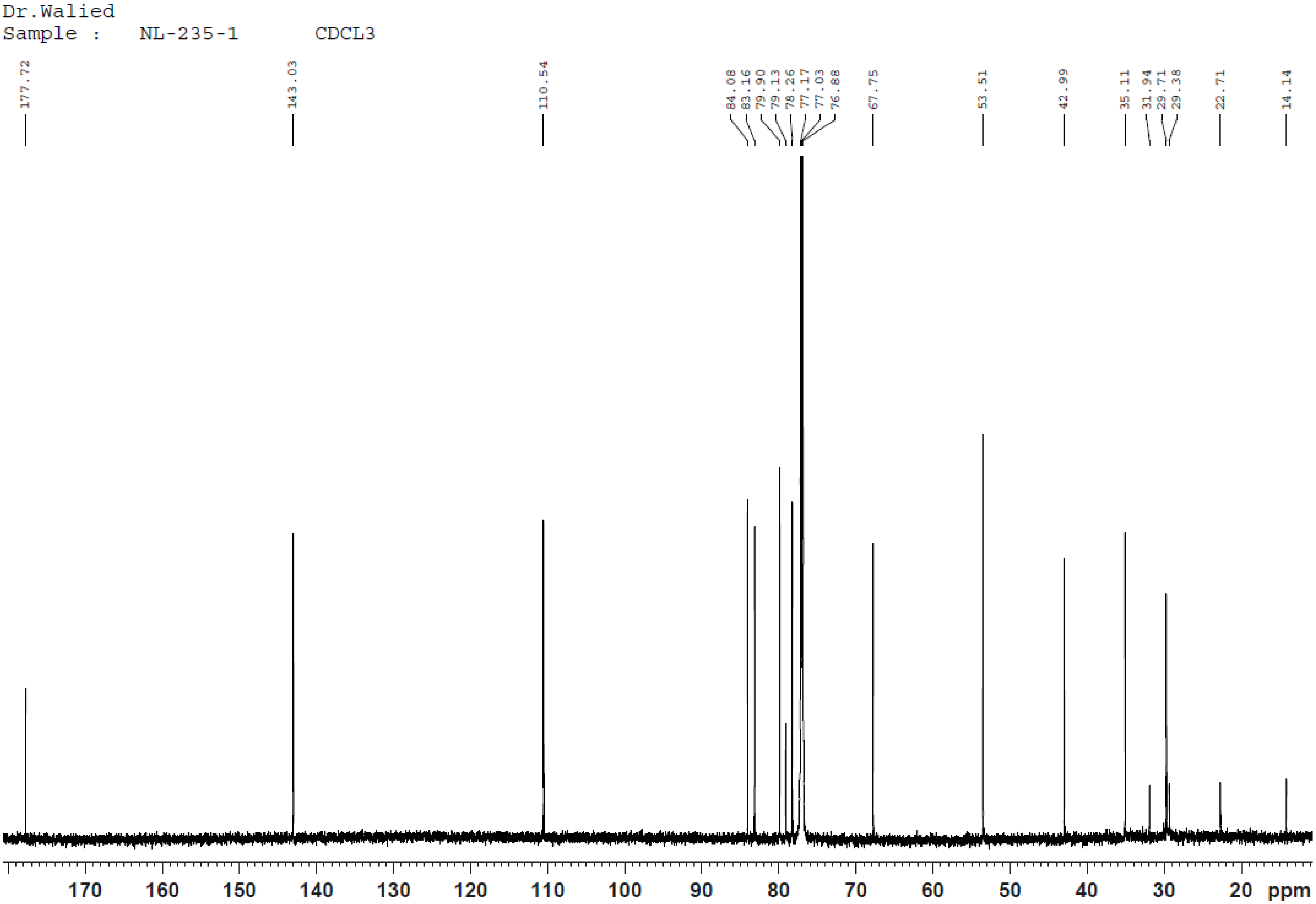

Figure S1e: ^{13}CNMR of compound 1

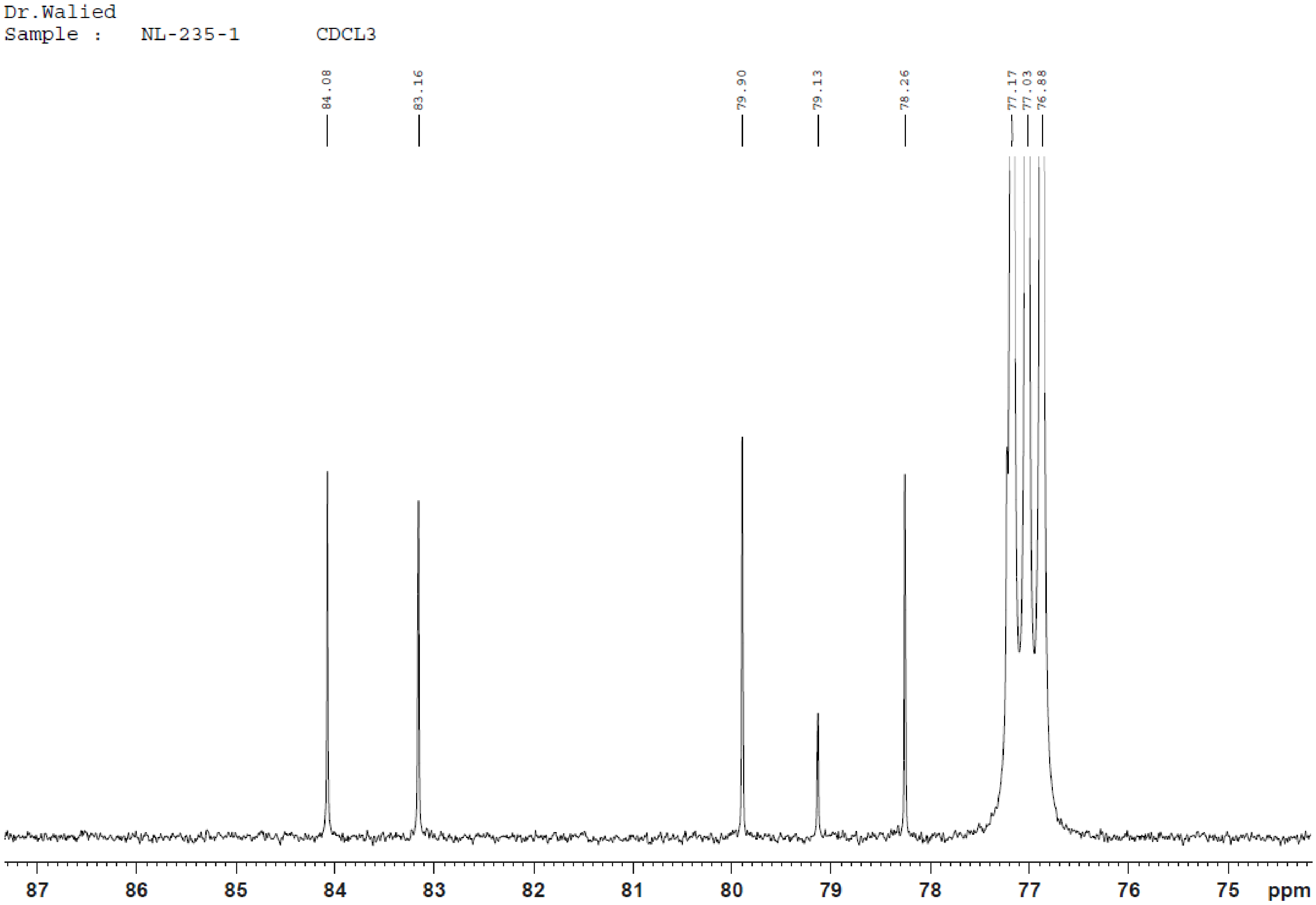

Figure S1f: ^{13}CNMR of compound 1

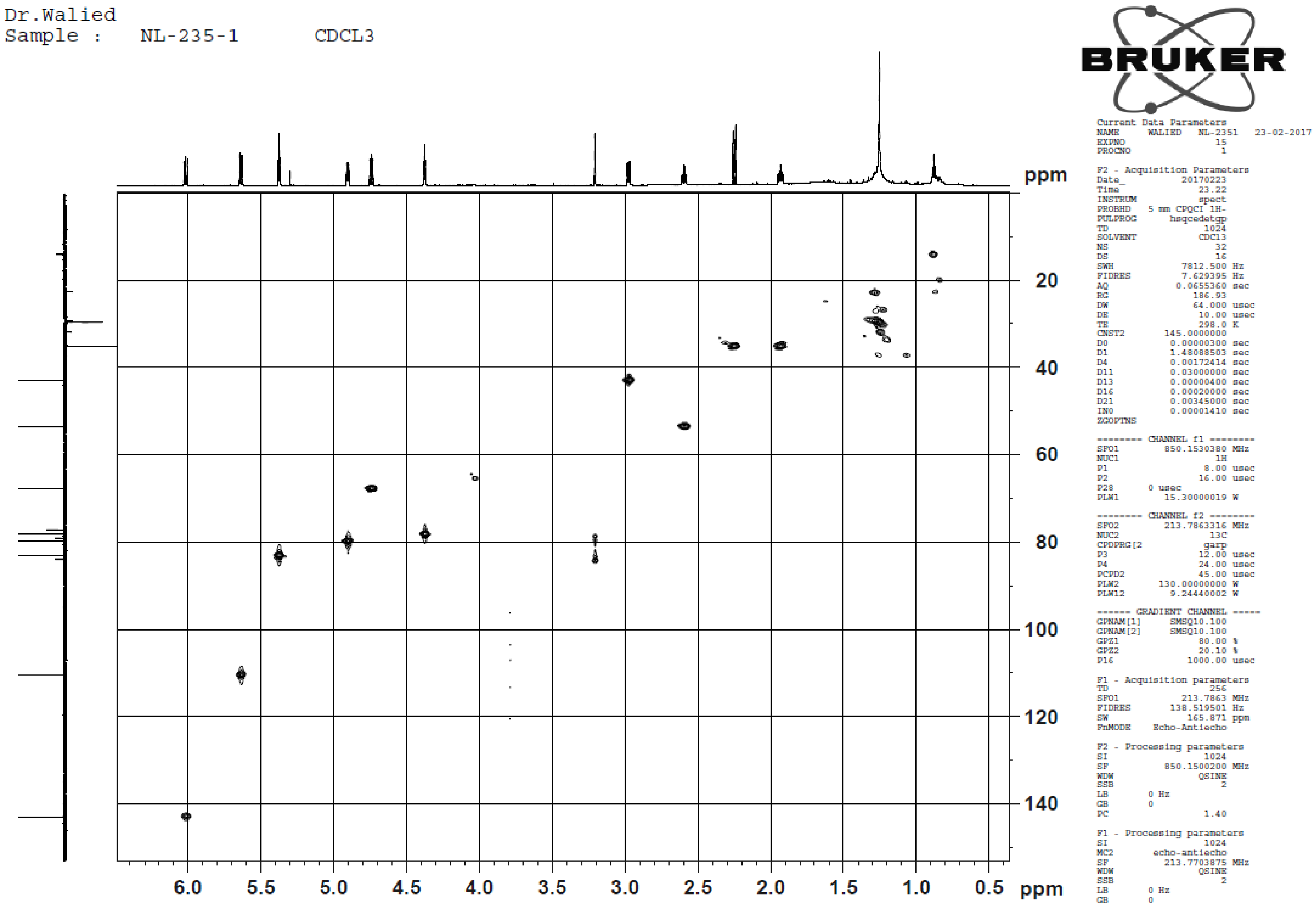

Figure S1g: HSQC NMR of compound 1

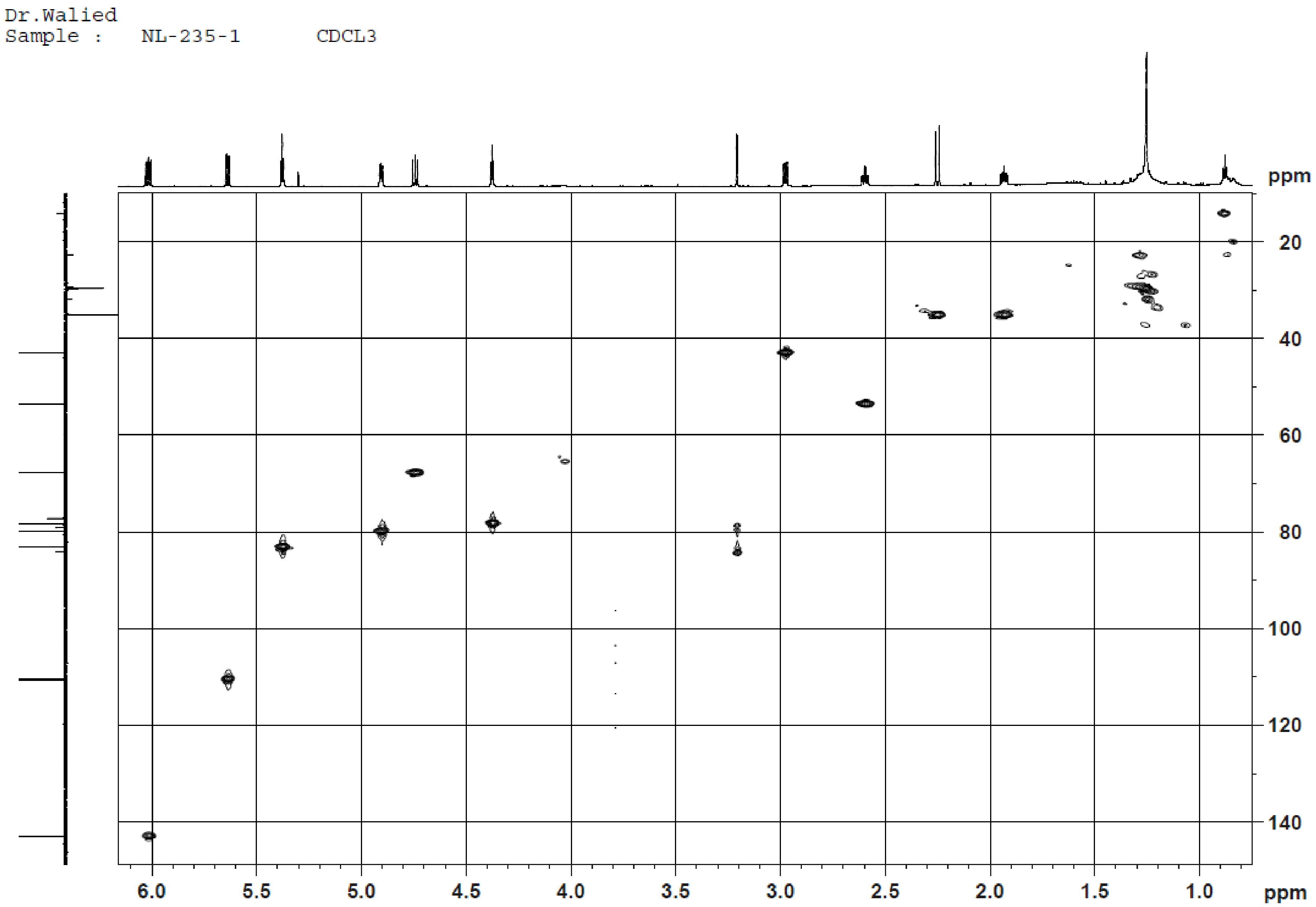

Figure S1h: HSQC NMR of compound 1

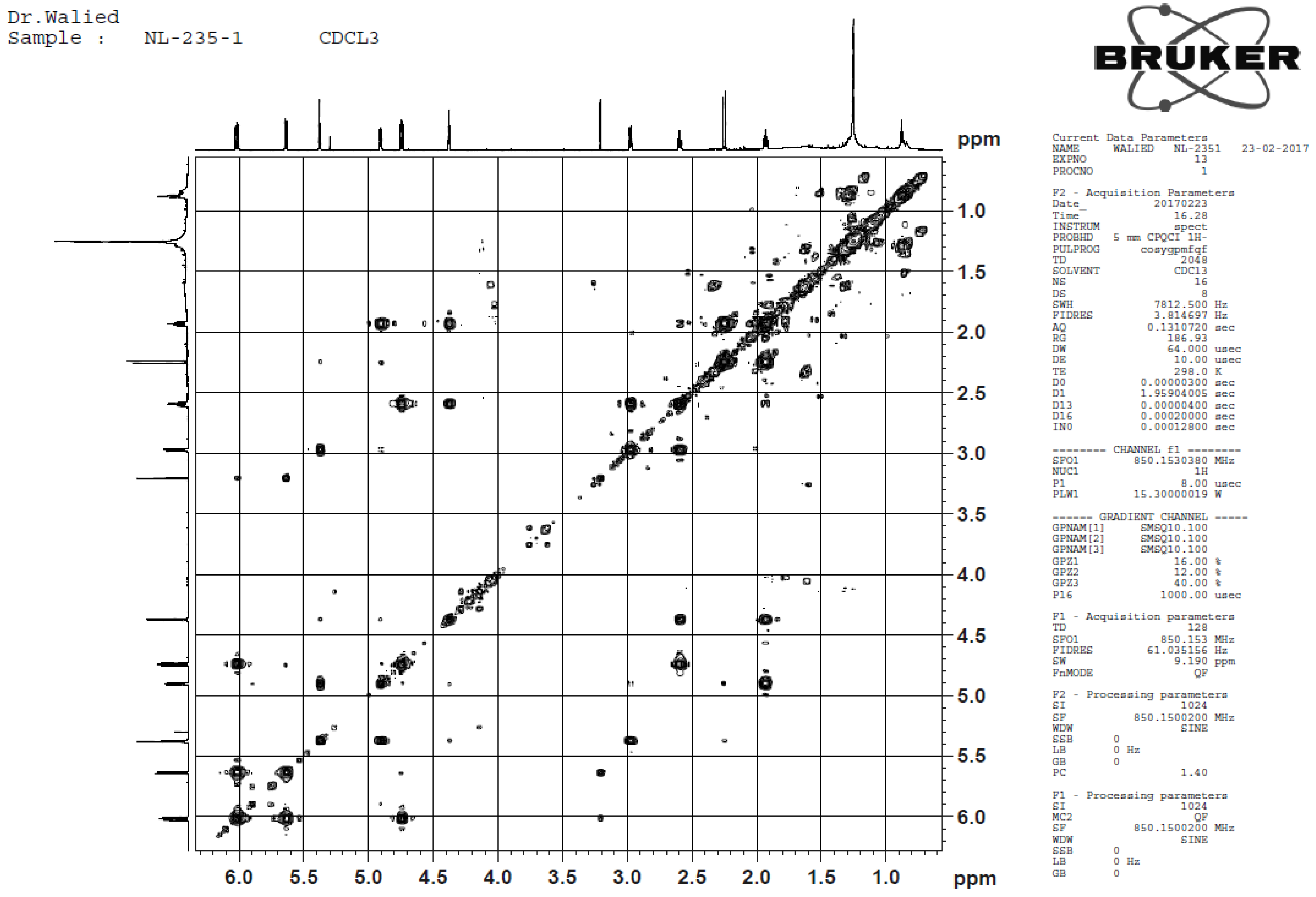

Figure S1i: COSY NMR of compound 1

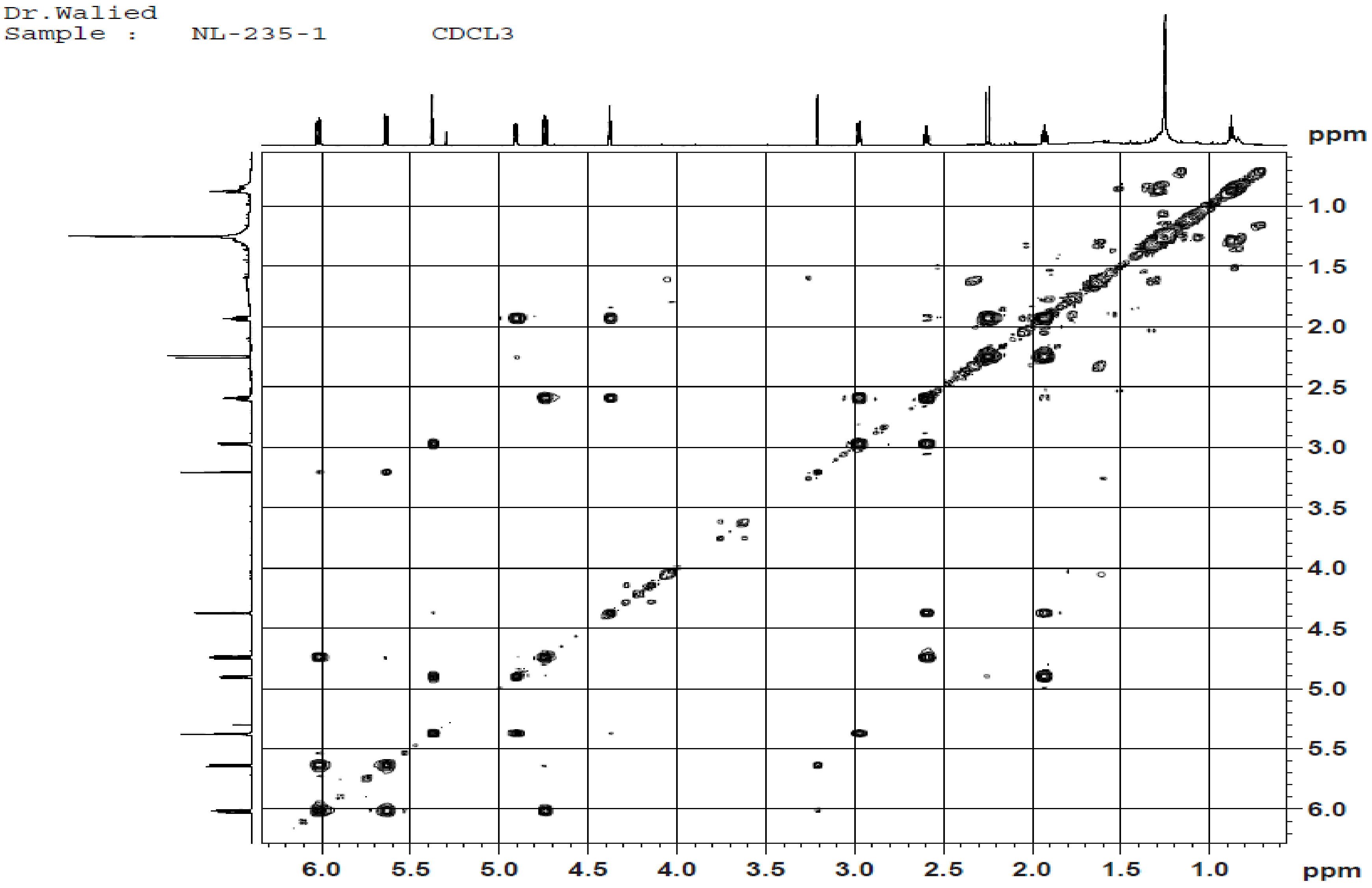

Figure S1j: COSY NMR of compound 1

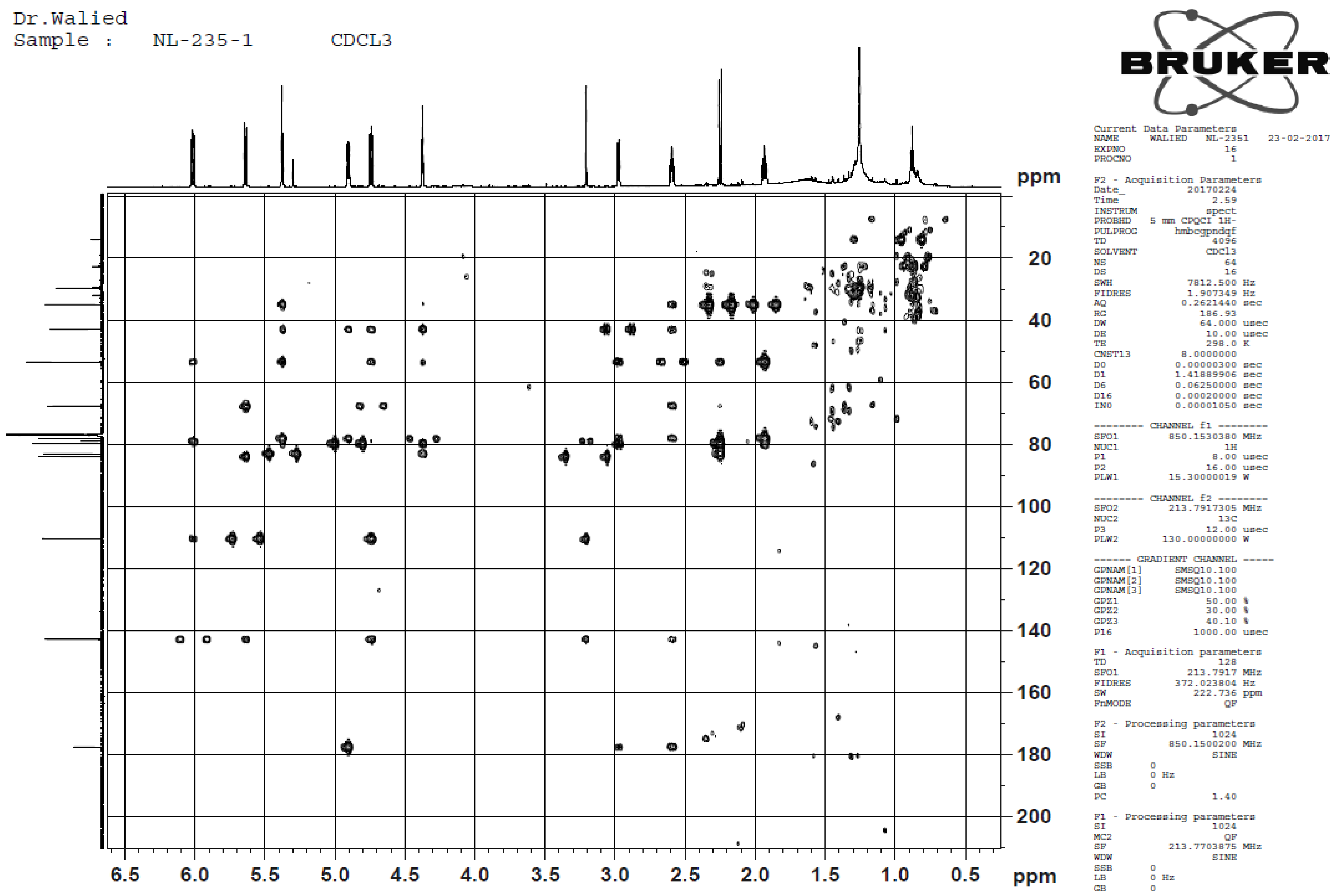

Figure S1k: HMBC NMR of compound 1

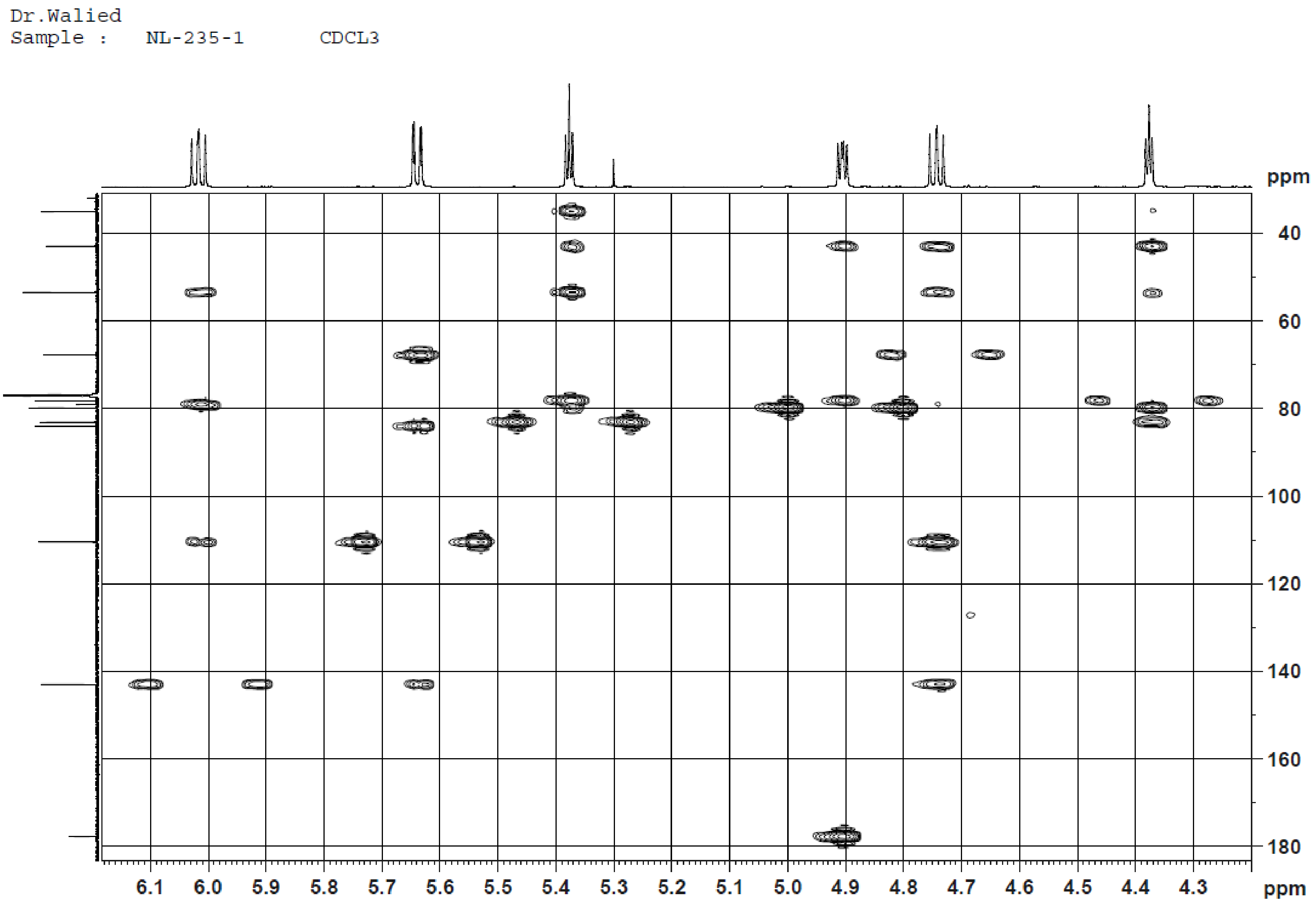

Figure S1l: HMBC NMR of compound 1

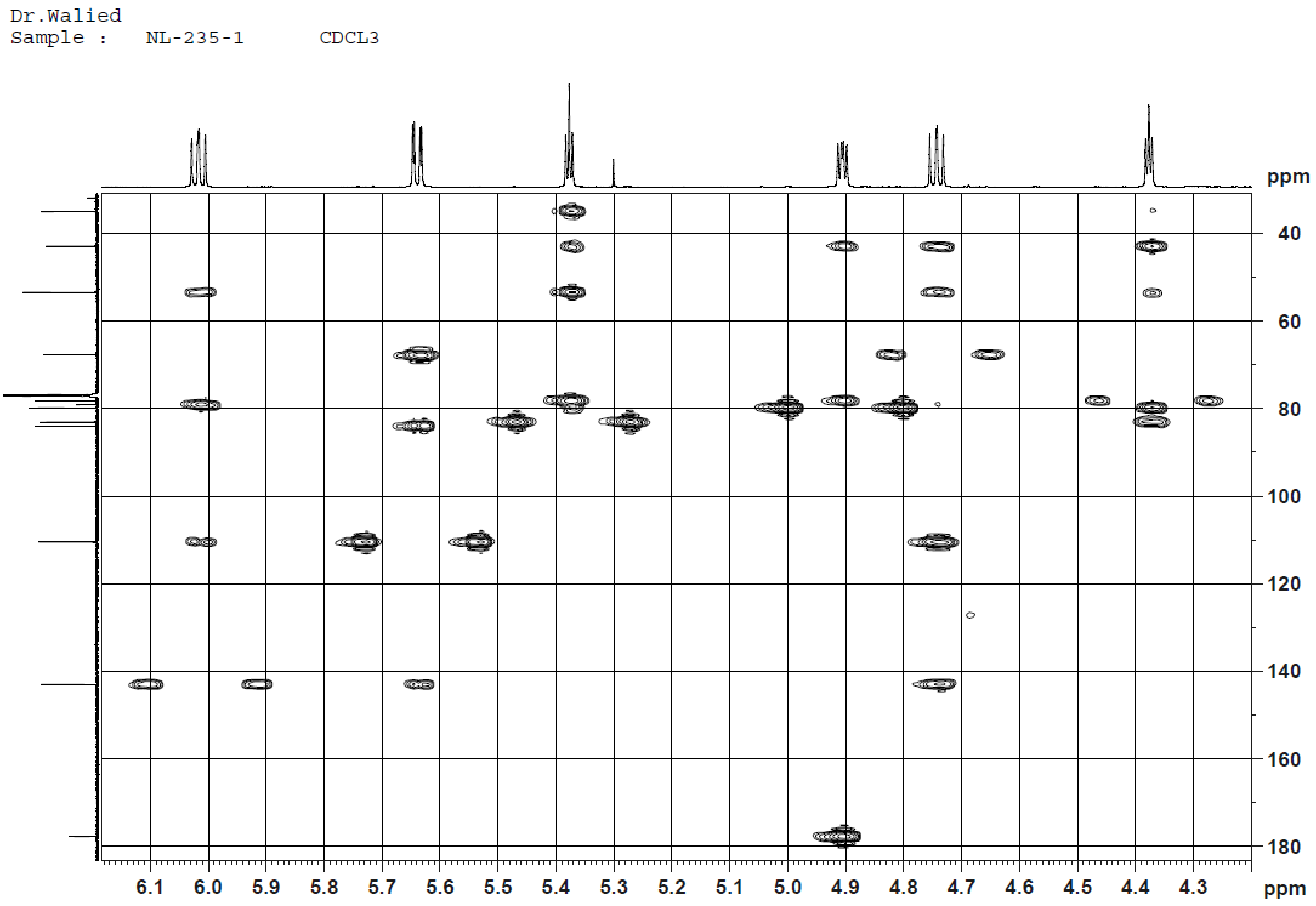

Figure S1m: HMBC NMR of compound 1

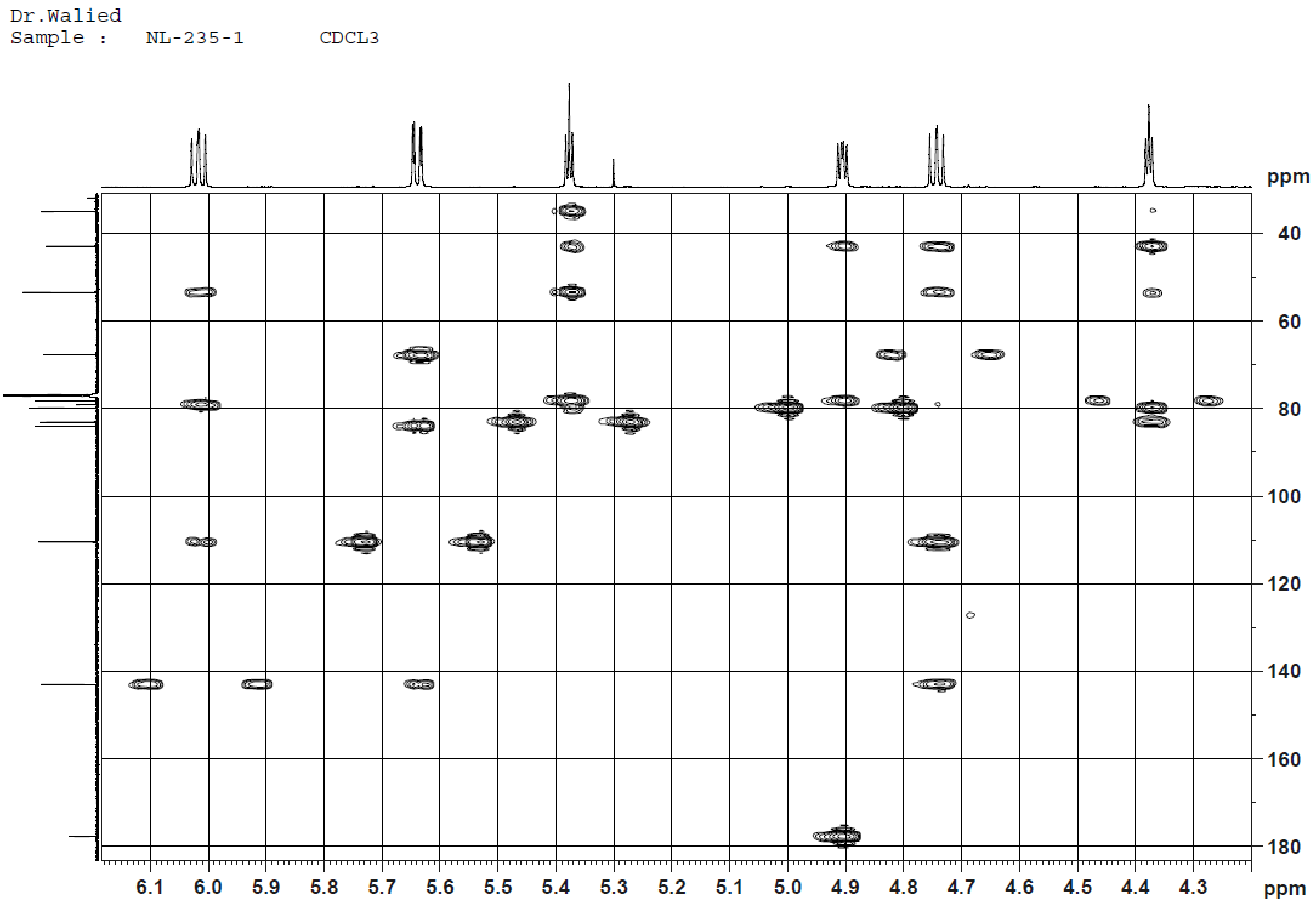

Figure S1n: HMBC NMR of compound 1

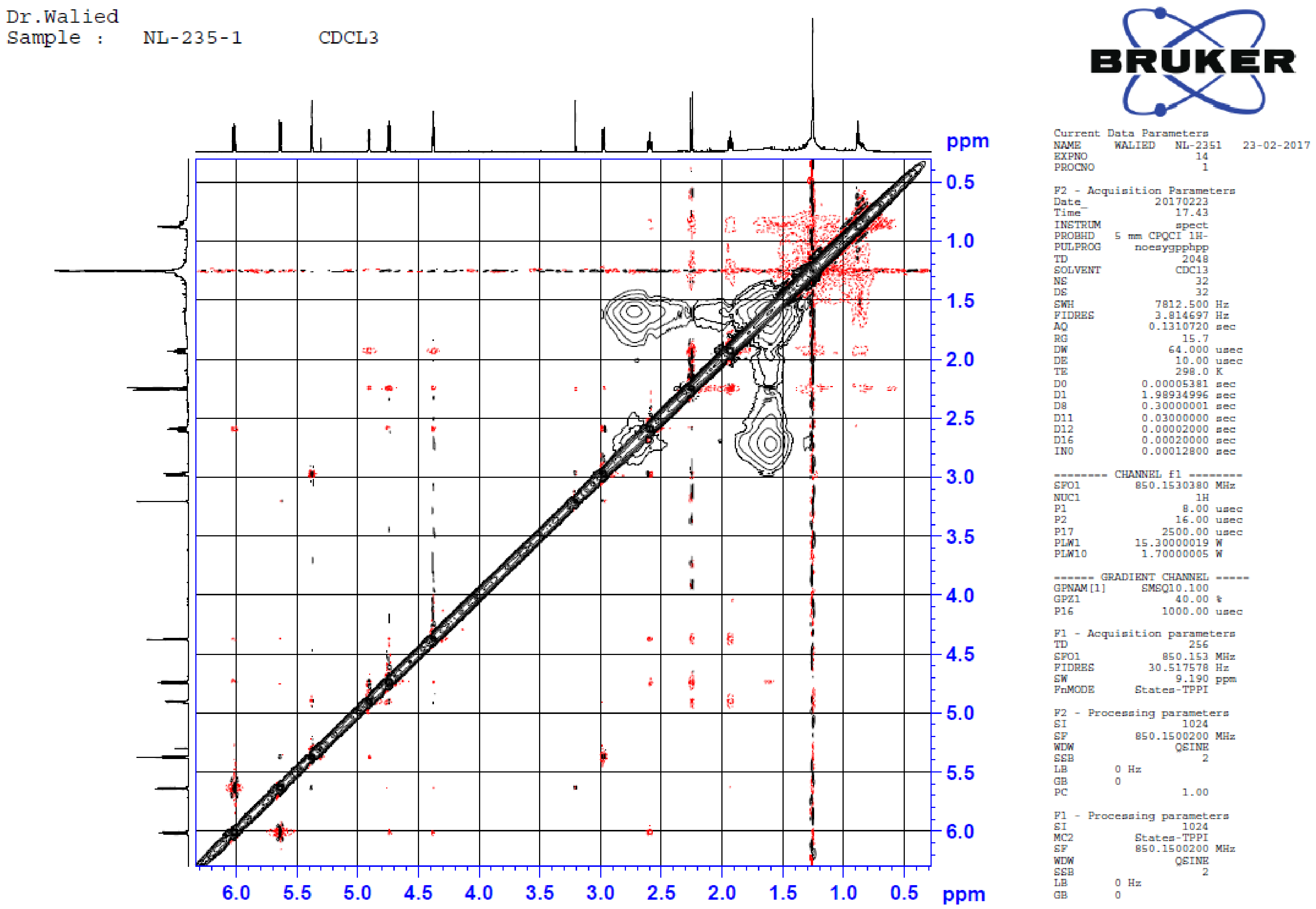

Figure S1o: NOESY NMR of compound 1

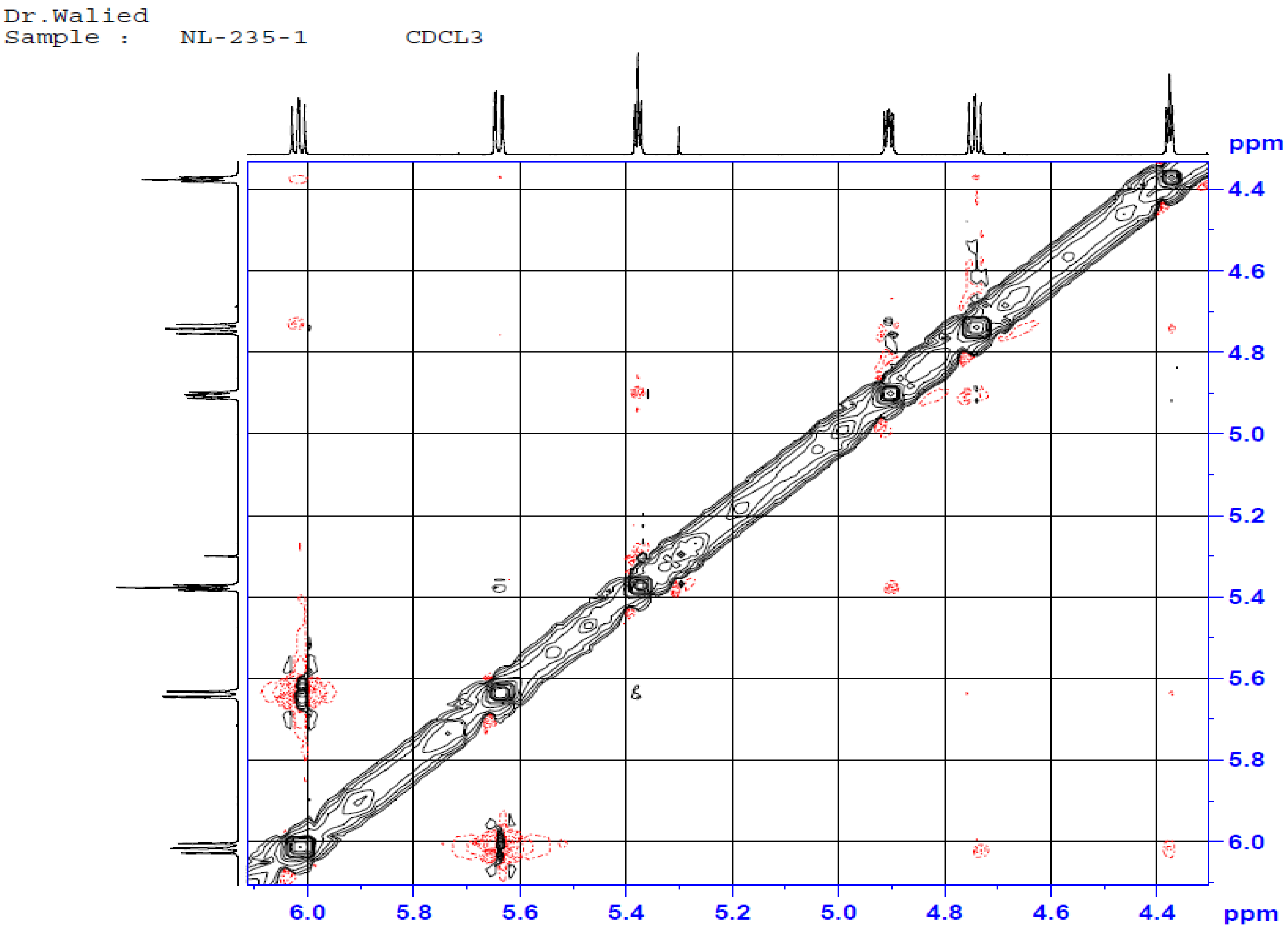

Figure S1p: NOESY NMR of compound 1

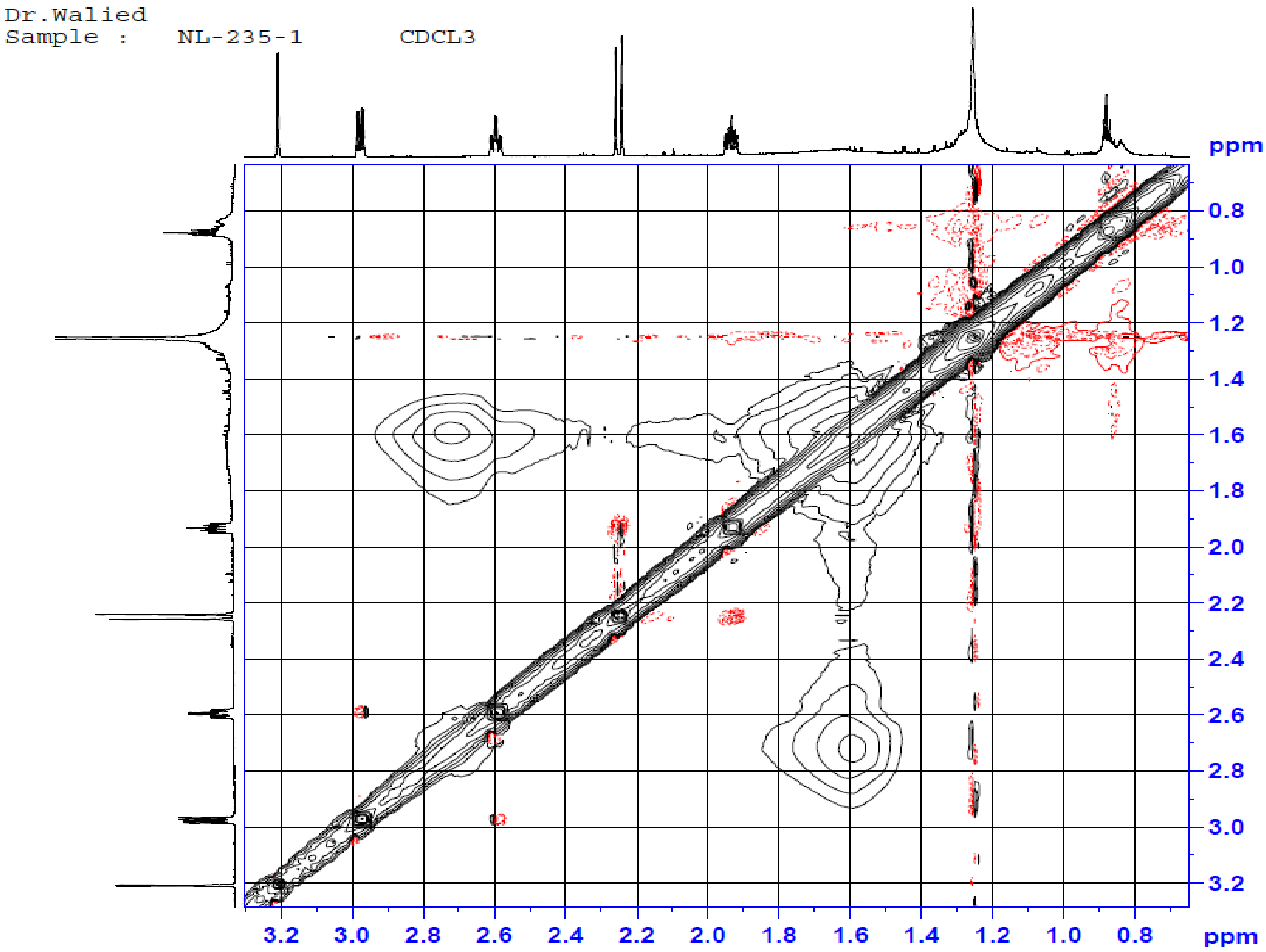

Figure S1q: NOESY NMR of compound 1

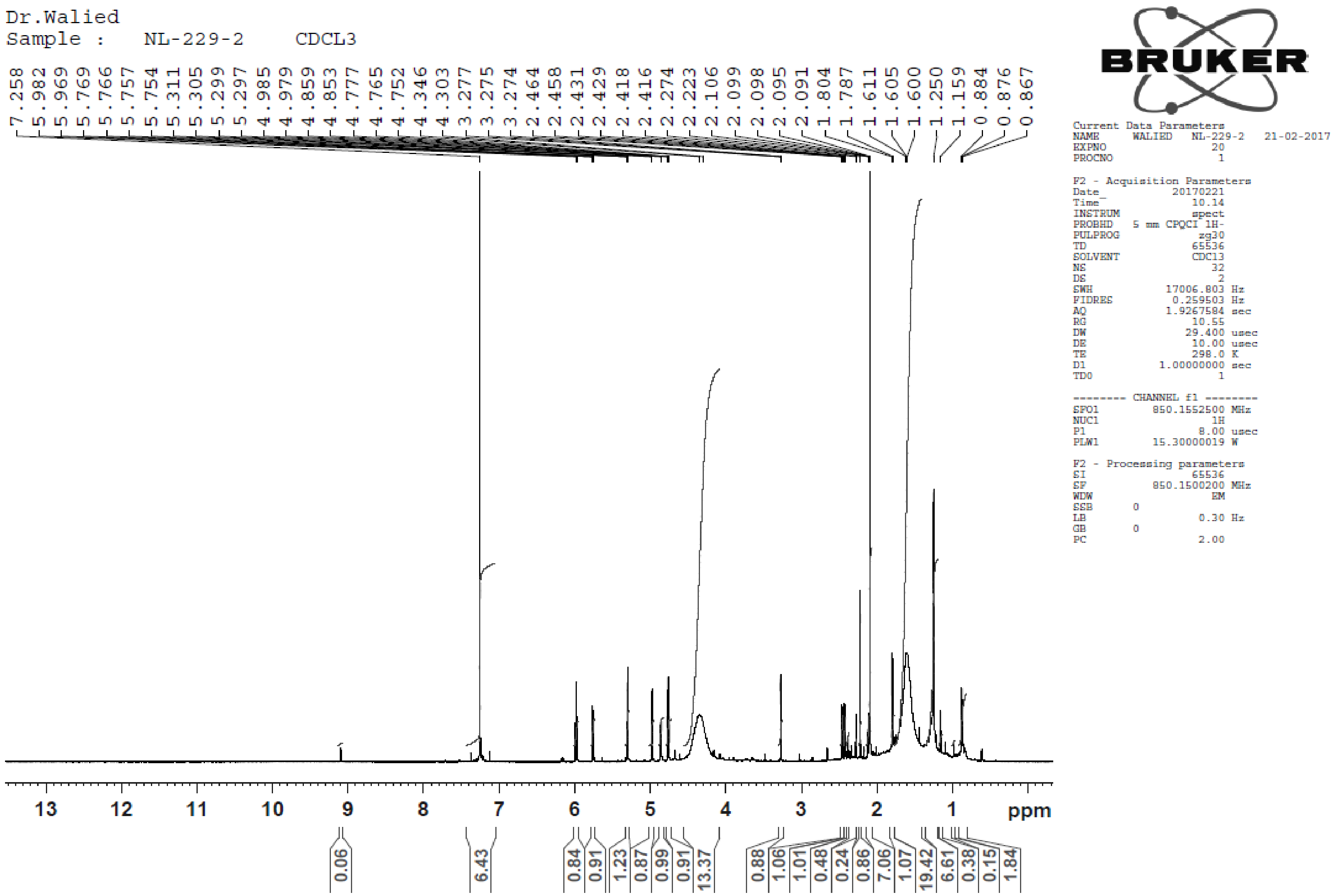

Figure S2a: ^{1}H NMR of compound 2

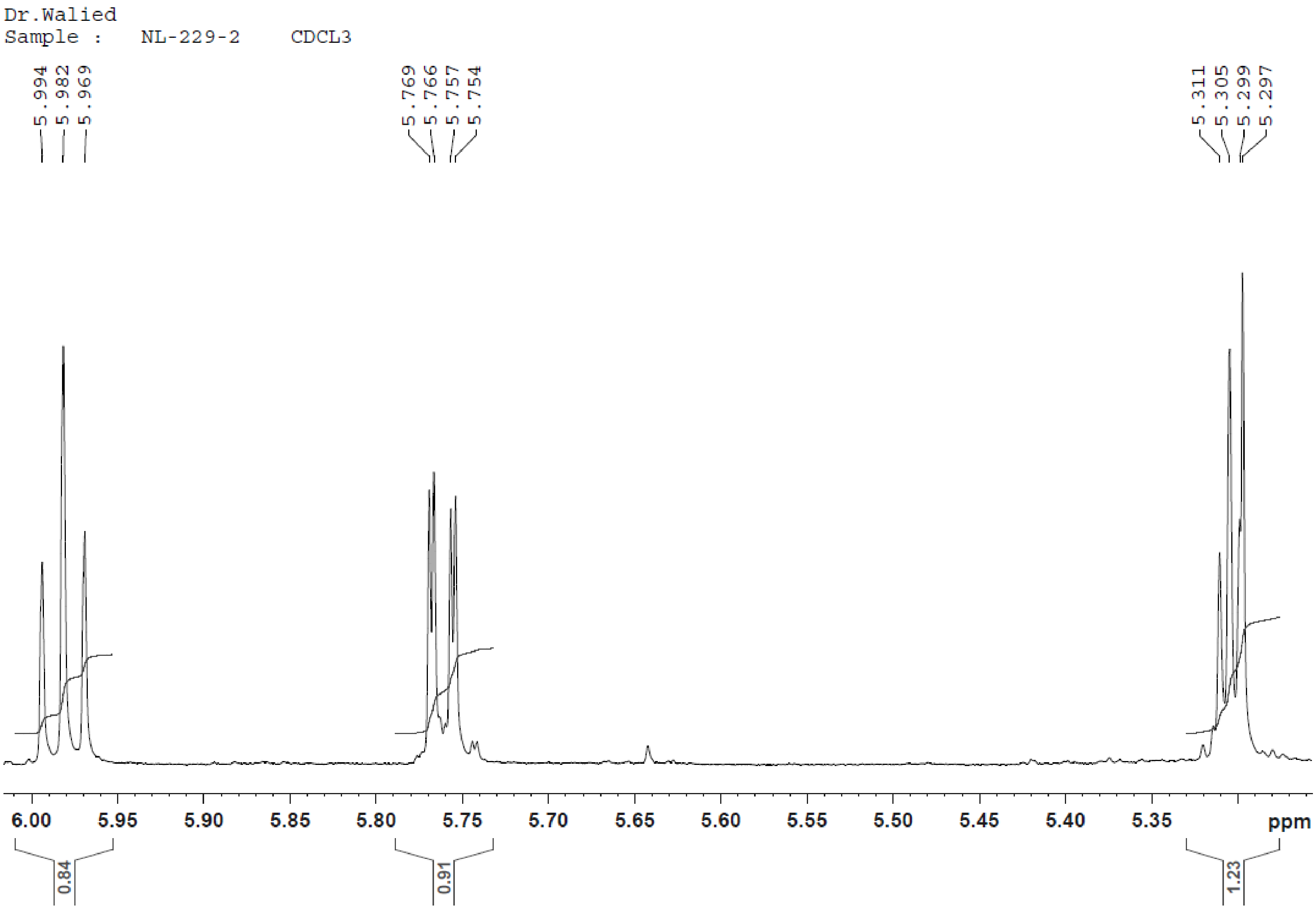

Figure S2a: ^{1}H NMR of compound 2

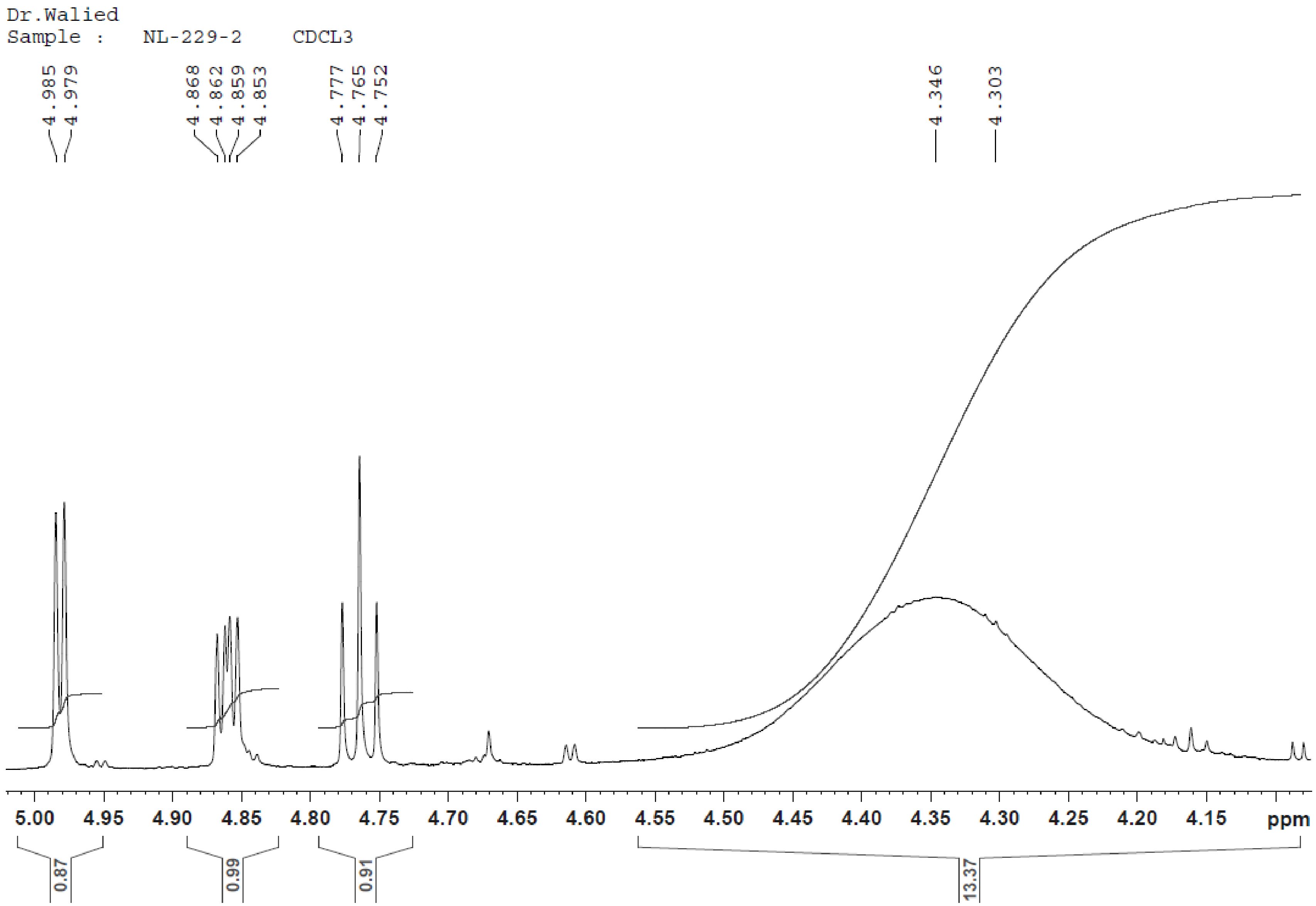

Figure S2b: ^{1}H NMR of compound 2

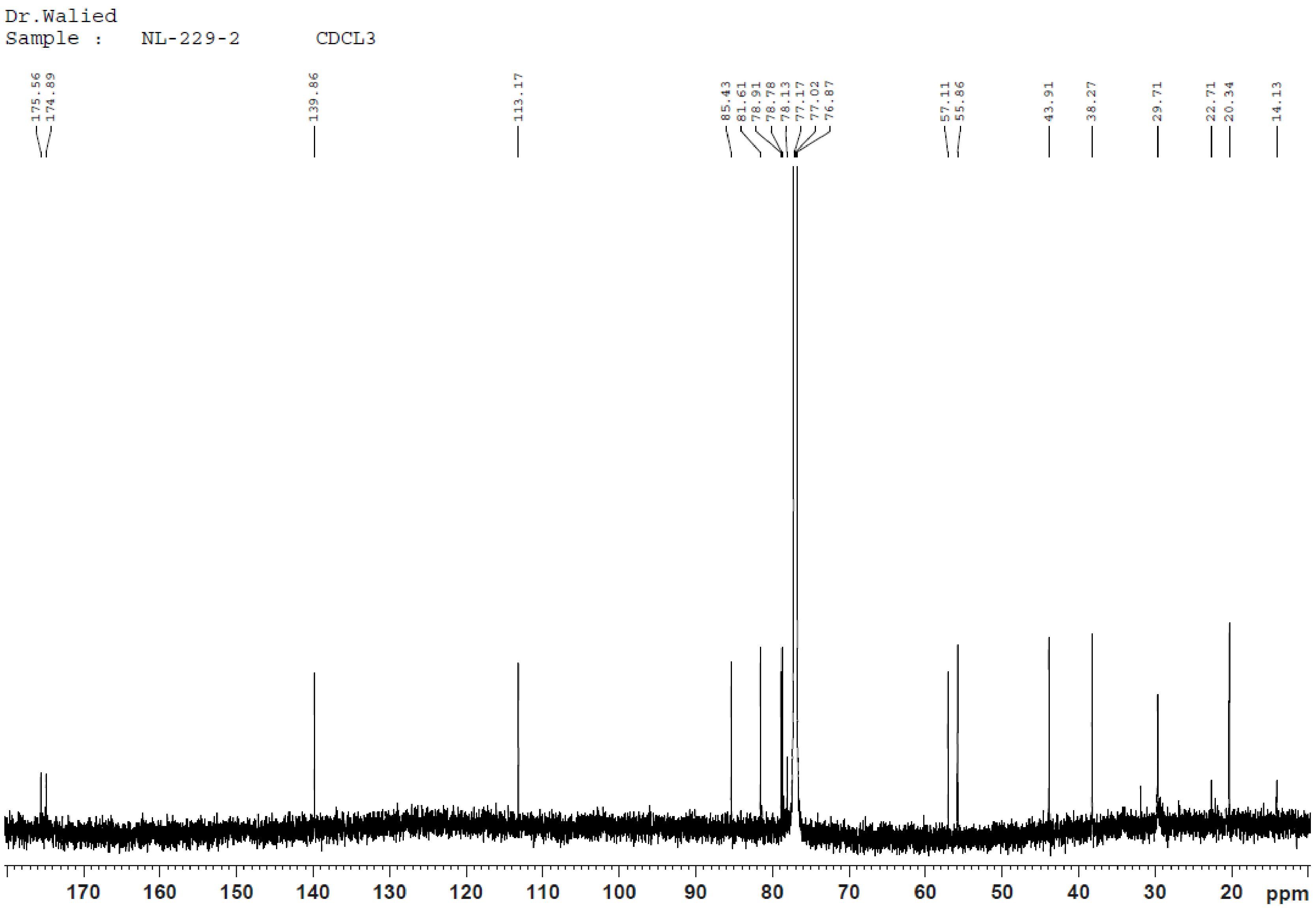

Figure S2c: ^{13}C NMR of compound 2

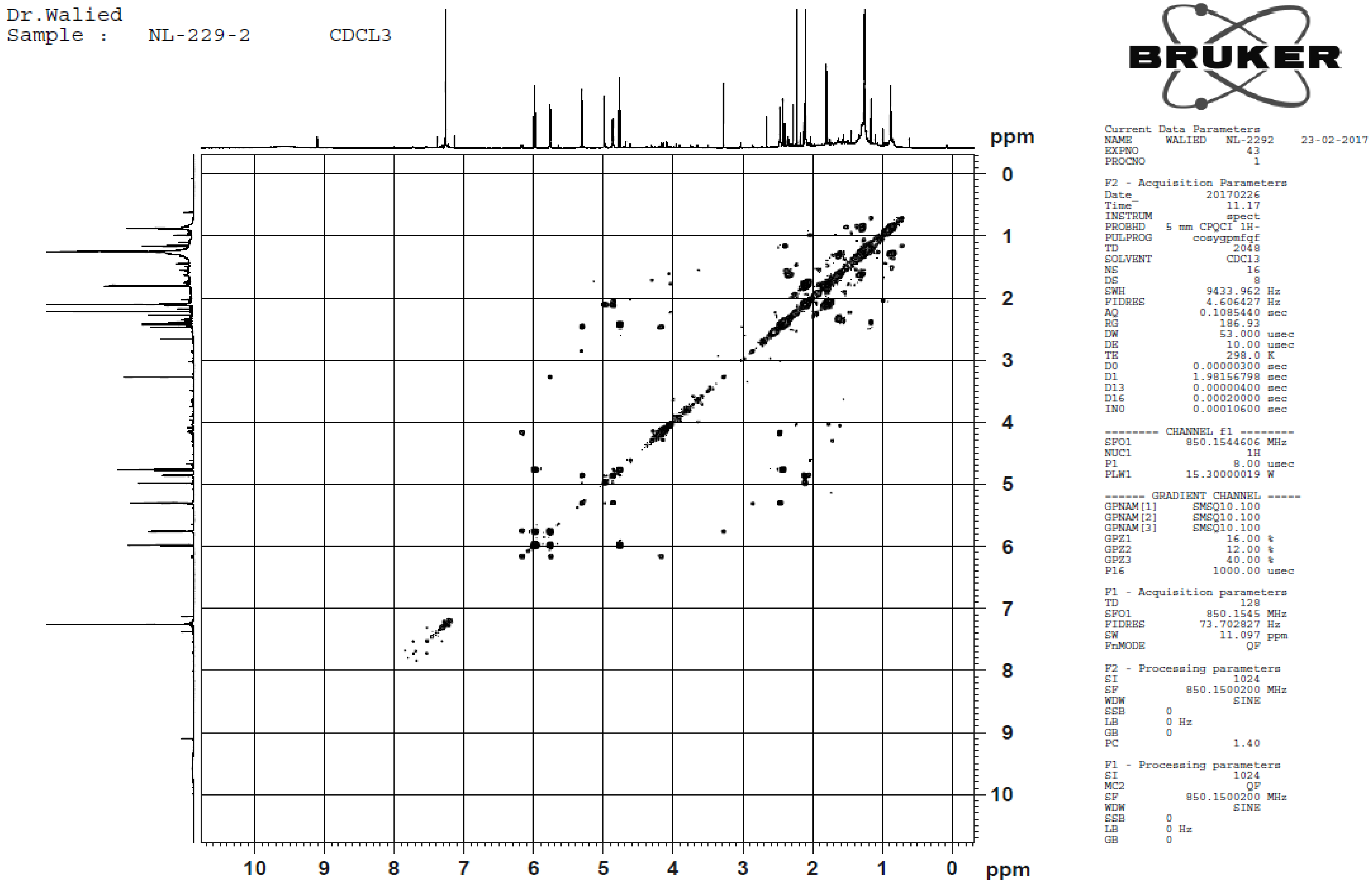

Figure S2d: COSY NMR of compound 2

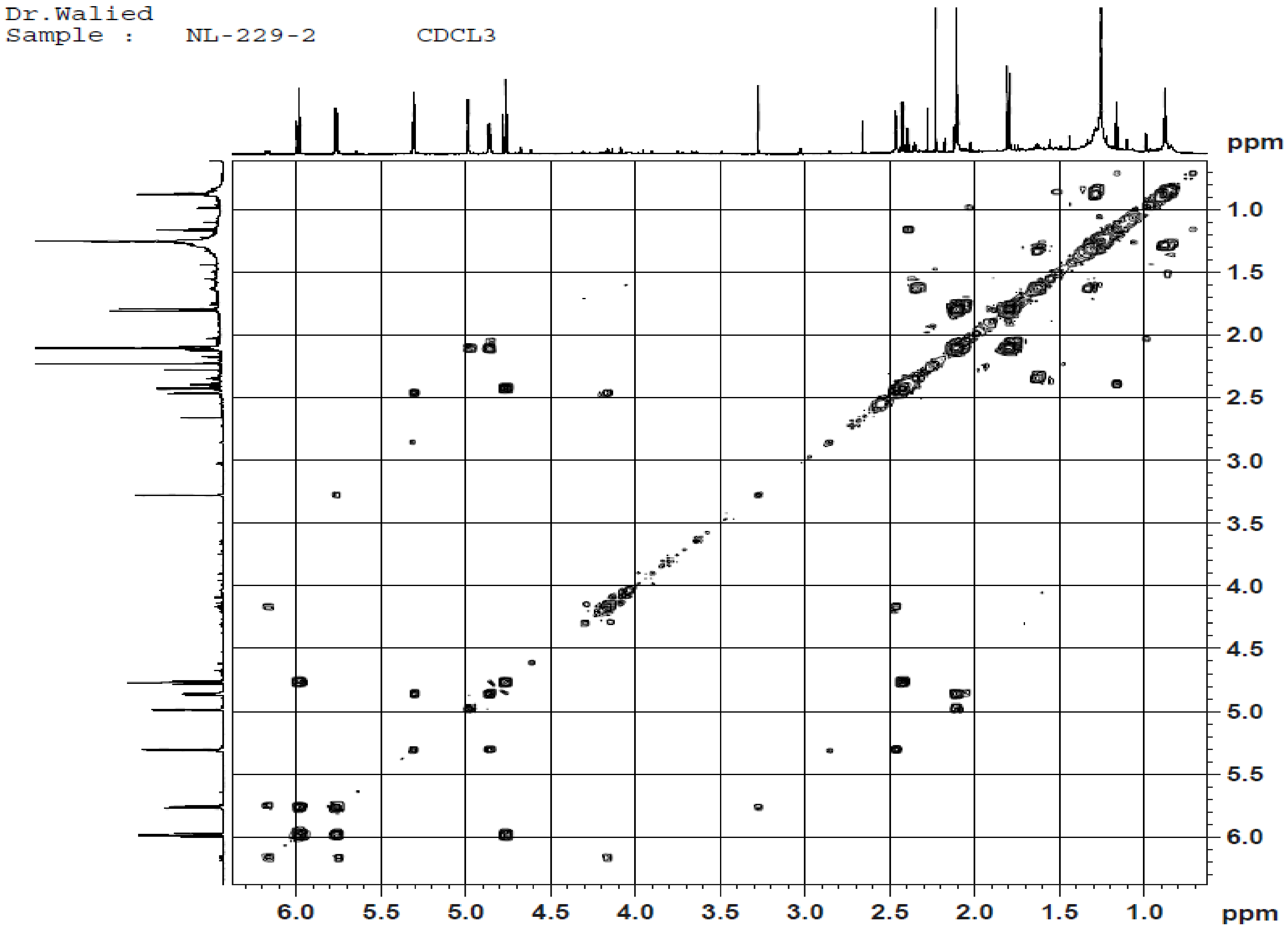

Figure S2e: COSY NMR of compound 2

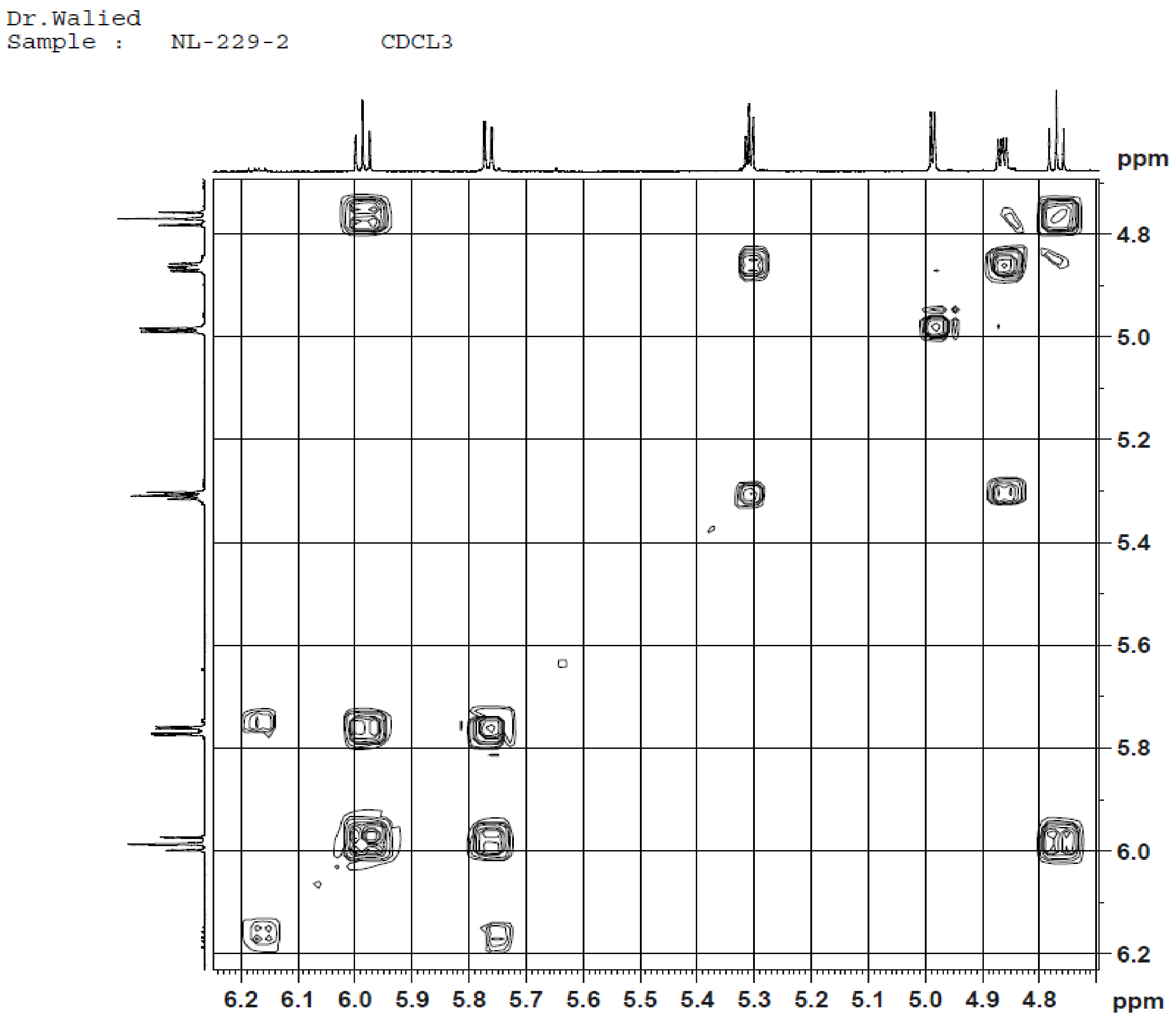

Figure S2f: COSY NMR of compound 2

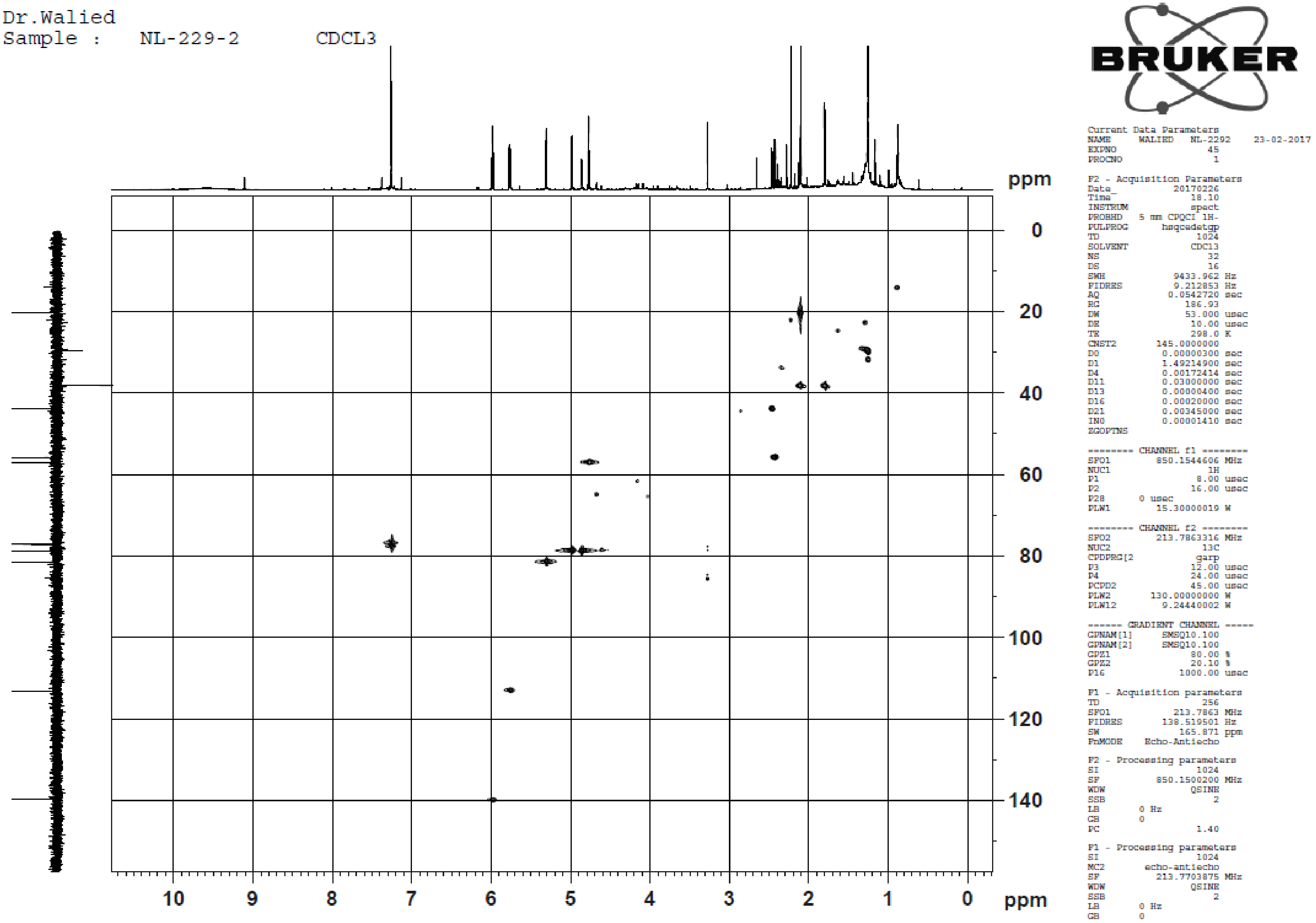

Figure S2g: HSQC NMR of compound 2

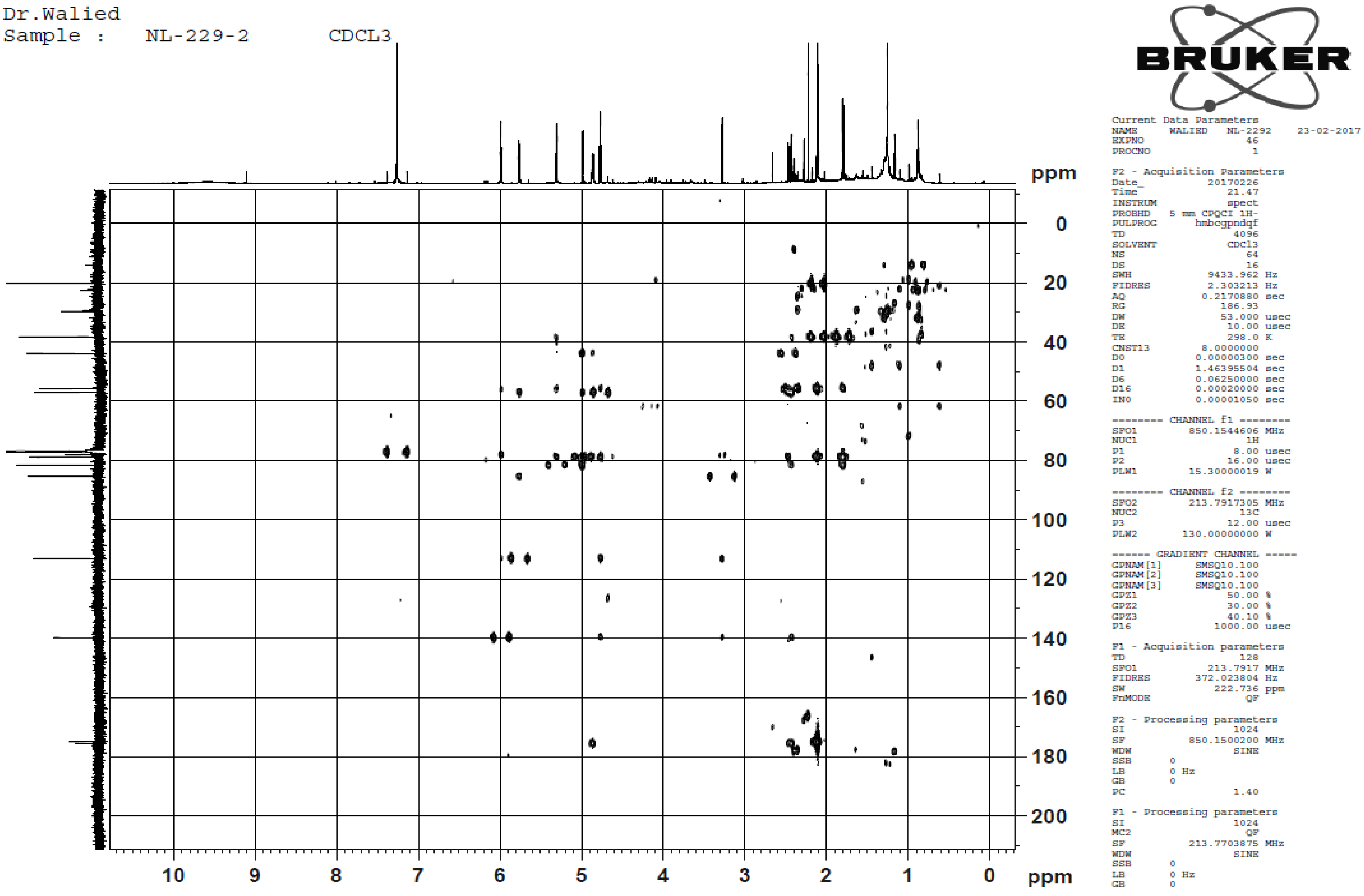

Figure S2h: HMBC NMR of compound 2

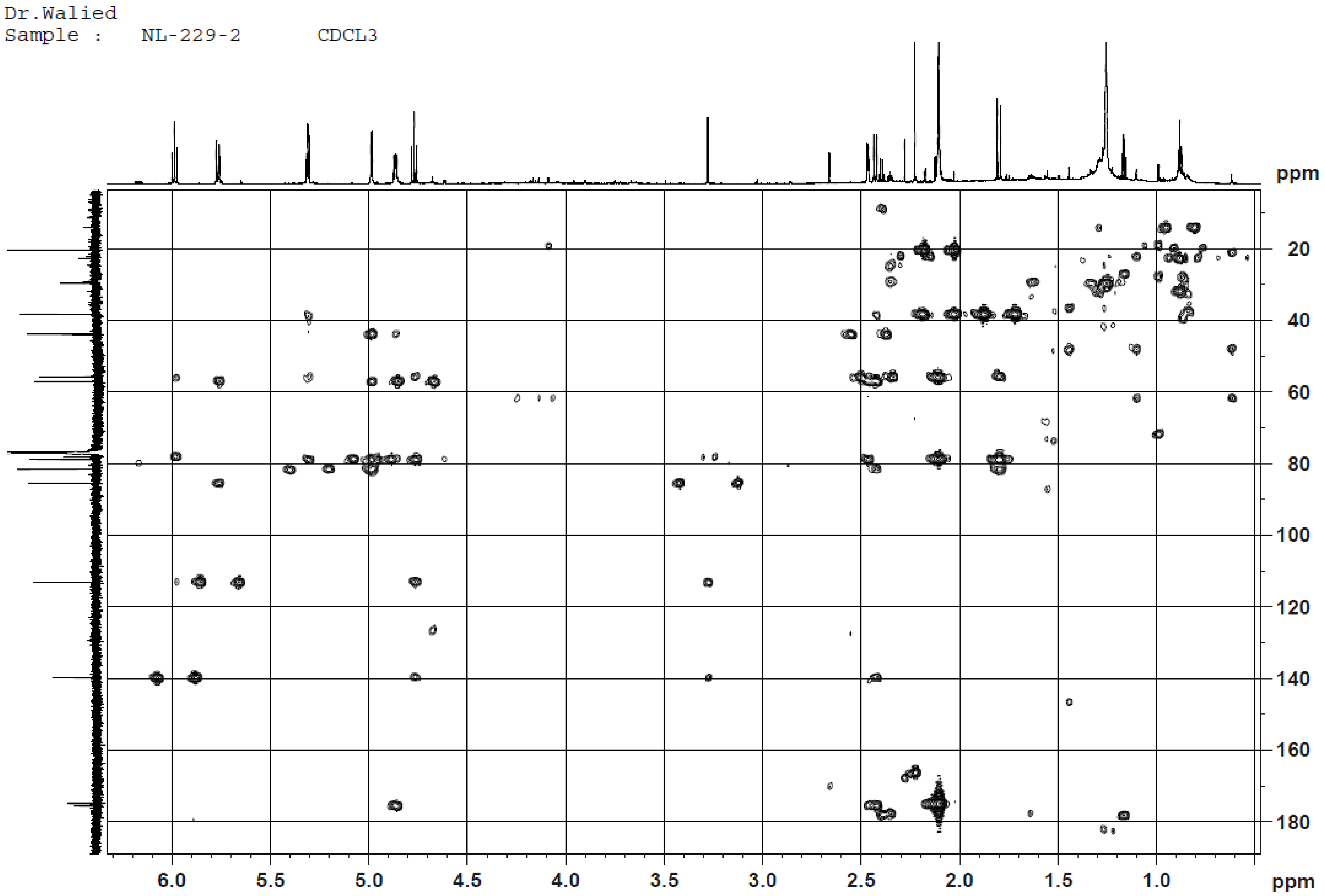

Figure S2i: HMBC NMR of compound 2

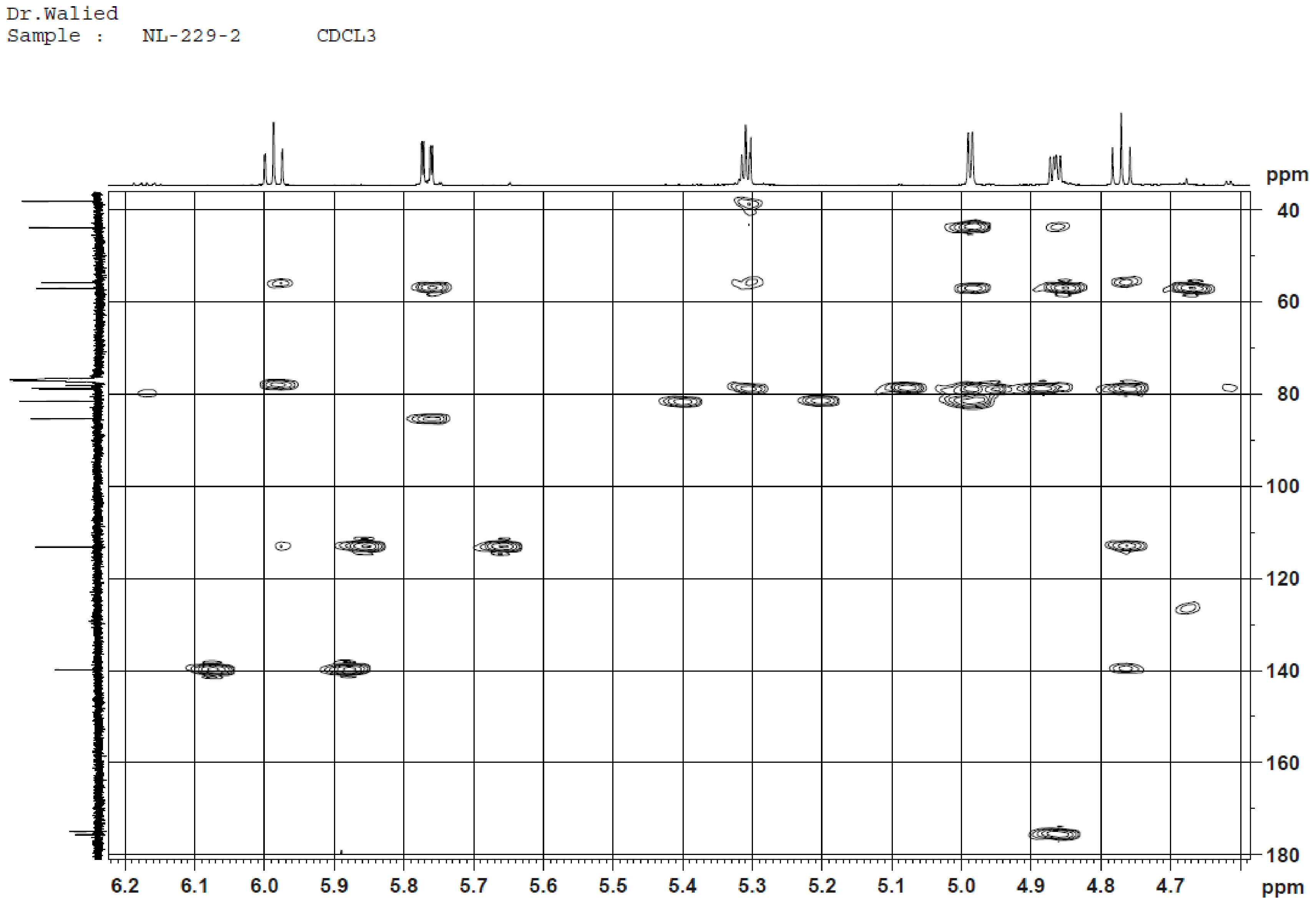

Figure S2j: HMBC NMR of compound 2

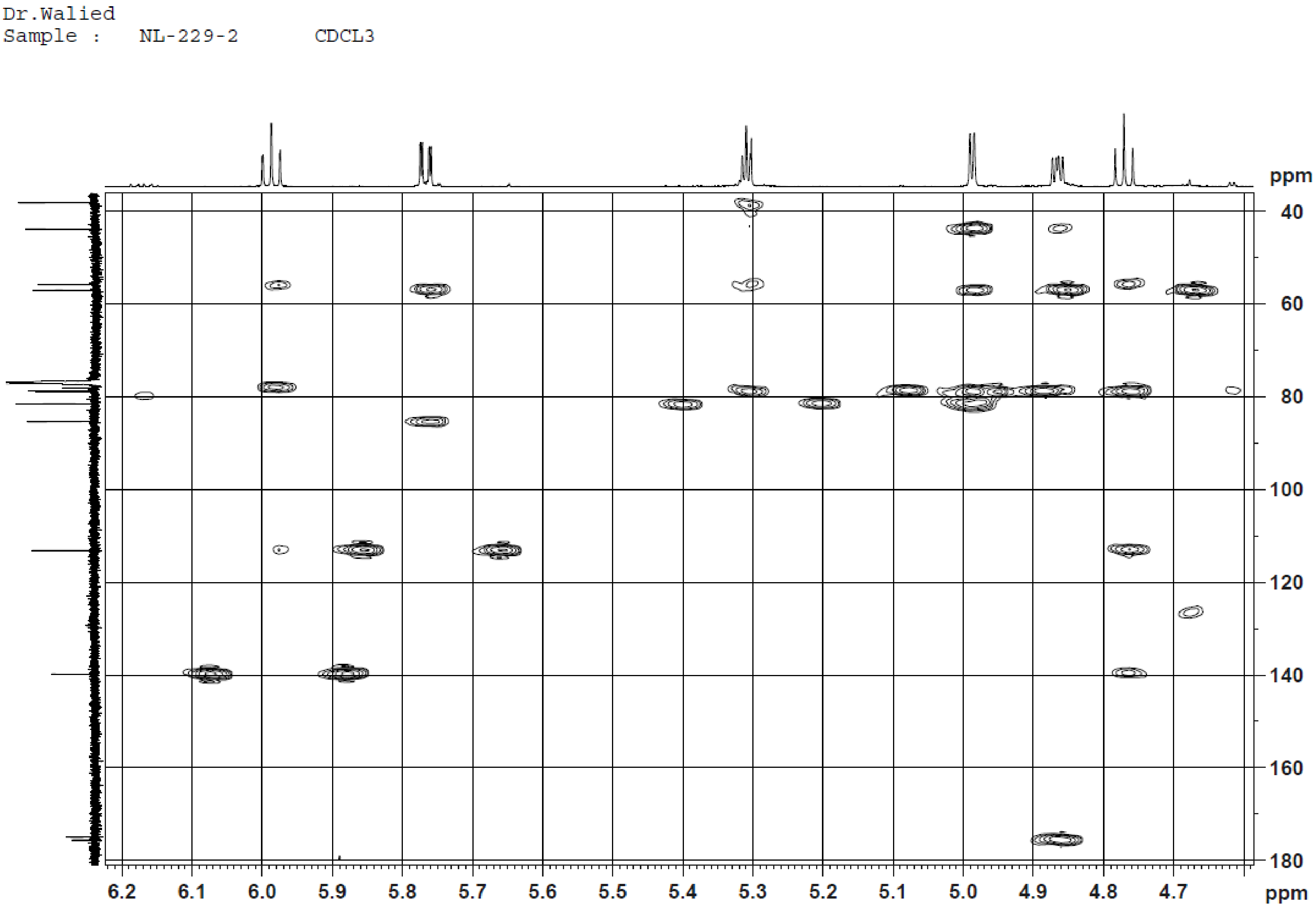

Figure S2k: HMBC NMR of compound 2

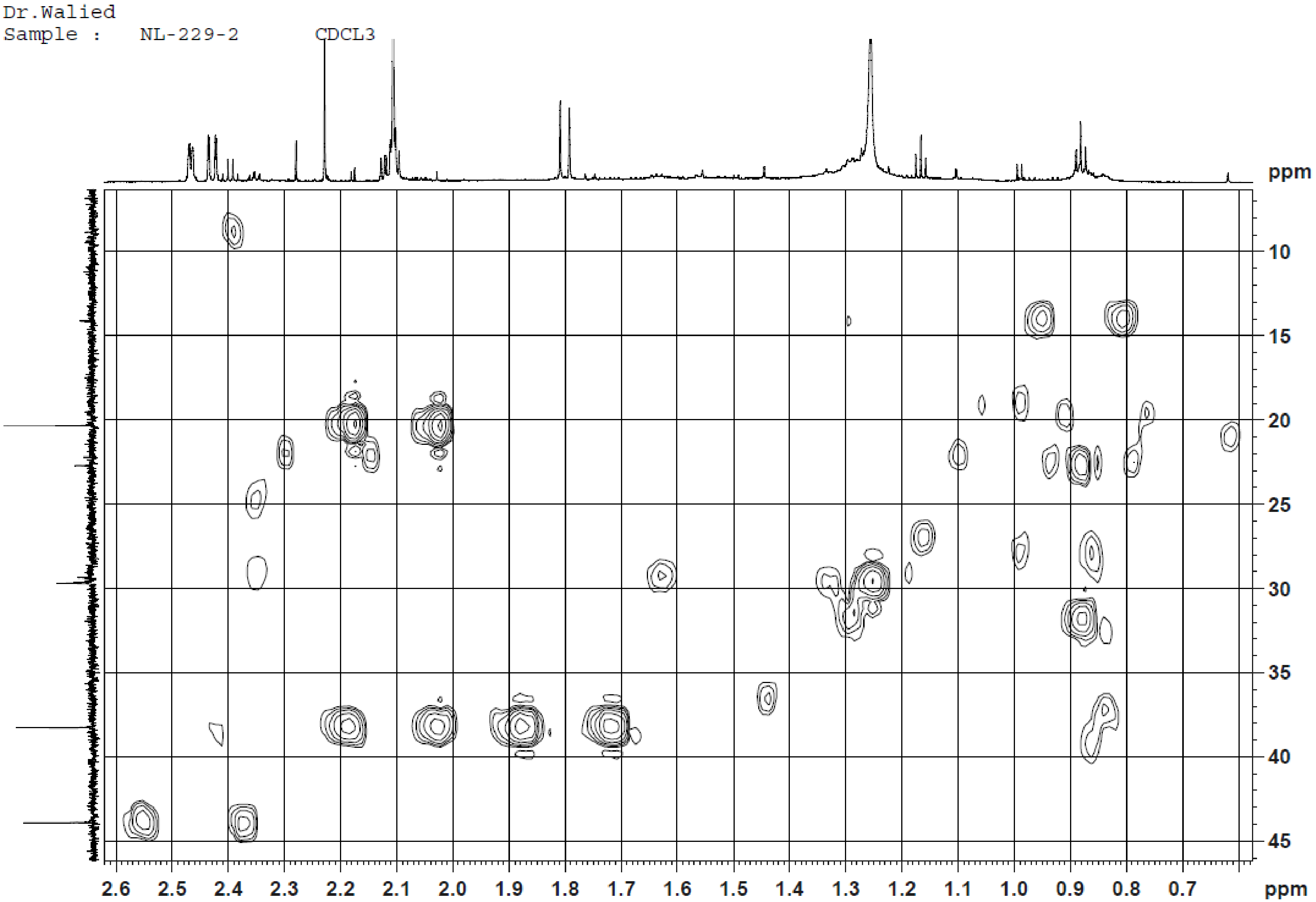

Figure S2l: HMBC NMR of compound 2

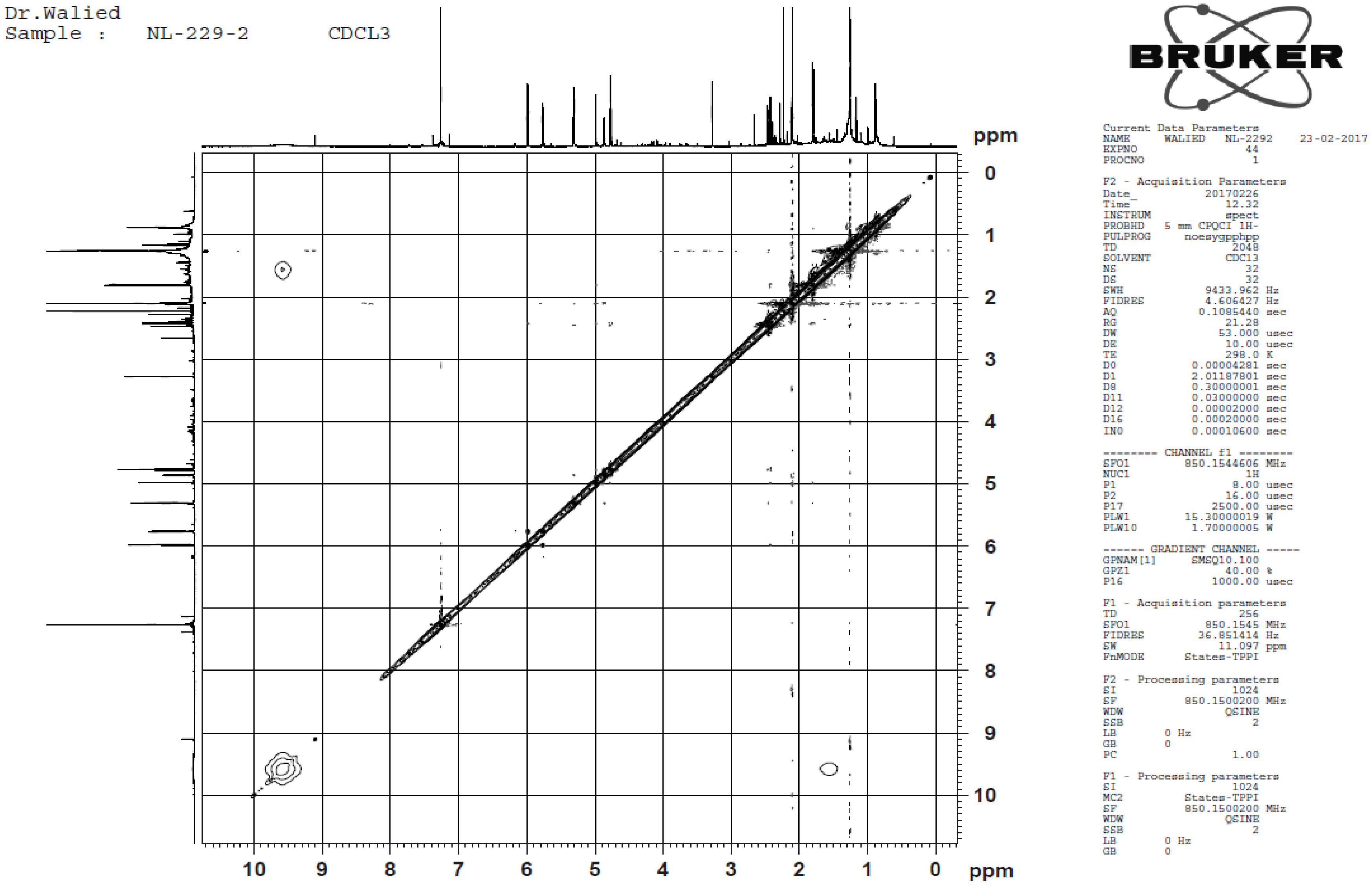

Figure S2l: NOSY NMR of compound 2

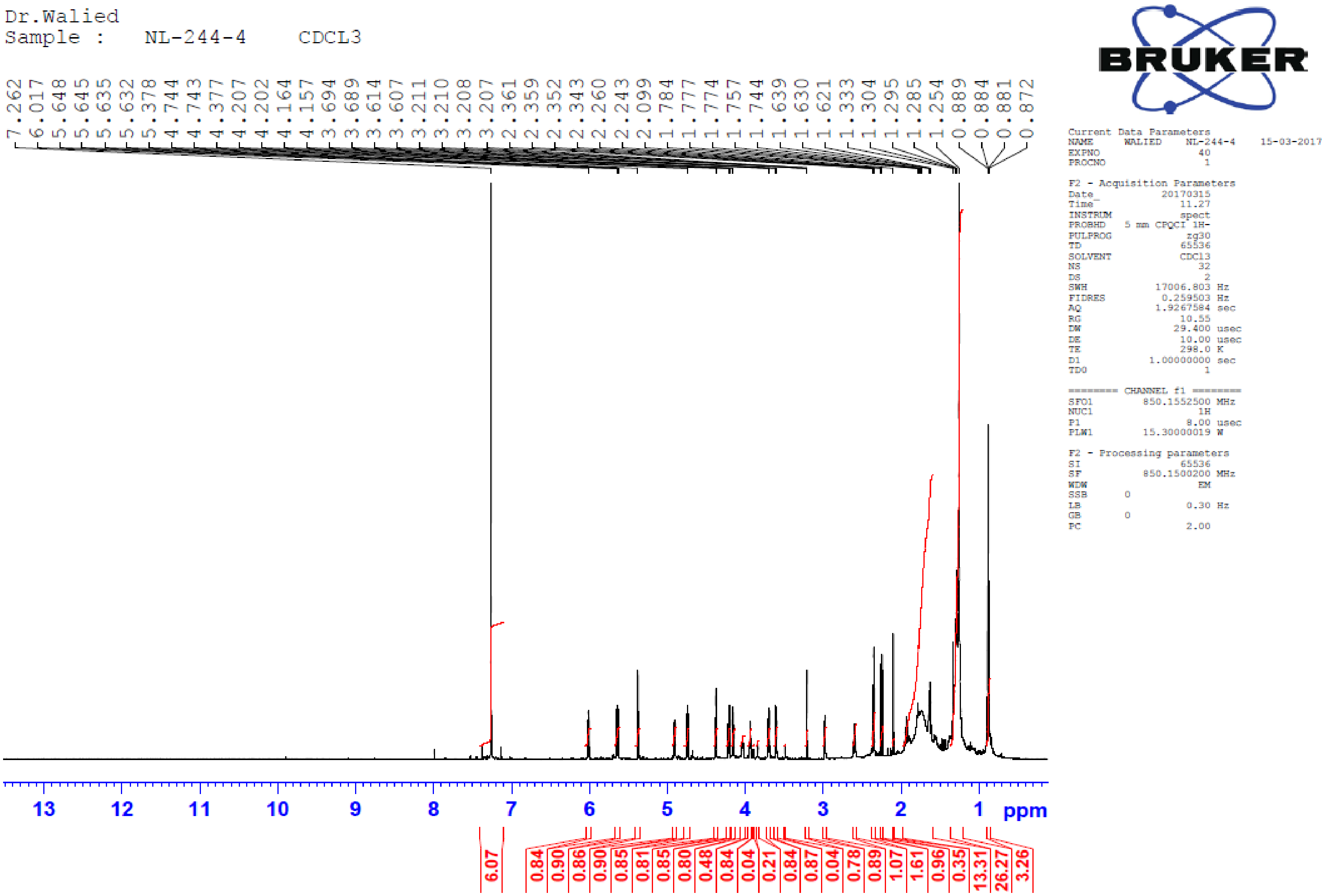

Figure S3a: ^{1}H NMR of compound 3

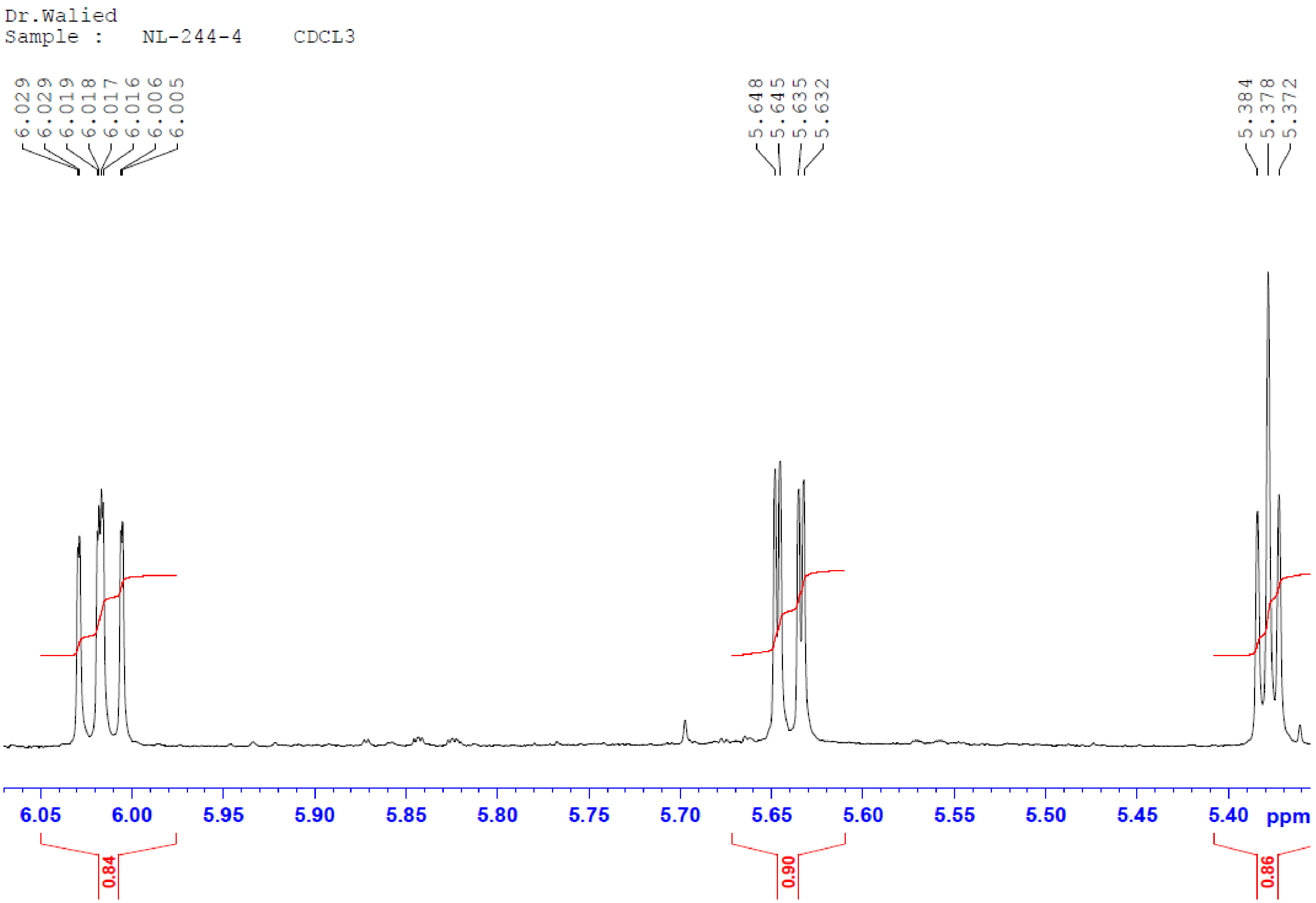

Figure S3b: ^{1}H NMR of compound 3

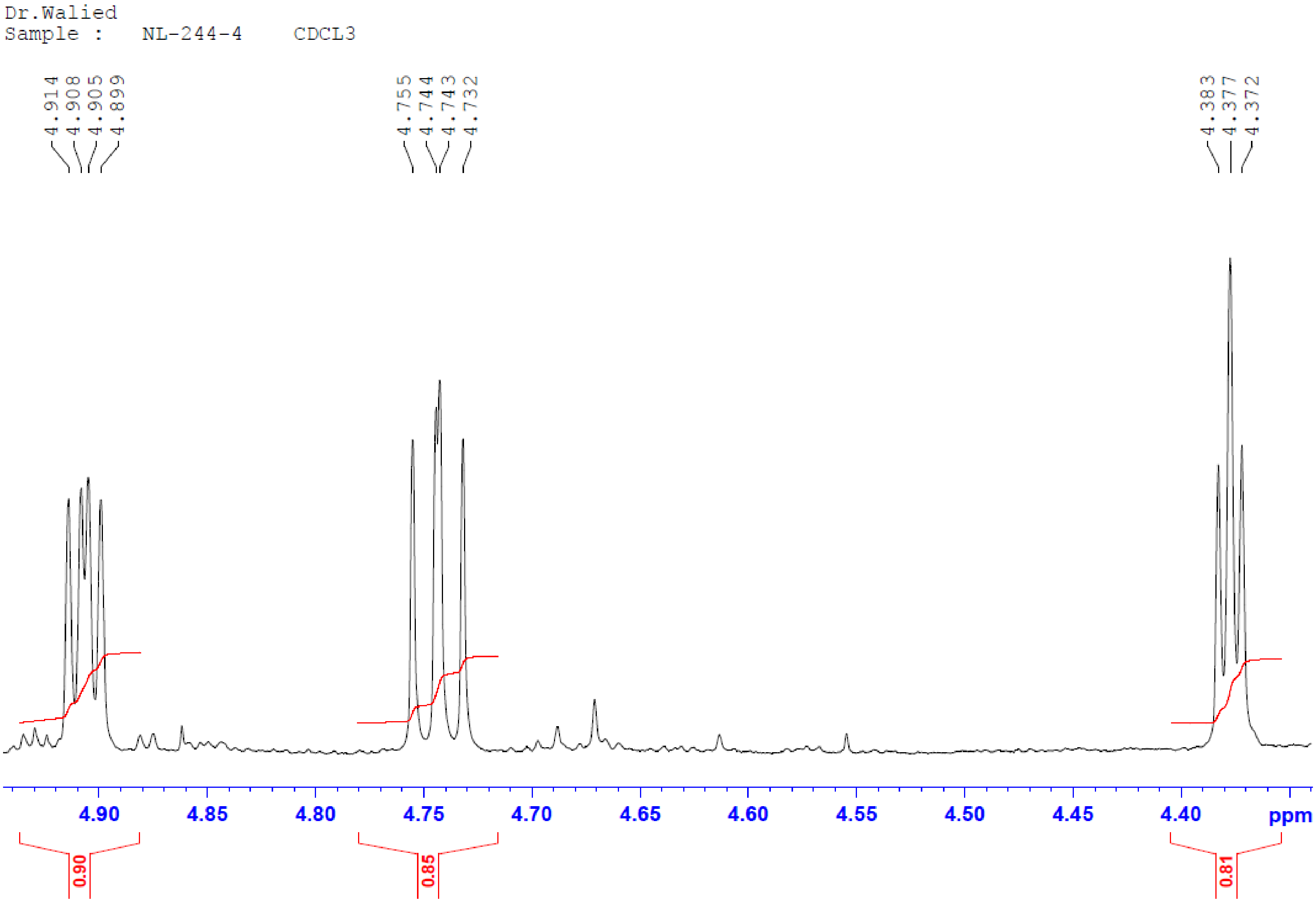

Figure S3c: ^{1}H NMR of compound 3

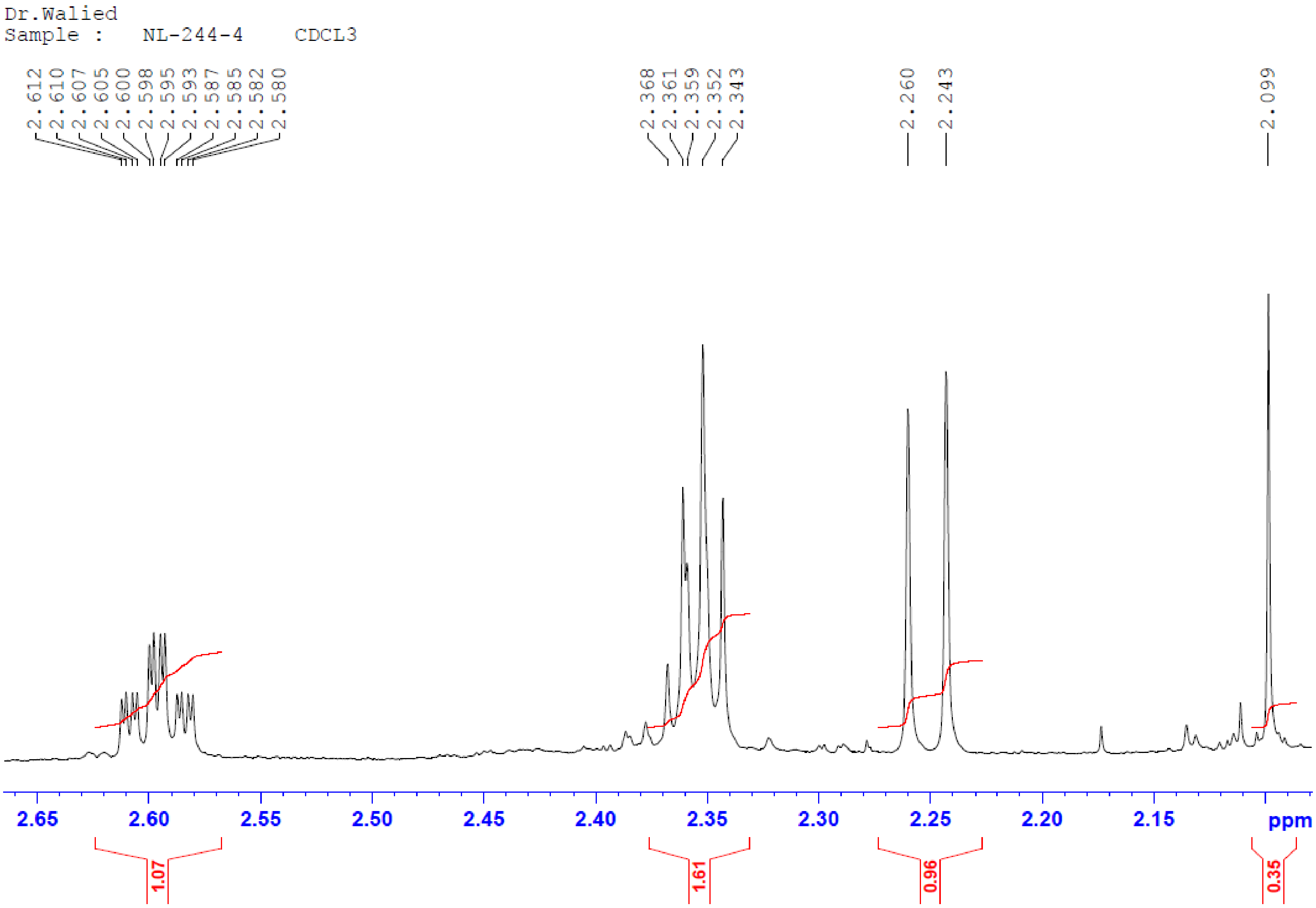

Figure S3d: ^{1}H NMR of compound 3

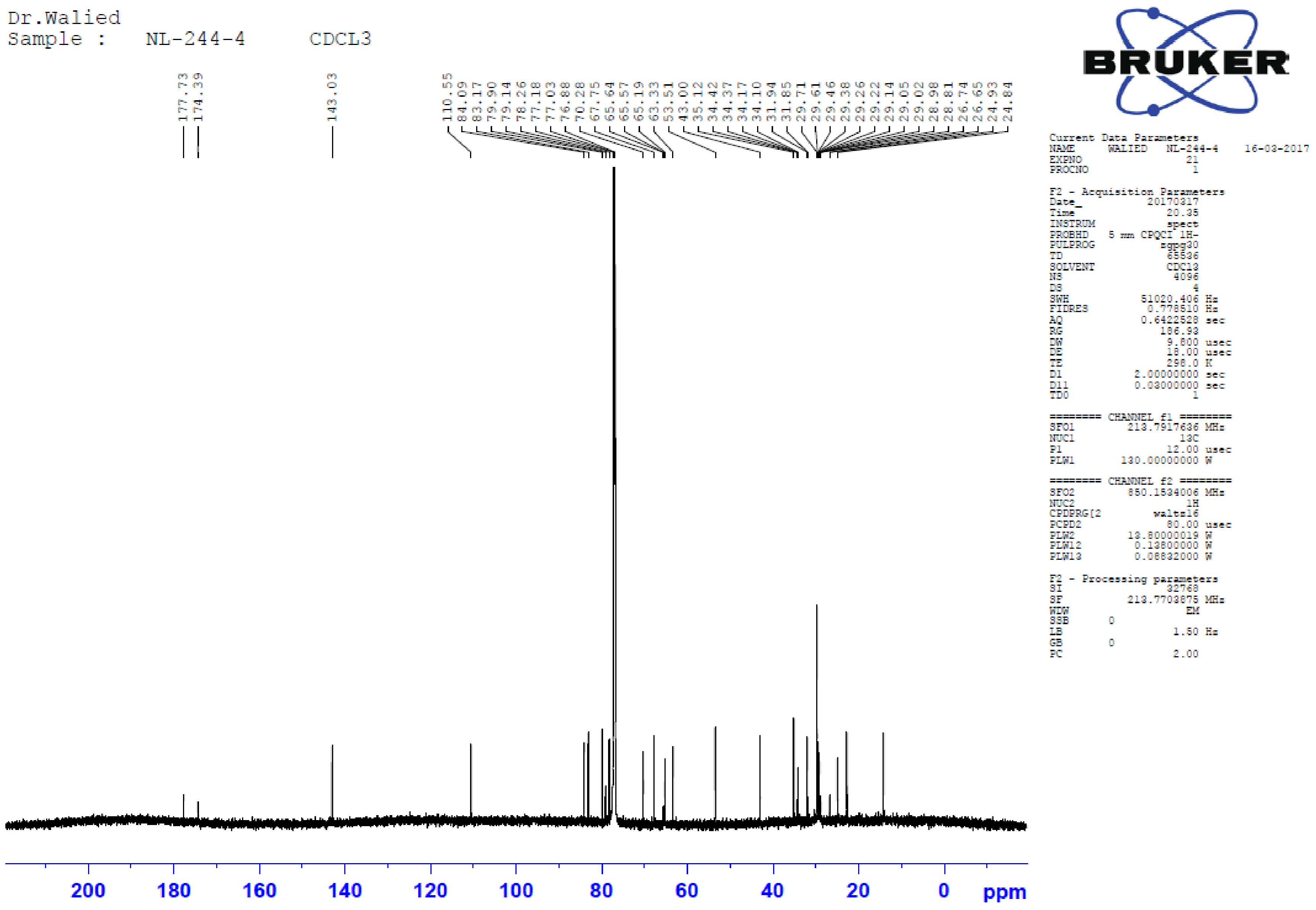

Figure S3e: ^{13}C NMR of compound 3

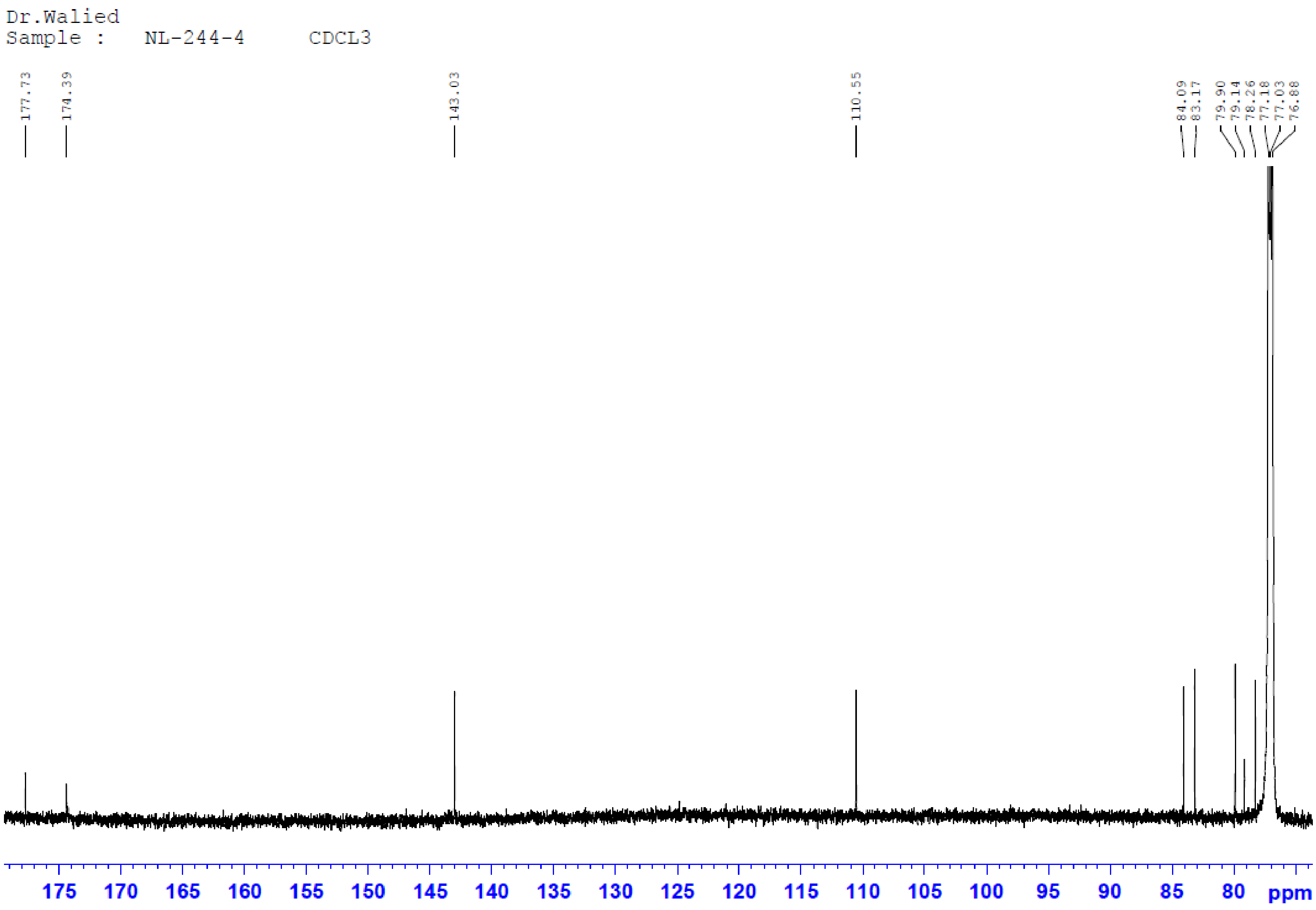

Figure S3f: ^{13}C NMR of compound 3

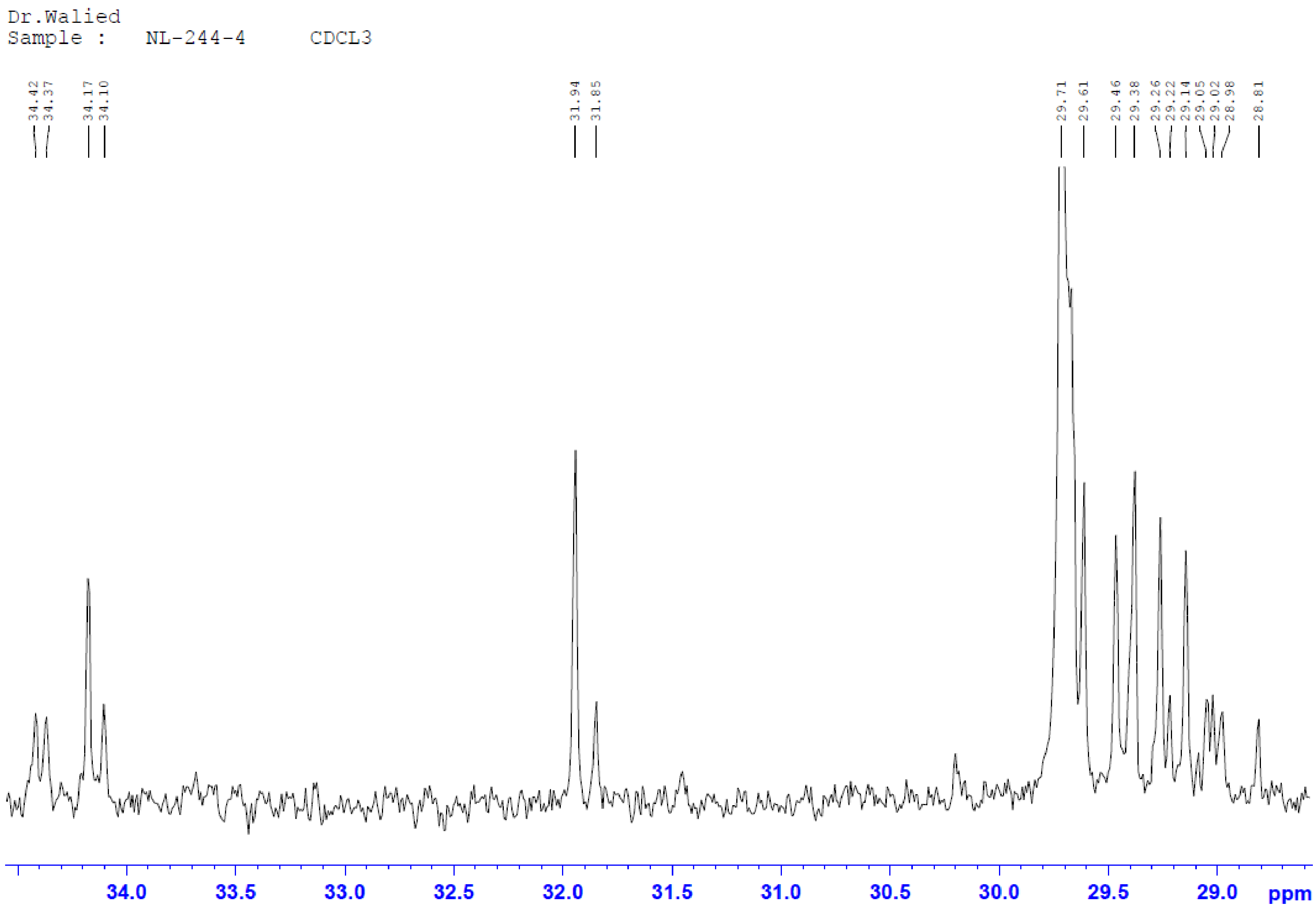

Figure S3g: ^{13}C NMR of compound 3

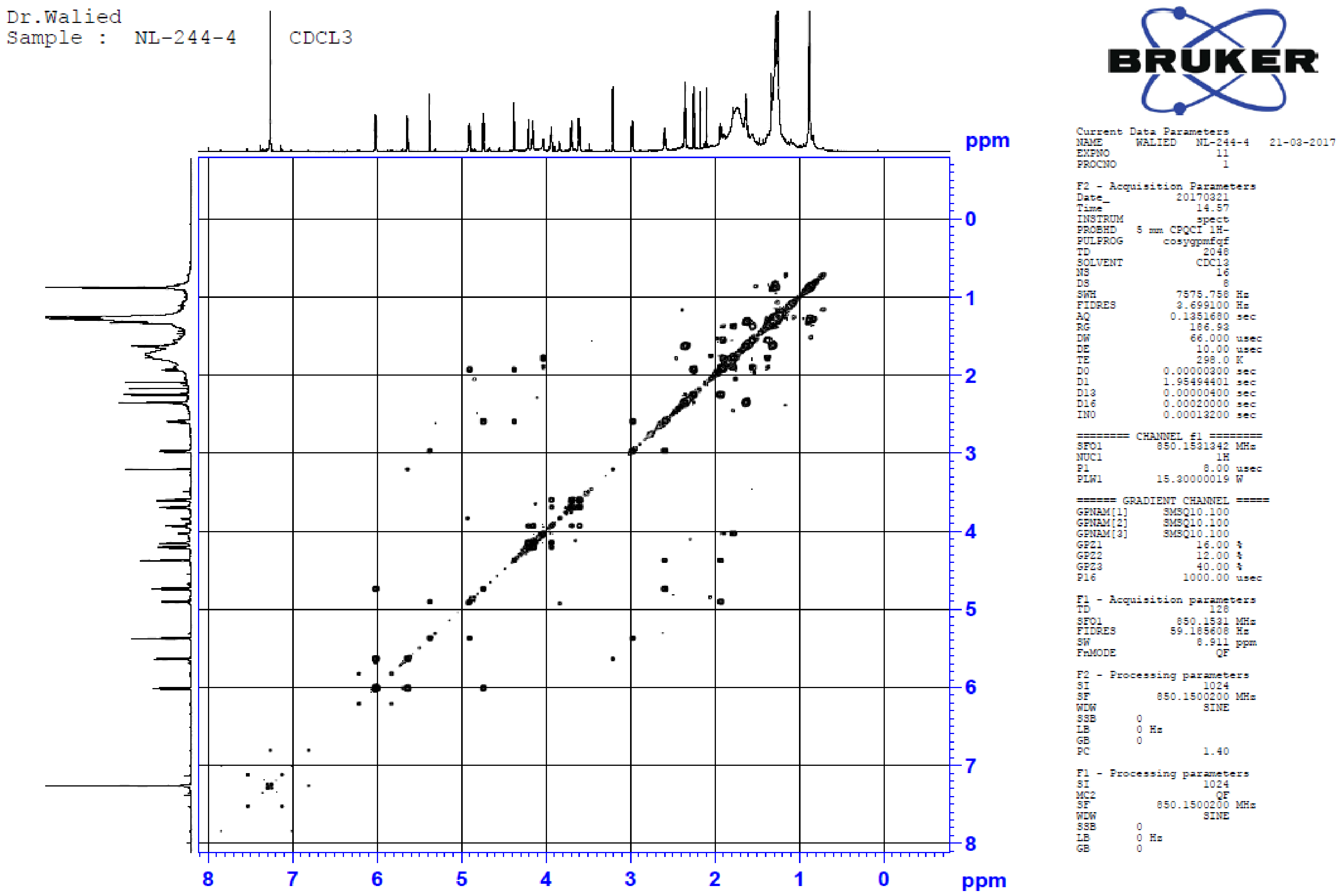

Figure S3h: COSY NMR of compound 3

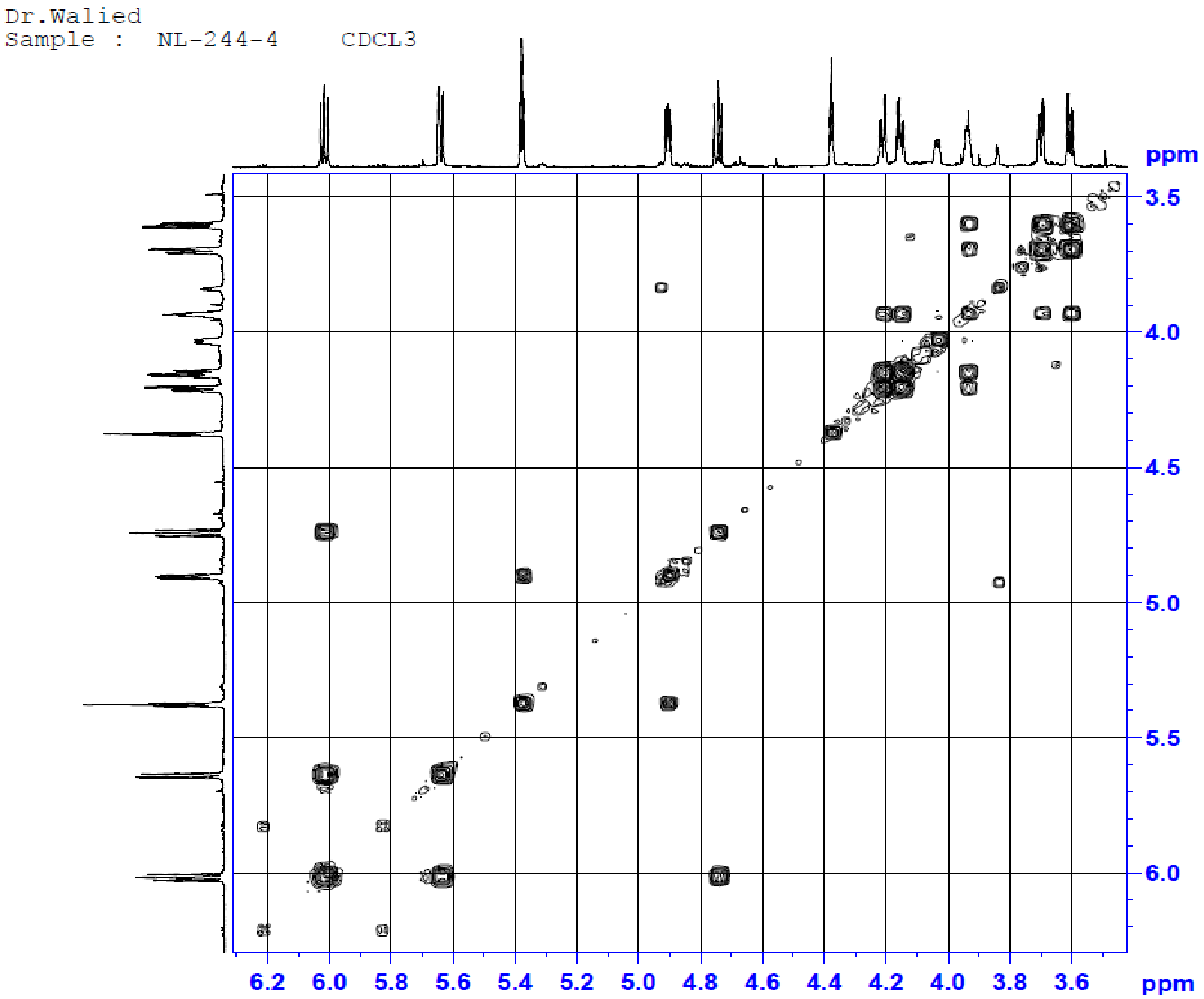

Figure S3i: COSY NMR of compound 3

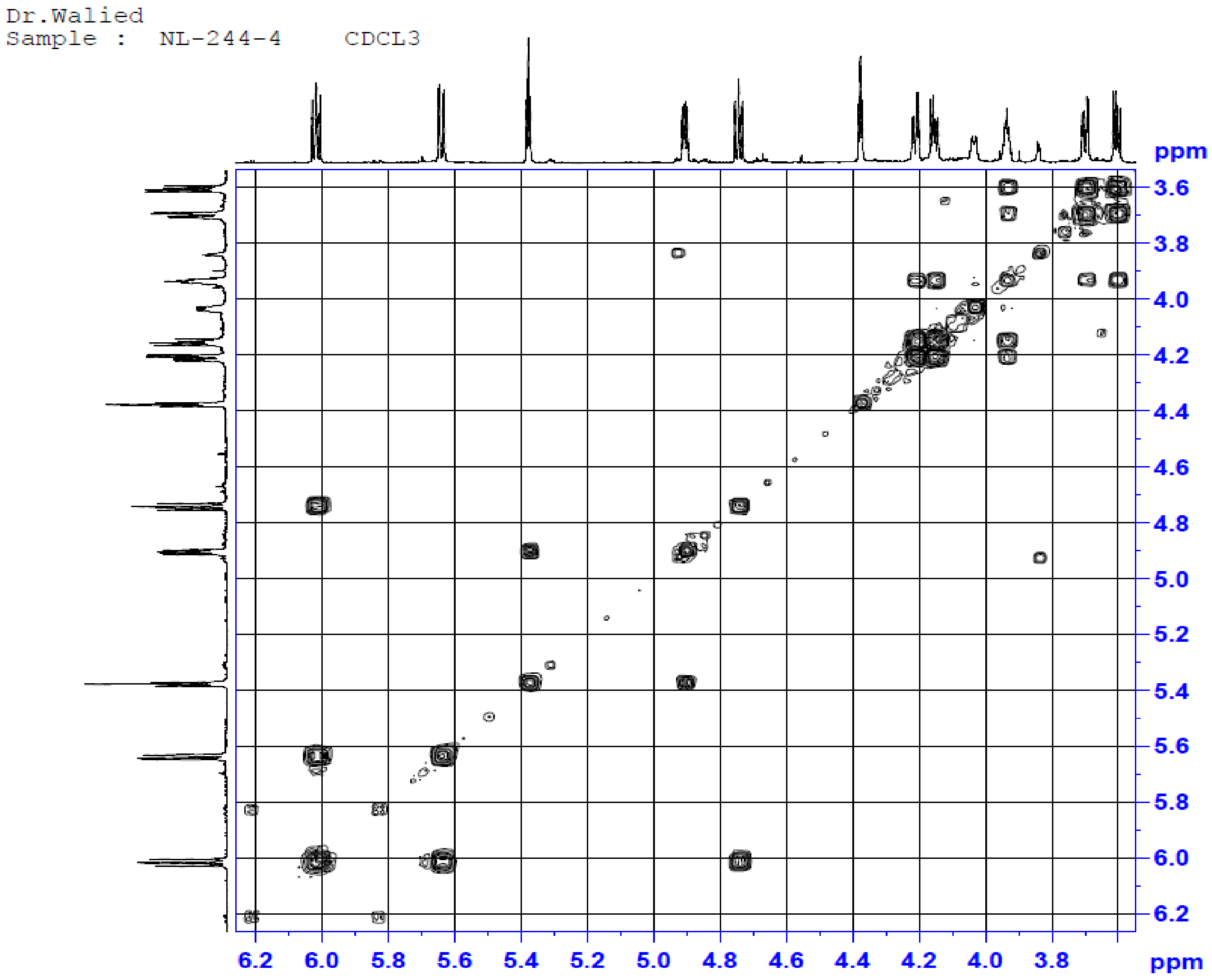

Figure S3j: COSY NMR of compound 3

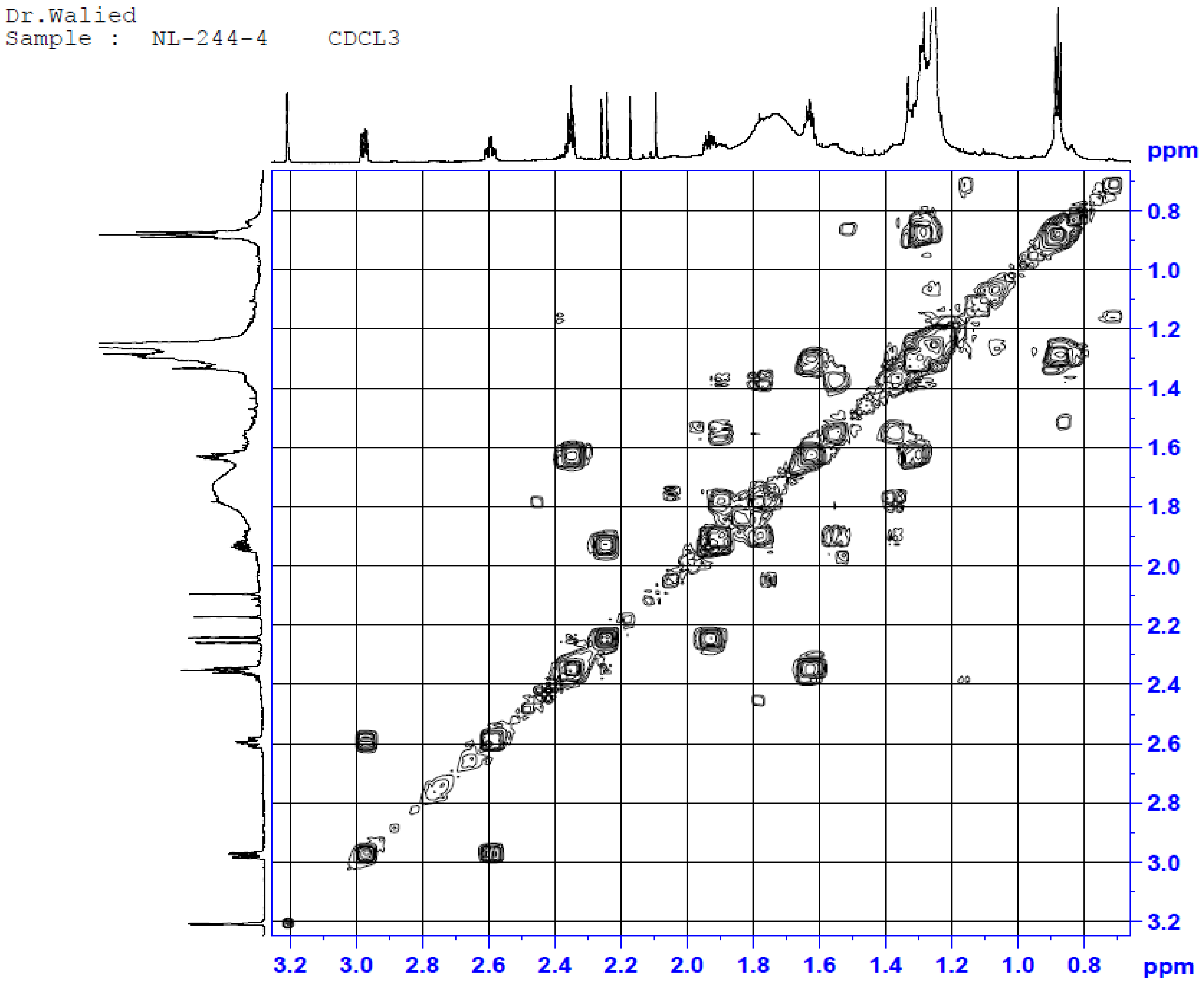

Figure S3k: COSY NMR of compound 3

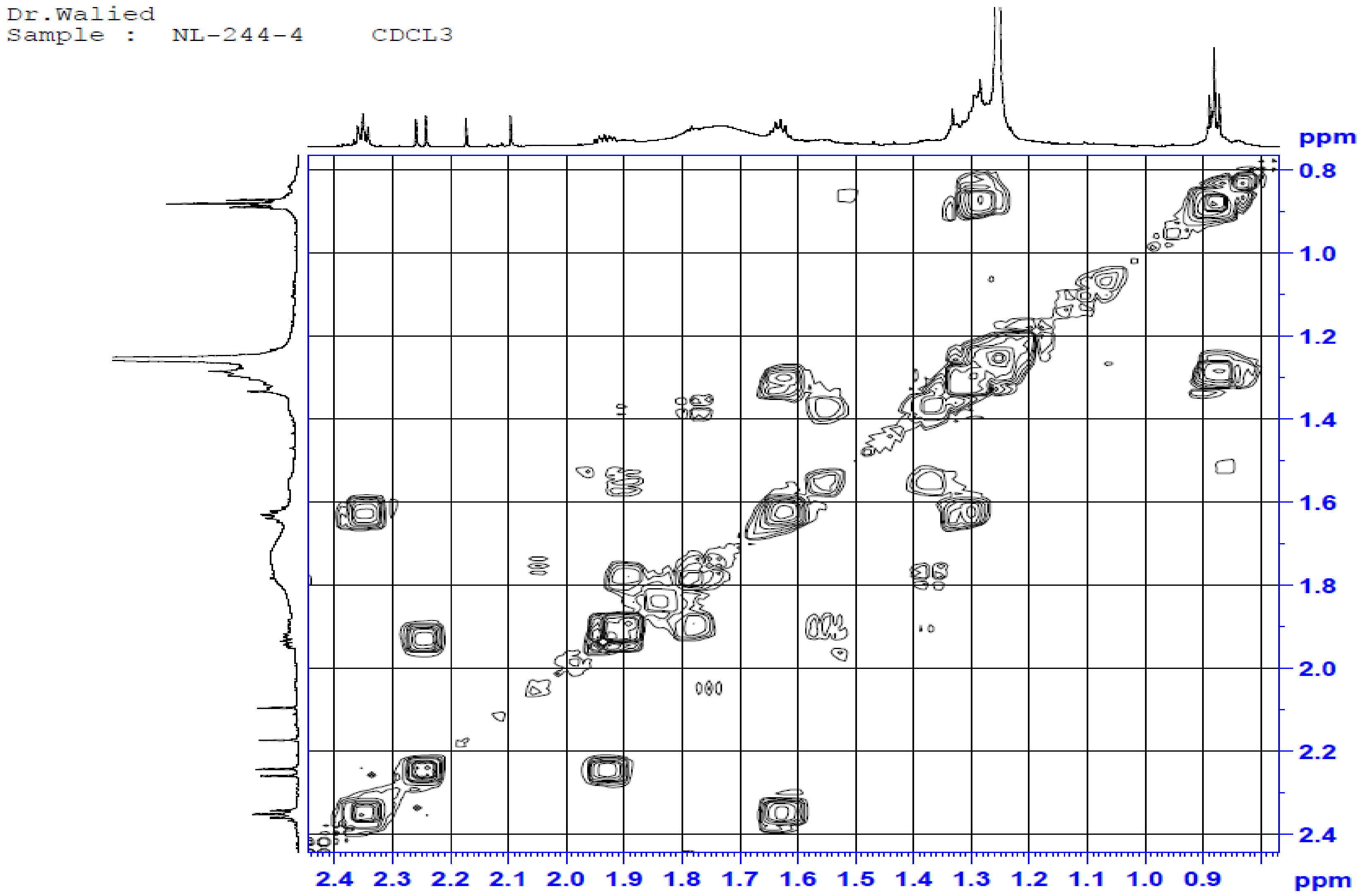

Figure S3l: COSY NMR of compound 3

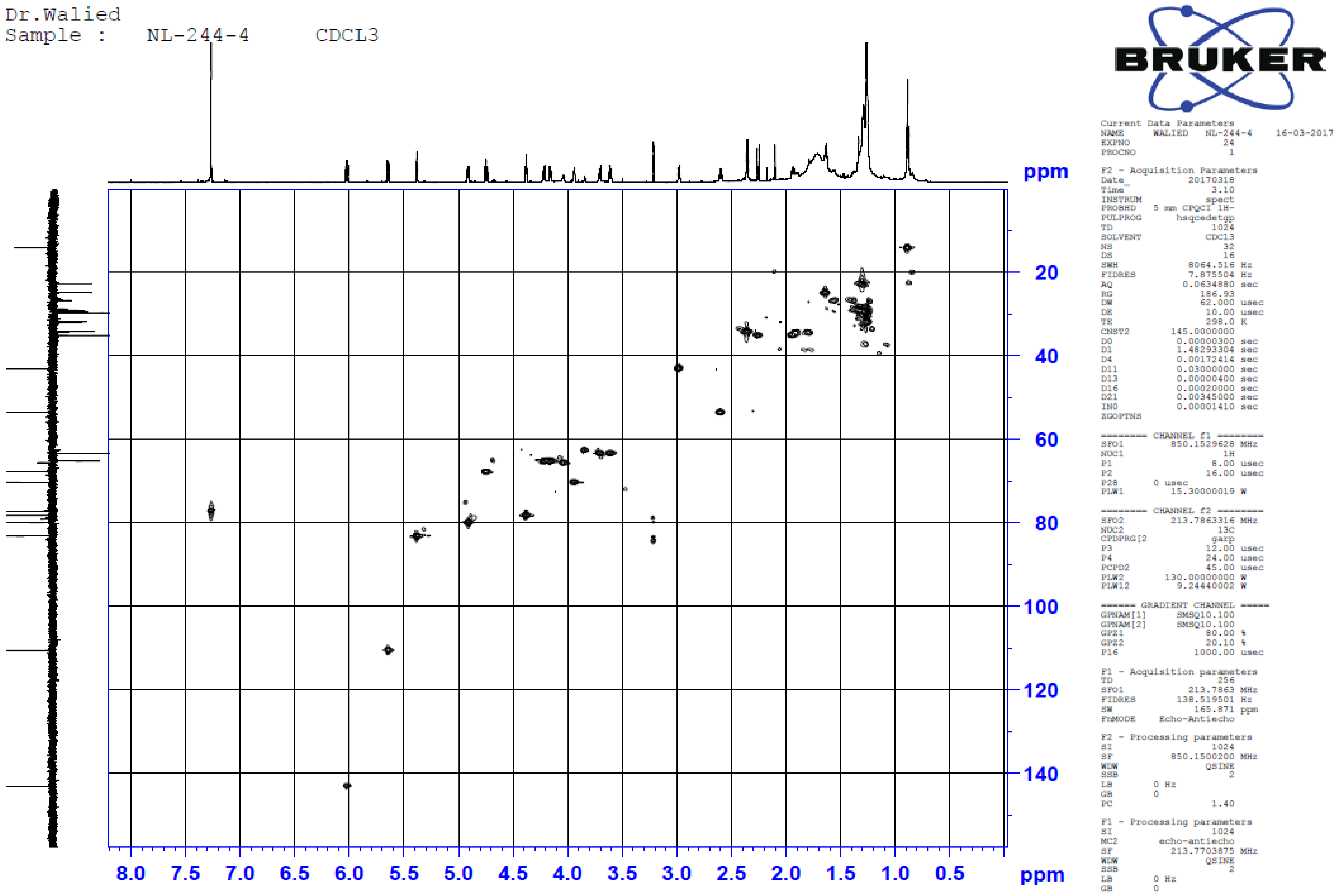

Figure S3m: HSQC NMR of compound 3

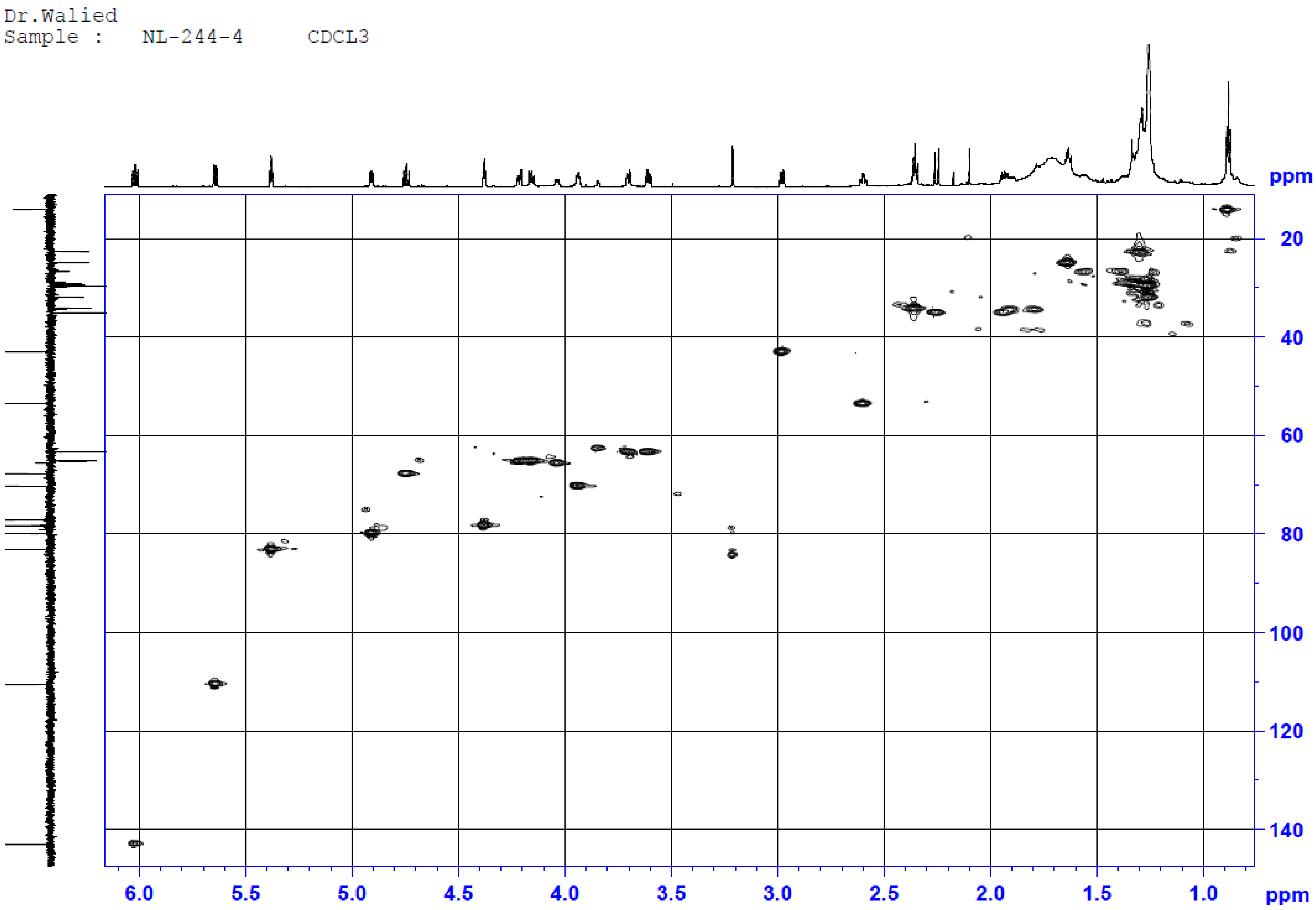

Figure S3n: HSQC NMR of compound 3

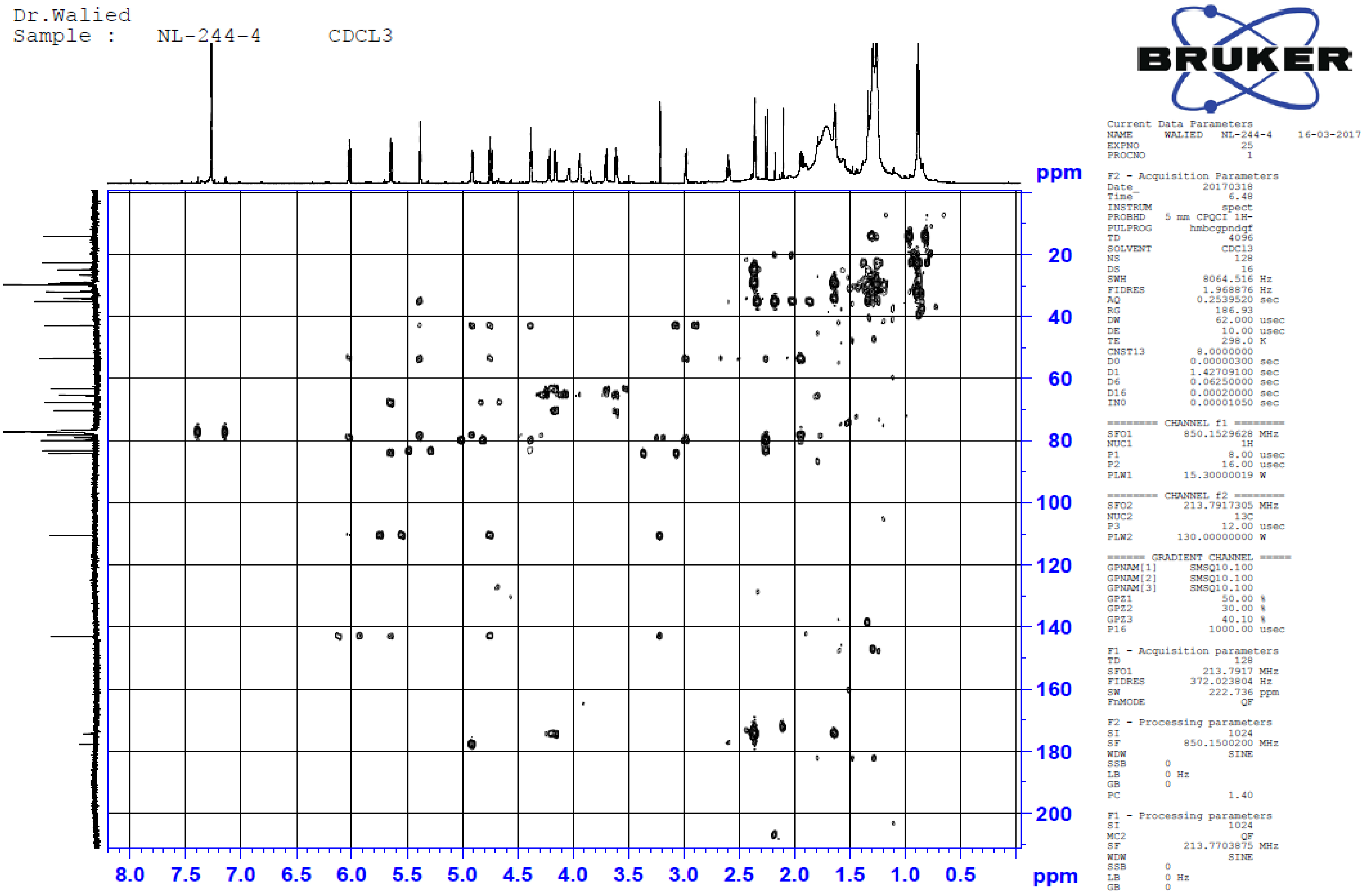

Figure S3o: HMBC NMR of compound 3

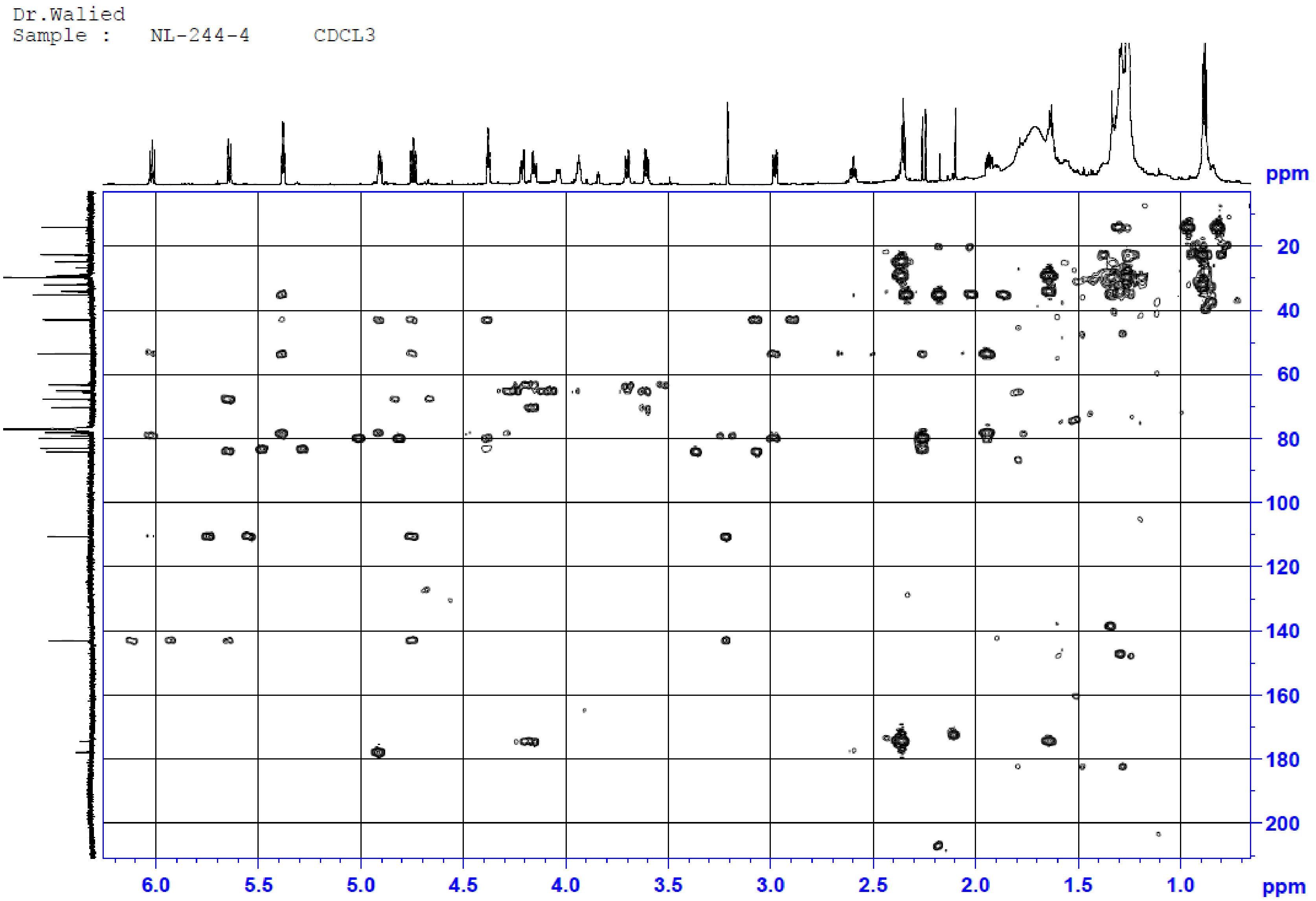

Figure S3p: HMBC NMR of compound 3

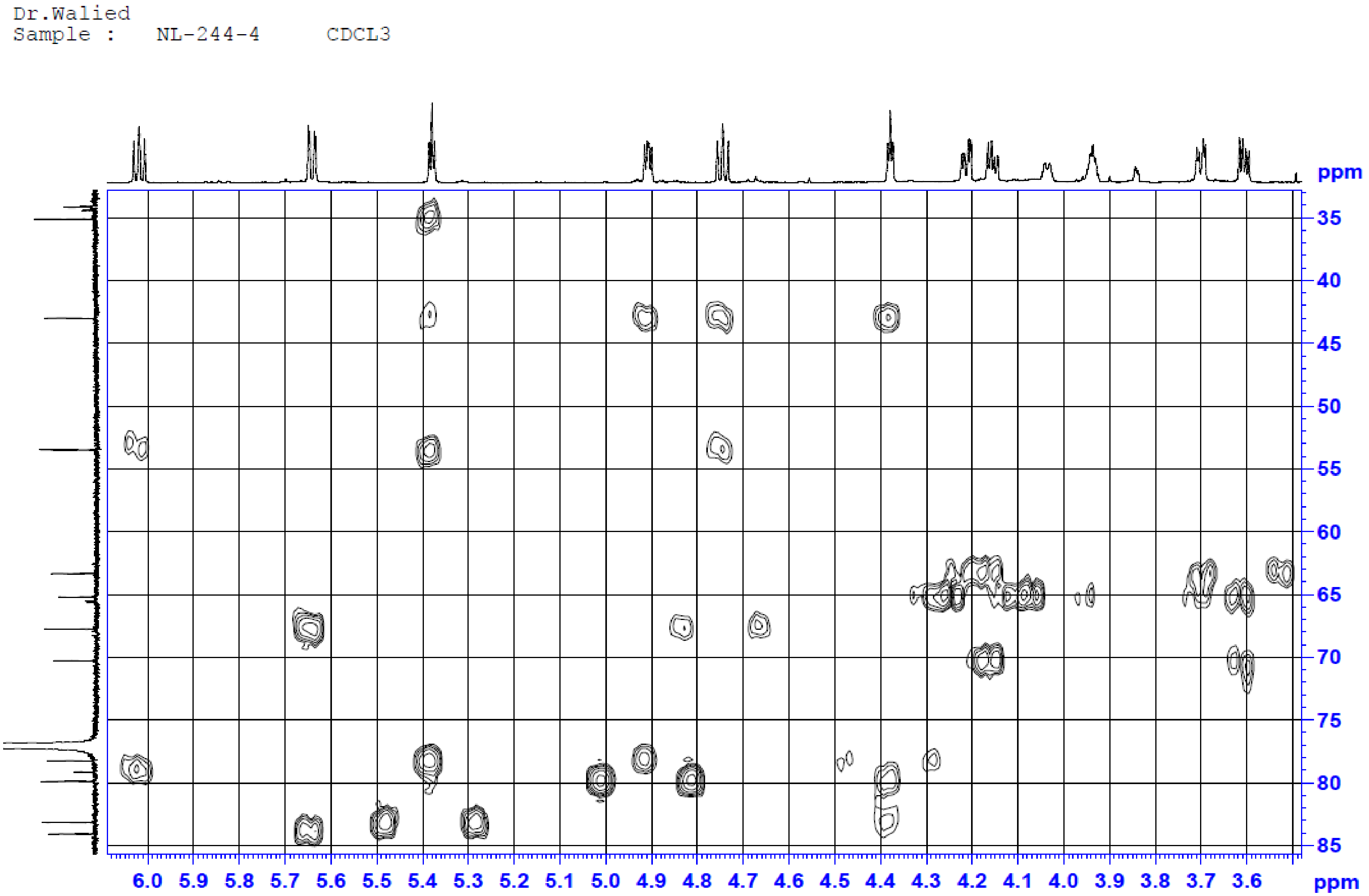

Figure S3q: HMBC NMR of compound 3

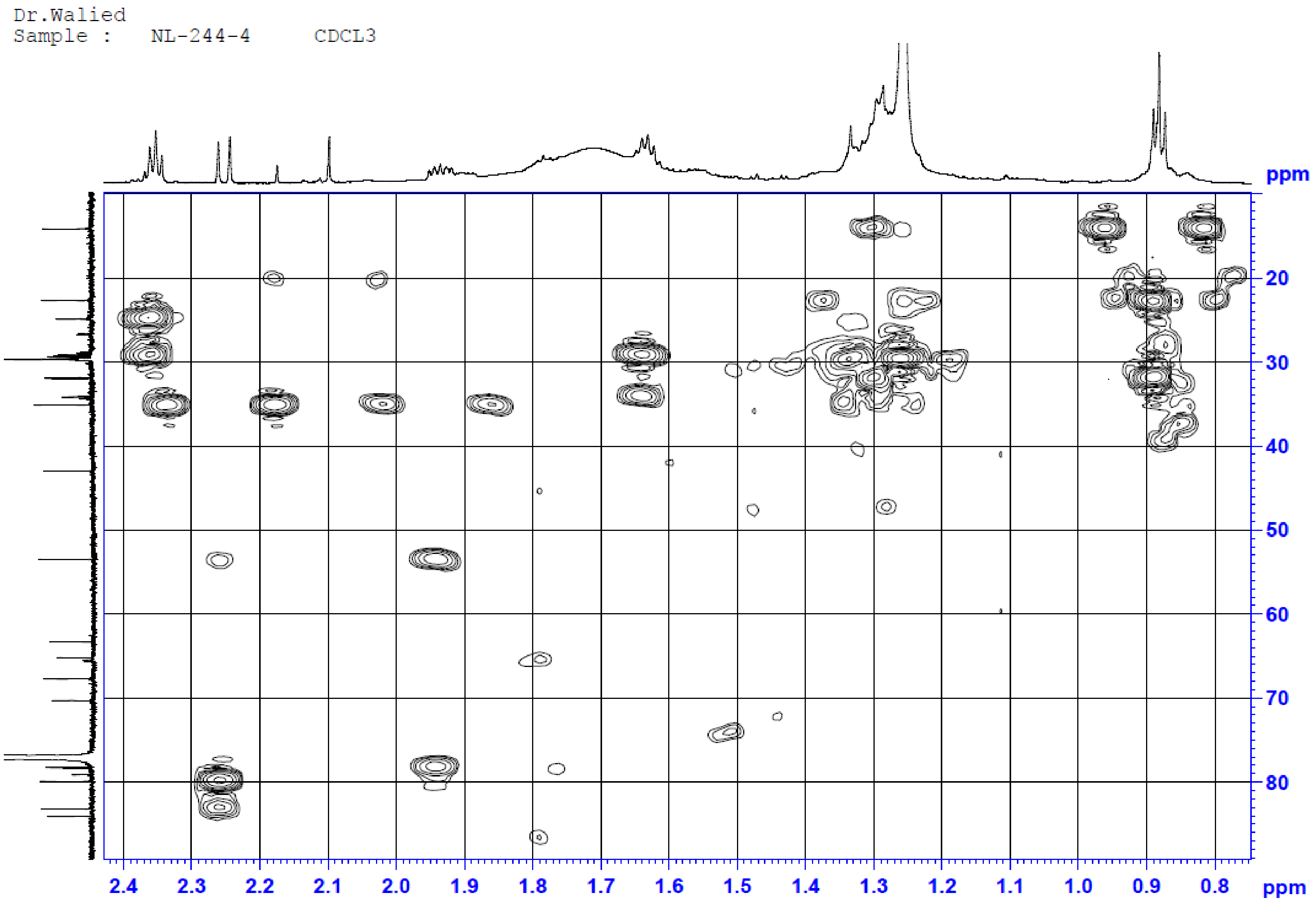

Figure S3r: HMBC NMR of compound 3

Dr.Walied
Sample :   NL-244-4   CDCL3

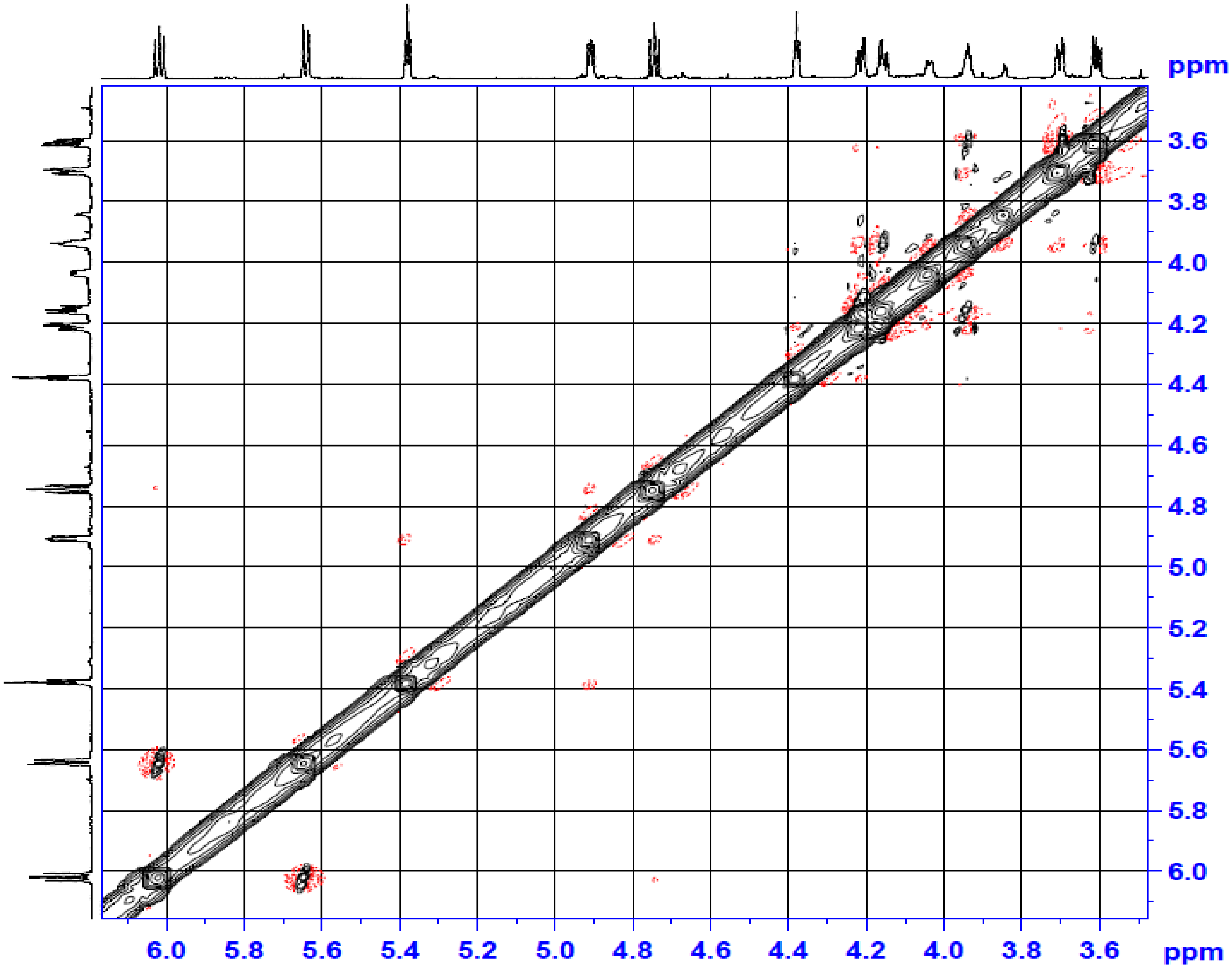

Figure S3s: NOSY NMR of compound 3

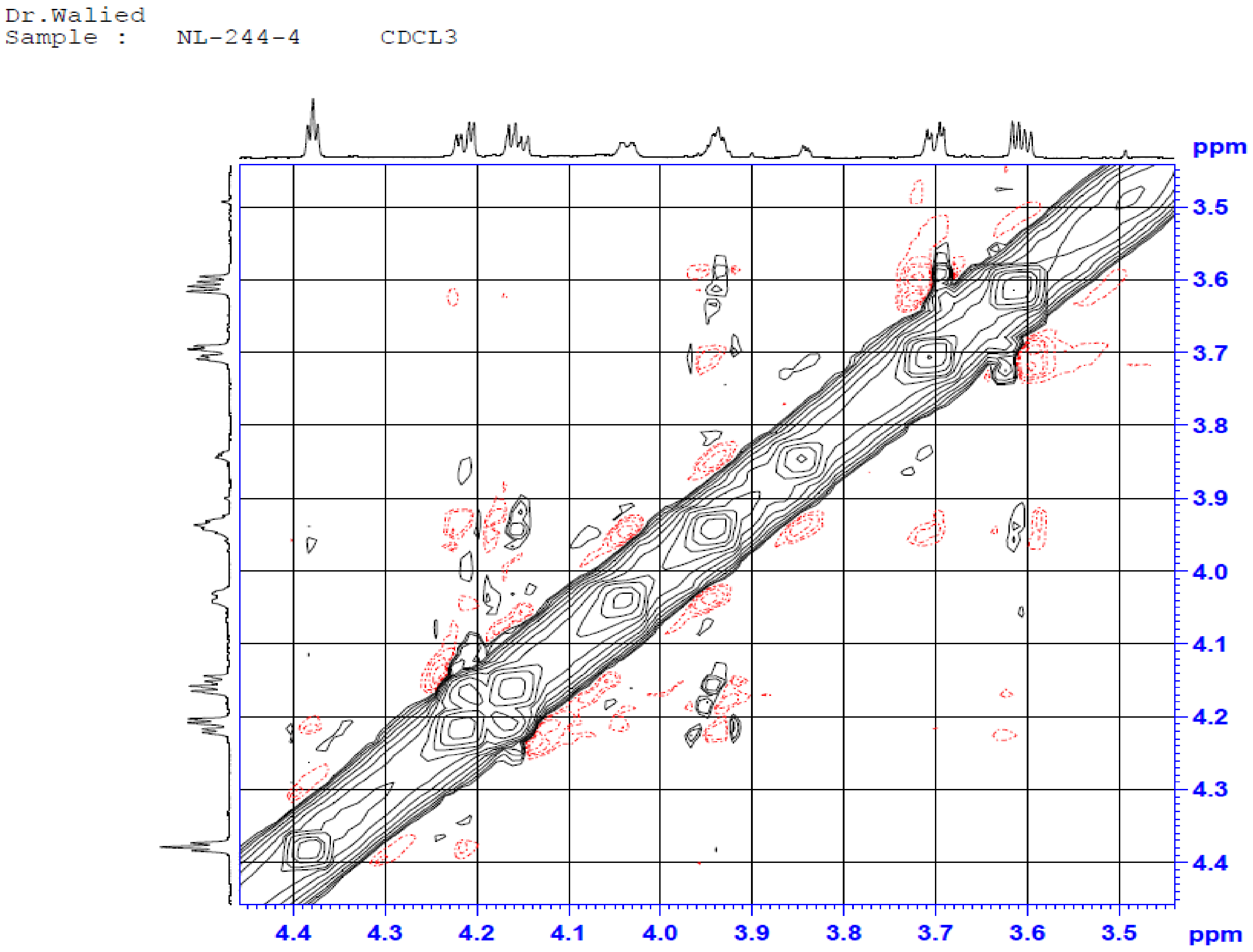

Figure S3t: NOSY NMR of compound 3
